# Cas9-mediated genome editing reveals a significant contribution of calcium signaling pathways to anhydrobiosis in Pv11 cells

**DOI:** 10.1038/s41598-021-98905-w

**Published:** 2021-10-05

**Authors:** Yugo Miyata, Hiroto Fuse, Shoko Tokumoto, Yusuke Hiki, Ruslan Deviatiiarov, Yuki Yoshida, Takahiro G. Yamada, Richard Cornette, Oleg Gusev, Elena Shagimardanova, Akira Funahashi, Takahiro Kikawada

**Affiliations:** 1 Division of Biomaterial Sciences, Institute of Agrobiological Sciences, National Agriculture and Food Research Organization (NARO), Tsukuba, Japan; 2 Department of Integrated Biosciences, Graduate School of Frontier Sciences, The University of Tokyo, Kashiwa, Chiba Japan; 3 Department of Biosciences and Informatics, Keio University, Yokohama, Kanagawa Japan; 4 Extreme Biology Laboratory, Institute of Fundamental Medicine and Biology, Kazan Federal University, Kazan, Russia; 5 Institute for Advanced Biosciences, Keio University, Tsuruoka, Yamagata Japan; 6 Systems Biology Program, Graduate School of Media and Governance, Keio University, Fujisawa, Kanagawa Japan; 7 Laboratory for Transcriptome Technology, RIKEN Center for Integrative Medical Sciences, RIKEN, Yokohama, Kanagawa Japan

**Keywords:** Calcium signalling, Genetic engineering

## Abstract

Pv11 is an insect cell line established from the midge *Polypedilum vanderplanki*, whose larval form exhibits an extreme desiccation tolerance known as anhydrobiosis. Pv11 itself is also capable of anhydrobiosis, which is induced by trehalose treatment. Here we report the successful construction of a genome editing system for Pv11 cells and its application to the identification of signaling pathways involved in anhydrobiosis. Using the Cas9-mediated gene knock-in system, we established Pv11 cells that stably expressed GCaMP3 to monitor intracellular Ca^2+^ mobilization. Intriguingly, trehalose treatment evoked a transient increase in cytosolic Ca^2+^ concentration, and further experiments revealed that the calmodulin–calcineurin–NFAT pathway contributes to tolerance of trehalose treatment as well as desiccation tolerance, while the calmodulin–calmodulin kinase–CREB pathway conferred only desiccation tolerance on Pv11 cells. Thus, our results show a critical contribution of the trehalose-induced Ca^2+^ surge to anhydrobiosis and demonstrate temporally different roles for each signaling pathway.

## Introduction

Anhydrobiosis is the striking ability of some organisms to survive extreme desiccation. To date, among known anhydrobiotic animals, the sleeping chironomid *Polypedilum vanderplanki* is the only species from which culturable cell lines have been established^1,2^. The cell line, Pv11, derived from embryos of the midge, recapitulates the extreme desiccation tolerance of *P. vanderplanki* larvae^3^. Thus, Pv11 cells are a promising model for investigating the molecular mechanisms of anhydrobiosis in *P. vanderplanki*. However, a major experimental limitation has been that only a limited number of gene engineering tools are available in Pv11 cells^4,5^.

Larvae of *P. vanderplanki* inhabit temporary rock pools in semi-arid regions of sub-Saharan Africa^6^. During the drought season, these rock pools completely dry up and consequently any larvae they contain are also dehydrated. During this process, the larvae sense the onset of desiccation and start to synthesize large quantities of the disaccharide trehalose in the fat body tissues. Trehalose is then distributed throughout the whole larval body and protects biological molecules from desiccation damage by replacing intracellular water with a biological glass^6^. Over the last few decades, physiological studies, and more recently genomics and metabolomics approaches, have revealed the metabolic processes and genes involved in anhydrobiosis^6,7^.

To induce anhydrobiosis in Pv11 cells, pre-treatment with highly concentrated trehalose is necessary^3^. Such high concentrations of trehalose are generally cytotoxic to insect cell lines^3^, but Pv11 cells do not die; instead, they are induced to undergo anhydrobiosis. During this pre-treatment, Pv11 cells take up trehalose for the protection of intracellular biological molecules against desiccation. In addition, trehalose treatment changes gene expression profiles in Pv11 cells^8,9^, suggesting that trehalose is also a signal that regulates gene expression. However, the exact mechanisms of anhydrobiosis in Pv11 cells and how they regulate gene expression during trehalose treatment have yet to be elucidated.

The CRISPR/Cas9 system is a versatile technology for genome editing due to its ease of use and accuracy, and its successful application to a wide range of species^10^. Basically, the system is composed of two molecules, Cas9 nuclease and a guide RNA (gRNA), which form a complex that makes a double strand break (DSB) at a target DNA site. When DNA fragments that are identical (or very similar) to sequences around the DSB are present, this exogenous DNA is efficiently inserted into the DSB site by intrinsic DNA repair mechanisms^11,12^. This knock-in technology has been used in various species including non-model organisms^13–16^. If a CRISPR/Cas9-based genome editing system can be developed for Pv11 cells, it should improve the experimental feasibility of exogenous gene expression and lead to the unravelling of the molecular details of anhydrobiosis in *P. vanderplanki*.

Genetically encoded fluorescent sensors are widely used to visualize molecular events and the mobilization of signaling molecules in living cells^17^. Intracellular Ca^2+^ mobilization is frequently analyzed with genetically encoded calcium indicators (GECIs), which were the first fluorescent sensors of this type to be developed. Since their development, GECIs have been improved by many researchers^18^, and are ideal for identifying biological factors that cause intracellular Ca^2+^ changes and activation of Ca^2+^ signaling pathways^19–23^. Given that Ca^2+^ signaling pathways are activated in response to various stresses, including oxidative stress^24,25^, heat shock^26^, osmotic stress^27^, and ER stress^28,29^, GECIs should provide clear information on the involvement of Ca^2+^ signaling pathways in anhydrobiosis in Pv11 cells.

Ca^2+^ is an intracellular second messenger that regulates a large number of biological processes via signaling pathways and the modulation of gene expression^30,31^. The key Ca^2+^-dependent signaling pathways are calmodulin (CaM)–calcineurin (CaN)–nuclear factor of activated T-cells (NFAT) and CaM–Ca^2+^/calmodulin-dependent protein kinase (CaMK)–calcium/cAMP response element binding protein (CREB). CaM is a Ca^2+^ binding protein that transduces a change in intracellular Ca^2+^ concentration into molecular signals. CaN is a phosphatase activated by Ca^2+^/CaM complexes, which in turn dephosphorylates and activates NFAT. Activated NFAT binds to the consensus DNA sequence, TTTCCA, and regulates transcription of any downstream genes^32^. Ca^2+^/CaM complex-activated CaMK phosphorylates CREB, which binds to the canonical CRE sequence, TGACGT, and regulates downstream gene expression^33^. The signaling pathways are conserved in many cell types^30,34–36^ and across species^37–40^, and are activated by many stimuli^24–29^. Therefore, we hypothesized that Ca^2+^ signaling pathways may contribute to the induction of anhydrobiosis in *P. vanderplanki*.

Here we describe the development of a CRISPR/Cas9-based genome editing system for Pv11 cells and its application to the identification of signaling pathways required for anhydrobiosis. Using the Cas9-mediated gene knock-in system, exogenous genes were inserted into a target site and constitutively expressed without affecting anhydrobiosis. We applied the gene knock-in method to visualize the mobilization of the second messenger, Ca^2+^, by knocking in the gene for the GECI, GCaMP3. Significantly, trehalose treatment evoked a transient increase in cytosolic Ca^2+^ concentration in Pv11 cells, and inhibition of Ca^2+^ signaling pathways during trehalose treatment decreased the cell survival rate after rehydration. Inhibitor experiments further indicated that the CaM–CaN–NFAT pathway conferred tolerance to trehalose treatment as well as desiccation tolerance on Pv11 cells, while the CaM–CaMK–CREB pathway conferred only desiccation tolerance. These results show the critical contribution of the trehalose-induced Ca^2+^ surge to anhydrobiosis and emphasise the distinct roles of the two signaling pathways.

## Results

### Utility of the CRISPR/Cas9 system in Pv11 cells

First, to examine whether the CRISPR/Cas9 system would work in Pv11 cells, the Cas9-expression vector and synthetic gRNAs targeting AcGFP1 genes were transfected into Pv11-KH cells; this Pv11-derived cell line stably expresses a transfected AcGFP1 construct^4^ (Fig. S1). After 4 weeks, images of the cells were acquired (Fig. S1a), and flow cytometric analysis was carried out, showing that AcGFP1 fluorescence was lost in about 30% of cells (Fig. S1b). GFP-negative cells were sorted, and the sequence of the AcGFP1 gene in the genome of these cells was analyzed. As shown in Fig. S1c,d, insertion or deletion (indel) mutations were found both in gRNA#1- and #3-transfected cells (Fig. S1c,d). Together, these data show that the CRISPR/Cas9 system can cause targeted indel mutations in Pv11 cells.

### Construction of functional gRNA-expression vectors for Pv11 cells

To construct gRNA-expression vectors for Pv11 cells, the promoter region of the PvU6b gene was cloned (Supplementary Data 1). To overcome the experimental limitation that U6 promoters generally initiate transcription at G or A^41–43^, DmtRNA sequence was added at the + 1 position of the PvU6b promoter as described in the tRNA-flanked gRNA expression system^44,45^ (Supplementary Data 1). The gRNA- and Cas9-expression vectors were transfected into Pv11-KH cells, and after 4 weeks, cell images and flow cytometric data were acquired (Figs. S2a,b). As in the case of the synthetic gRNAs (Fig. S2), AcGFP1 fluorescence was lost in about 30% of the cells transfected with both Cas9- and gRNA-expression vectors (Fig. S2b). Again, indel mutations were found in the fluorescence-negative cells (Fig. S2c,d).

### CRISPR/Cas9-mediated targeted knock-in for exogenous gene expression

**Figure 1:**
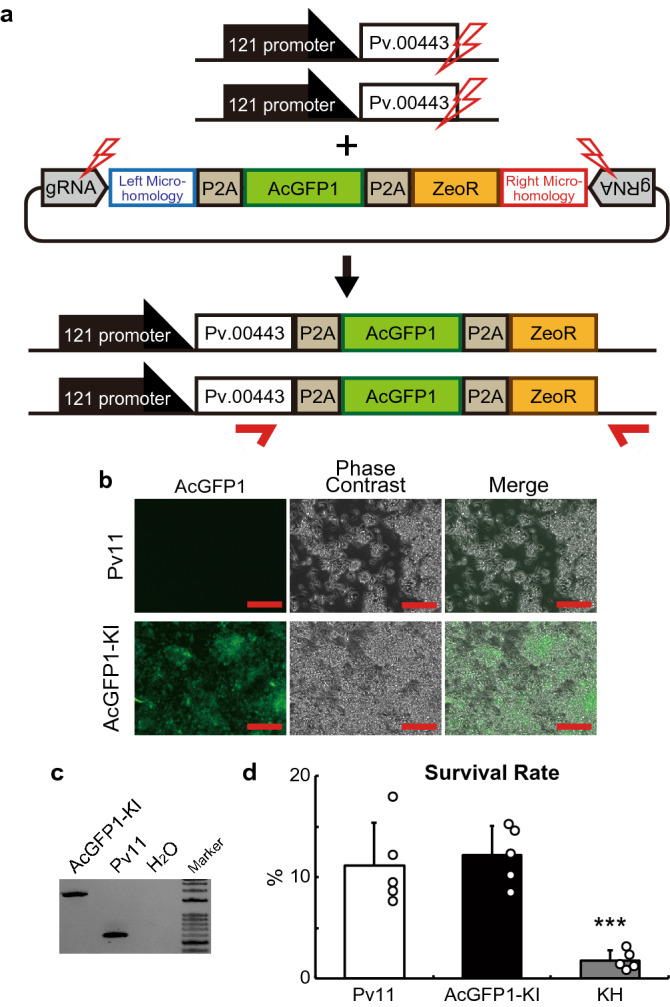
Insertion of the AcGFP1 and zeocin resistance (ZeoR) genes into the 5′ flanking site of the stop codon of the *Pv.00443* gene. (**a**) The PITCh scheme for AcGFP1 and ZeoR knock-in is shown; the Cas9- and gRNA-expression vectors and the donor vector harboring AcGFP1 and ZeoR genes were transfected into Pv11 cells. Red arrows indicate the primers used in genomic PCR with gDNA obtained from cells after selection for zeocin resistance and AcGFP1 fluorescence. (**b**) Cell images were acquired with a conventional fluorescence microscope. (**c**) Genomic PCR from the cells was carried out (the whole gel image is in Supplementary Fig. S3). (**d**) Comparison of survival rate after desiccation-rehydration treatment of two cell lines stably expressing AcGFP1: targeted knock-in (AcGFP1-KI) and random integration-based (Pv11-KH) cells. An endogenous strong promoter, the 121 promoter, is located in the upstream region of the *Pv.00443* gene. Scale bars, 100 µm. Values are expressed as mean ± SD; n = 5 in each group. ***p = 0.0008 (vs. Pv11) and 0.0003 (vs. AcGFP1-KI).

Targeted knock-in for exogenous gene expression is one of the most useful applications of the CRISPR/Cas9 system. To develop the knock-in technique in Pv11 cells, we tried the PITCh (Precise Integration of Target Chromosome) method^11^ and selected the *Pv.00443* gene as the insertion site because its expression is constitutively high under the regulation of a strong constitutive promoter, the 121 promoter, in Pv11 cells^5^. To exploit the endogenous constitutive expression system, we designed the gRNA to target the 5′-flanking site of the stop codon of *Pv.00443*, and then constructed a donor vector for the polycistronic expression of three genes: *Pv.00443*, AcGFP1 and the zeocin resistance gene (ZeoR; Fig. 1a and Supplementary Data 2).

The Cas9- and gRNA-expression vectors plus the donor vector were transfected into Pv11 cells, and the cells were treated with zeocin. After zeocin selection, single-cell sorting for AcGFP1 fluorescence was performed, and the sorted cell was grown on a feeder layer of intact Pv11 cells for 2 weeks. Zeocin treatment and sorting were carried out again to remove the feeder cells. Genomic DNA was extracted from the surviving, sorted cells and subjected to PCR using primers flanking the inserted DNA (Fig. 1b). As shown in Fig. 1c and Fig. S3, only a single 1840 bp-band was detected in the modified cells, which means that biallelic integration of the donor construct occurred (Fig. 1a,c and Fig. S3). Furthermore, sequencing of the band confirmed a precise gene knock-in in the target cells (Supplementary Data 3). The established cell line was named AcGFP1-KI.

As mentioned above, the Pv11-KH cell line was established by random integration of an AcGFP1 expression construct^4^. Pv11-KH cells exhibited a lower desiccation tolerance compared to intact Pv11 cells, perhaps due to unintended disruption of desiccation-related genes by genomic insertion of exogenous DNA fragments^46,47^, as previously noted^4^ and shown in Fig. 1d (Fig. 1d). To assess whether there was an advantage of the targeted knock-in system over the random integration of transfected DNA, the survival rate after a desiccation-rehydration cycle was compared in two different AcGFP1-expressing cell lines, AcGFP1-KI and Pv11-KH. As shown in Fig. 1d, there was no significant difference in the survival rate between intact Pv11 and AcGFP1-KI cells, while the survival rate of Pv11-KH cells was significantly lower than the other two cell lines (p = 0.0008 for Pv11 and 0.0003 for AcGFP1-KI; Fig. 1d). This result clearly shows that the targeted knock-in into the 5′-flanking site of the stop codon of *Pv.00443* caused no deleterious effect on the anhydrobiotic ability of Pv11 cells.

To test whether different donor DNAs could be inserted into each of the two *Pv.00443* alleles simultaneously, the HaloTag and hygromycin resistance (HygR) genes were individually cloned into a donor vector and transfected with the Cas9- and gRNA-expression vectors into Pv11 cells (Fig. S4a). After hygromycin treatment, single-cell sorting was performed by staining with HaloTag fluorescent ligand, and the sorted cells were grown with intact Pv11 cells as a feeder layer for 2 weeks. Hygromycin treatment and sorting were carried out again to remove feeder cells, and cell images were acquired (Fig. S4b). The genome of the cells was extracted and subjected to genomic PCR using primers flanking the inserted DNA. As shown in Fig. S4c, 1139 bp- and 1280-bp bands were detected, suggesting genomic insertion of both the HaloTag and HygR genes, respectively, had occurred (Fig. S4c). Furthermore, sequencing of the PCR bands confirmed precise gene knock-in of both genes in Pv11 cells (Supplementary Data 4 and 5). These results indicate the successful knock-in of a different donor DNA into each *Pv.00443* allele of homologous chromosomes. This experiment also demonstrates that both inserted genes are haplosufficient, i.e. a single copy of each exogenous gene gives sufficient protein expression for the desired phenotype. Again, there was no significant difference in the anhydrobiotic survival rate of intact Pv11 and the HaloTag/HygR-KI cells (p = 0.8929, Fig. S4d).

### Establishment of GCaMP3-KI cells

**Figure 2:**
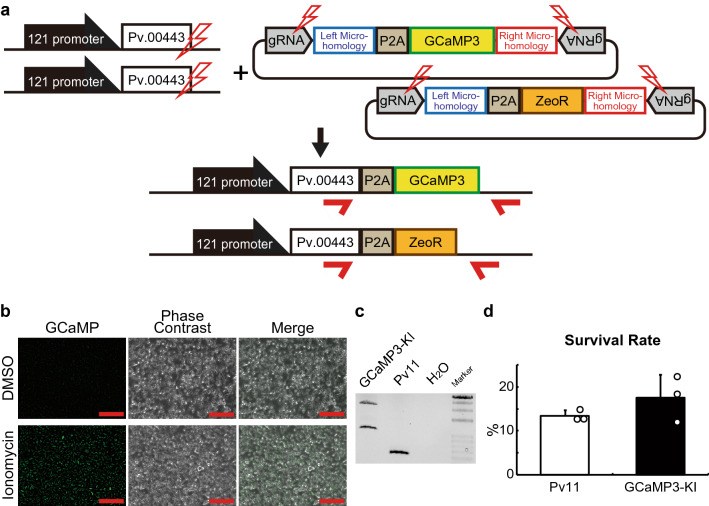
The establishment of a cell line stably expressing GCaMP3. (**a**) The PITCh scheme for GCaMP3 and ZeoR knock-in is shown; Cas9- and gRNA-expression vectors and two donor vectors harboring a gene encoding GCaMP3 or ZeoR were transfected into Pv11 cells. Red arrows indicate the primers used in genomic PCR with gDNA obtained from cells after selection for zeocin resistance and GCaMP3 fluorescence. (**b**) Cell images following treatment with DMSO or ionomycin were acquired by a conventional fluorescence microscope. (**c**) Genomic PCR was carried out on cell DNA (the whole gel image is shown in Supplementary Fig. S6). (**d**) Comparison of survival rate after desiccation-rehydration treatment of Pv11 and GCaMP3-KI cells. Scale bars, 100 µm. Values are expressed as mean ± SD; n = 3 in each group.

To use the genome editing technique to investigate signaling mechanisms involved in anhydrobiosis, we attempted to knock-in the GCaMP3 gene, which is one of GECIs used in a wide range of cell types^20^, including insect cells^48,49^. As shown in Fig. 2a, the Cas9- and gRNA-expression vectors plus two different donor vectors harboring GCaMP3 and ZeoR were transfected into Pv11 cells (Fig. 2a). To select cells expressing both genes, zeocin treatment and subsequent single cell sorting were performed (Fig. S5). We identified a clonal cell line that exhibited strong GCaMP3 fluorescence after treatment with the calcium ionophore, ionomycin, compared to DMSO (Fig. 2b). The genome of the cells was extracted, and subsequent genomic PCR showed both 2035 bp- and 1057 bp-bands, suggesting precise GCaMP3- and ZeoR-knock-in, respectively (Fig. 2c and Fig. S6), which was confirmed by sequencing of the PCR bands (Supplementary Data 6 and 7). Furthermore, the desiccation tolerance of the modified cell line, which we named GCaMP3-KI, was not affected (p = 0.1083; Fig. 2d).

### Intracellular Ca^2+^ levels during trehalose treatment

**Figure 3:**
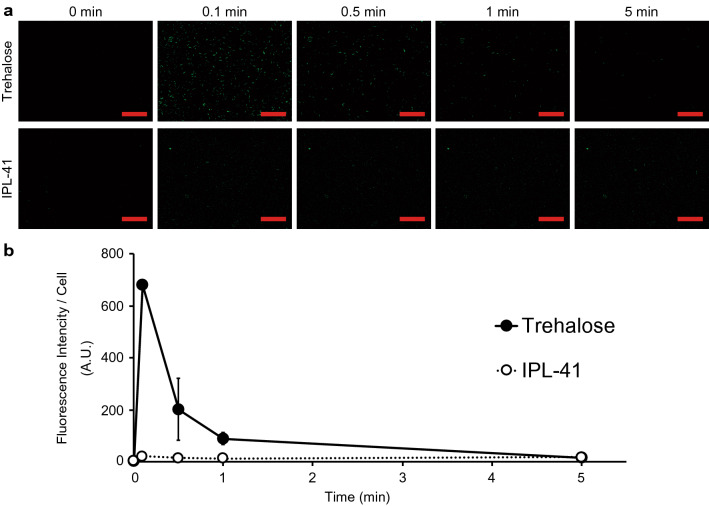
Intracellular Ca^2+^ mobilization in GCaMP3-KI cells during trehalose treatment. (**a**) Trehalose treatment induces a transient increase in GCaMP3 fluorescence in cells. (**b**) Quantitative data are shown. Scale bars, 100 µm. Values are expressed as mean ± SD; n = 3 in each group.

Using the GCaMP3-containing cell line, we examined whether the intracellular Ca^2+^ concentration changed in Pv11 cells in response to trehalose treatment, which is known to alter the expression pattern of desiccation-related genes^8^. GCaMP3-KI cells were treated with trehalose, and their fluorescence was monitored. As shown in Fig. 3a, Fig. S7 and Fig. S8, trehalose treatment induced a transient increase in GCaMP3 fluorescence in GCaMP3-KI cells (Fig. 3a, Fig. S7 and Fig. S8), while ionomycin treatment stably increased GCaMP3 fluorescence (Fig. S9). Quantitative analysis of GCaMP3 fluorescence showed a 236-fold increase at 0.1 min after trehalose treatment, after which the fluorescent intensity returned to the basal level within 5 min (Fig. 3b and Fig. S10).

### Contribution of calcium signaling pathways to desiccation tolerance

**Figure 4:**
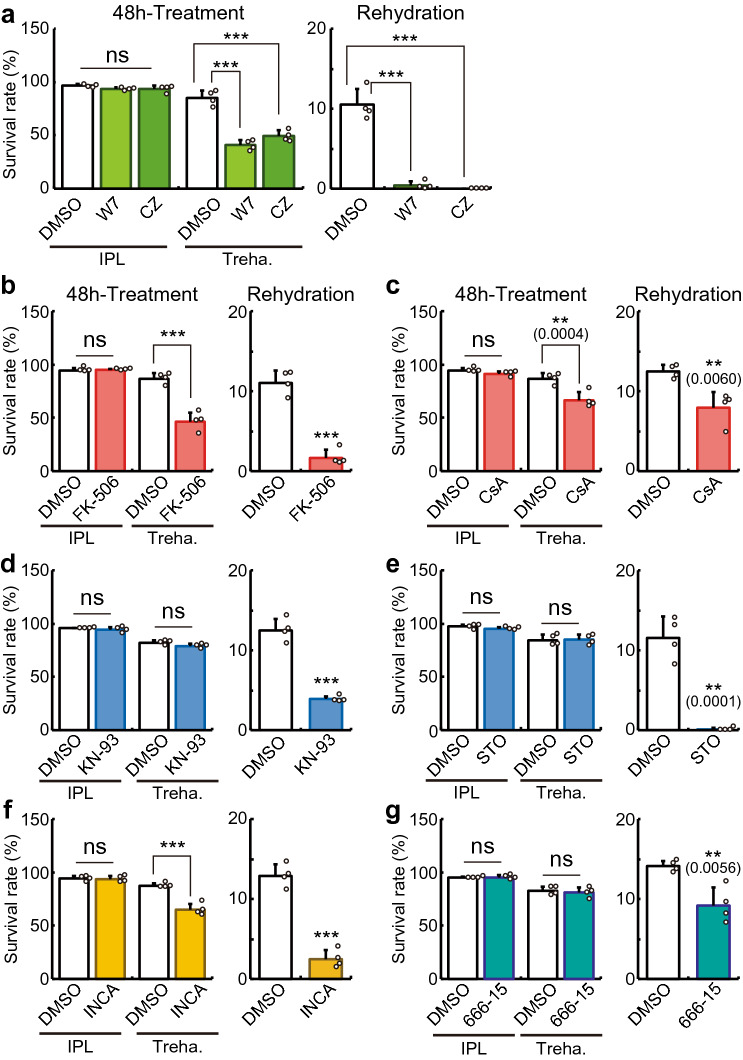
Contribution of calcium signaling pathways to desiccation tolerance in Pv11 cells. (**a**–**g**) Pv11 cells were treated with CaM inhibitors (W7 and calmidazolium; (**a**), CaN inhibitors (FK-506 and cyclosporine A; (**b**) and (**c**), respectively), CaMK inhibitors (KN-93 and STO-609; (**d**) and (**e**), respectively), an NFAT inhibitor (INCA-6; **f**) and a CREB inhibitor (666–15; **g**) and survival rates were analyzed during IPL-41/trehalose treatment or after desiccation-rehydration treatment. Values are expressed as mean ± SD; n = 4 in each group. Treatments were performed at the following concentrations: W7, 100 µM; calmidazolium, 7.5 µM; FK-506, 40 µM; cyclosporin A, 160 nM; KN-93, 30 µM; STO-609, 20 µM; INCA-6, 30 µM; and 666–15, 20 µM. ***p < 0.0001, **p < 0.01 (actual p-values are shown within parentheses).

To examine the effect of the above cytosolic calcium surge on desiccation tolerance, Pv11 cells were treated with inhibitors against the major calcium signaling pathways during the desiccation-rehydration process (Fig. S11a). First, we determined inhibitor concentrations that had no deleterious effect on the cell survival rate during growth in normal culture medium, IPL-41 (Fig. S11b,c, and Fig. 4a–g, and Supplementary Table 1). At these same concentrations, the CaM inhibitors, W7 and calmidazolium, the CaN inhibitors, FK-506 and cyclosporin A, and the NFAT inhibitor, INCA-6^50^, all decreased the survival rate after 48 h-treatment with trehalose (Fig. 4a–c,f), while the CaMK inhibitors, KN-93 and STO-609, and the CREB inhibitor, 666-15^51^, did not have such an effect (Fig. 4d,e,g). However, all inhibitors decreased the survival rate after rehydration (Fig. 4a–g). These results demonstrate the following: (i) activation of both calcium signaling pathways is probably necessary for the establishment of desiccation tolerance in Pv11 cells, (ii) the CaM–CaN–NFAT pathway confers tolerance to trehalose treatment on Pv11 cells as well as desiccation tolerance.

### Gene ontology analysis of genes potentially regulated by NFAT and CREB

**Figure 5:**
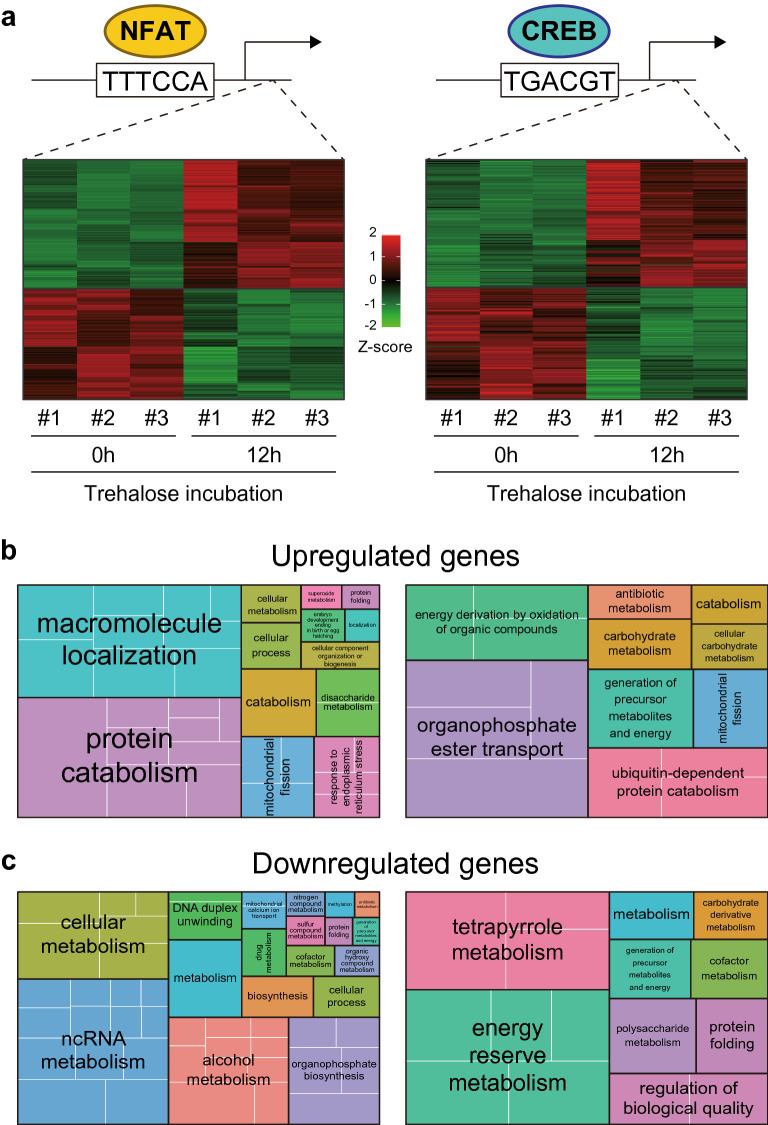
GO enrichment analysis of Pv11 genes potentially regulated by NFAT and CREB following trehalose treatment. (**a**) Heat maps of DEGs that respond to 12-h trehalose treatment, whose promoters contain NFAT or CREB consensus sequences. The heat maps show Z-scored TPM values. (**b**,**c**) Treemaps of GO enrichment analysis of upregulated and downregulated DEGs (**b** and **c**, respectively). NFAT- and CREB-related GO enrichment is shown on the left and right, respectively.

To evaluate functional differences between the two calcium signaling pathways, we performed a gene ontology (GO) analysis of differentially expressed genes (DEGs) that responded to trehalose treatment^9^ and had NFAT or CREB binding sequences in their promoter regions. Based on the fact that intracellular Ca^2+^ concentration is rapidly elevated by trehalose treatment (Fig. 3 and Fig. S8), any corresponding signal activation and gene regulation should occur during the early stages of the induction of anhydrobiosis by trehalose treatment. Therefore, we selected the shortest timepoint (12 h) from published RNA-seq data on the effect of trehalose treatment on Pv11 cells^9^ (accession number DRA008948). As shown in Fig. 5a, 1274 upregulated and 1094 downregulated DEGs in this dataset were potentially NFAT-regulated, while 124 upregulated and 109 downregulated DEGs were potentially CREB-regulated (DESeq2, FDR < 0.05; Fig. 5a and Supplementary Data 16).

GO analysis was performed on these DEGs and the data was summarized with REVIGO, based on semantic similarity (Fig. 5b,c, and Supplementary Table 3). For the upregulated DEGs, GOs relating to “protein catabolism” and “macromolecule localization” were mainly enriched in the NFAT group (Fig. 5b, the left panel). Some of the GOs were also enriched in the CREB group, such as GO:0070647 (protein modification by small protein conjugation or removal) and GO:0033036 (macromolecule localization), which were categorized in the “ubiquitin-dependent protein catabolism” and “organophosphate ester transport” clusters, respectively (Fig. 5b, the right panel and Supplementary Table 3). Furthermore, GO:0000266 (mitochondrial fission) was commonly enriched in both groups (Fig. 5b, and Supplementary Table 3). In contrast, the GO cluster “response to endoplasmic reticulum stress” was specific to the NFAT group (Fig. 5b and Supplementary Table 3). For the downregulated DEGs, metabolism-related GOs were generally enriched in both the NFAT and CREB groups (Fig. 5c and Supplementary Table 3). These data suggest that NFAT- and CREB-activation by trehalose treatment should promote the acceleration of protein catabolism and deceleration of diverse metabolic processes, which eventually invoke the ametabolic state in Pv11 cells, as observed in *P. vanderplanki* larvae^6,7^.

## Discussion

The major achievements of the current study are: (i) successful construction of genome editing tools for Pv11 cells; and (ii) the application of this technique to reveal molecular signaling pathways responsible for anhydrobiosis in Pv11 cells. The Cas9-mediated genome editing technique has been much improved recently and is now a basic tool for molecular biology even in non-model species^52–55^. Here we used the CRIS-PITCh method for inserting genes of interest (Figs. 1, 2 and Fig. S4), and in one of the KI cell lines, GCaMP3-KI, uncovered a novel function of trehalose in evoking an intracellular calcium surge; in turn, this allowed us to reveal the involvement of calcium signaling pathways in the induction of anhydrobiosis. These results suggest that trehalose plays a critical role as a signal inducer.

To improve our understanding of the molecular mechanisms underlying anhydrobiosis in Pv11 cells, we have developed several gene manipulation methods, particularly involving plasmid vectors for exogenous gene expression^4,5,56^. However, transfection of Pv11 cells can only be achieved by electroporation^4^, and in transient expression experiments, the damage incurred decreases the desiccation tolerance of the cells (Fig. S12). To avoid such temporary damage, we generated Pv11 cells that stably expressed AcGFP1 (Pv11-KH cells) after random integration of an expression vector. However, perhaps due to adventitious disruption of desiccation-related genes by genomic insertion of exogenous DNA fragments^46,47^, Pv11-KH cells show a lower desiccation tolerance than untransformed Pv11^4^ (Fig. 1d). In contrast, the method established in this study allows us to effectively express exogenous genes without deleterious effects on the desiccation tolerance of Pv11 cells (Figs. 1, 2 and Fig. S4).

The CRISPR/Cas9 system functions in Pv11 cells with either synthetic gRNAs or gRNA expression vectors (Figs. S1 and S2). Furthermore, we have shown that targeted knock-in is possible using the PITCh method (Figs. 1, 2, and Fig. S4), which allowed the establishment of stably expressing cell lines without loss of anhydrobiotic ability (Figs. 1d, 2d, and Fig. S4d). In our forthcoming study, we plan to disrupt an endogenous gene and are now constructing the donor vectors to create knock-in/knock-out alleles^11^. Thus, the current success of targeted knock-in in Pv11 cells should lead to endogenous gene knock-out experiments aimed at identifying the genes essential for anhydrobiosis (Supplementary Note).

In the current CRISPR/Cas9 technique for Pv11 cells, we acquired clonal cell lines; after transfection of a set of vectors for the CRISPR/Cas9 system, the cells were treated with zeocin to eliminate genetically unmodified or imprecisely edited cells. At the sorting process, GCaMP3-positive cells accounted only for 5.1% (Fig. S13). To increase the knock-in efficiency, optimizing homology arm length can be one of the ordinary solutions^57,58^. Generally, knock-in efficiency becomes higher as homology arms of donor DNAs become longer especially when homology arm length is short. However, knock-in efficiency also reaches a plateau at a certain length of homology arms. So, we are trying to find the best length for the current Cas9-mediated knock-in system in Pv11 cells.

GCaMP3-KI cells fluorescence strongly immediately after trehalose treatment (Fig. 3), suggesting that they (and likely therefore the parental cell type, Pv11) interpret the presence of trehalose in the extracellular medium as a specific signal. But how is this transduced into an intracellular calcium concentration spike? We hypothesize that osmotic sensors and mechanosensors, such as OSCA, Piezo and TRP cation channels^59–61^, might be responsible for the Ca^2+^ mobilization. For example, gated channels that allow mono- or divalent cations such as Na^+^ or Ca^2+^ to enter cells, have the potential to activate calcium signaling pathways. Another candidate is a gustatory receptor that senses trehalose, like Gr5a^62,63^. In Gr5a-expressing *Drosophila* S2 cells, trehalose induces an intracellular calcium surge, suggesting that the receptor functions in multiple cellular types. Furthermore, recent research raises the possibility that several gustatory receptors expressed in non-gustatory neurons have roles other than chemosensation^64,65^. To examine this possibility, in silico screening and experimental confirmation may be needed^66,67^. We believe that these approaches will clarify the molecular mechanism of trehalose sensing and calcium signaling in Pv11 cells.

Our results identify a critical role for calcium signaling pathways in anhydrobiosis in Pv11 cells (Fig. 4). A potential contribution of calcium signaling to anhydrobiosis has also been suggested in tardigrades and nematodes^68,69^. In the anhydrobiotic tardigrade, *Hypsibius dujardini*, a calmodulin inhibitor (J-8) and a calcium release modulator (2-APB) reduce the survival rate after rehydration, while inhibitors of CaN and CaMK have no effect on anhydrobiotic ability. Given that calmodulin regulates the gating of calcium ion channels in endoplasmic reticulum and mitochondria as well as the signaling pathways^70,71^, treatment with J-8 and 2-APB may disrupt calcium storage in these organelles rather than calcium signaling, leading to organelle malfunction^72,73^. RNAi-based screening of the nematode, *Panagrolaimus superbus*, showed that knockdown of “CAMK/CAMKL/MELK protein kinase” decreased the survival rate after rehydration^69^, but the contribution of the CaN–NFAT pathway or CREB to anhydrobiotic ability was not assessed. Our detailed experiments show that activation of the CaM–CaN–NFAT pathway confers tolerance both to trehalose treatment and desiccation on Pv11 cells, while activation of the CaM–CaMK–CREB pathway leads only to desiccation tolerance (Fig. 4 and Fig. S11). Therefore, our study demonstrates different functions of the individual calcium pathways in anhydrobiosis for the first time.

The GO analysis (Fig. 5b) implies one mechanism by which the CaM–CaN–NFAT pathway protects trehalose-induced Pv11 cells from cell death during anhydrobiosis (Fig. 4a–c,f). GO terms related to “response to endoplasmic reticulum stress” were enriched only in the NFAT-upregulated group (Fig. 5b and Supplementary Table 3). In general, ER stress is triggered by hyperosmolality, and it causes cell death^74–77^. Hence, in trehalose-treated Pv11 cells, the CaM–CaN–NFAT pathway could alleviate the deleterious effects of ER stress.

The GO analysis also suggests how the calcium signaling pathways might control entrance into the ametabolic state (Fig. 5). Figure 5b,c show that both NFAT and CREB can accelerate protein catabolism and decelerate diverse metabolic processes (Fig. 5b,c, and Supplementary Table 3). Furthermore, GOs related to localization, transport and mitochondria are commonly enriched in both groups (Fig. 5b,c, and Supplementary Table 3). Therefore, during trehalose treatment, Pv11 cells probably prepare existing biomolecules and organelles for forthcoming desiccation instead of activating metabolic processes.

In the current study, we succeeded in detecting Ca^2+^ mobilization using GCaMP3-KI cells. Ca^2+^ is one of many second messengers used by cells, and the knock-in technique can be used to study them since a wide variety of genetically encoded fluorescent sensors have been developed^17^. For example, cyclic AMP, inositol 1,4,5-trisphosphate, diacylglycerol and reactive oxygen species^78,79^ can all be detected by specific fluorescent sensors. In addition, there are also fluorescent sensors that report the activation status of proteases, kinases, and GTPases^80,81^. Thus, real-time monitoring of these biomolecules should uncover an anhydrobiosis-specific signaling profile. Another advantage of genetically encoded fluorescence sensors is that their subcellular localization can be controlled^80,82^. Given that differential subcellular localization of channels and enzymes contributes to their specific functions^83–89^, analyzing the active state of signal pathways with subcellular resolution should facilitate a comprehensive understanding of the anhydrobiosis-related signaling profile.

Recent reports have revealed that integrating genetic materials can change a transcriptional profile due to chromatin and chromosome conformation changes^90,91^. In the current study, GCaMP3-KI cells may have the different transcriptional profile compared to wild type cells, which might induce intracellular Ca^2+^ surge (Fig. 3). To exclude the possibility, RNA-seq data of GCaMP3-KI and wild-type cells should be compared. On the other hand, the inhibitor experiments in this article were performed in wild-type cells, so the contribution of the calcium signaling pathways in anhydrobiosis was proved independently.

In conclusion, genome editing with the CRISPR/Cas9 system has been successfully developed in Pv11 cells, and reveals the critical contribution of calcium signaling pathways to anhydrobiosis. This technique will allow further advanced molecular engineering strategies, such as gene knockout, and enable us to characterize the mechanisms underlying anhydrobiosis in Pv11 cells.

## Methods

### Cell culture

Pv11 and Pv11-KH cells were grown in IPL-41 medium (Thermo Fisher Scientific, Waltham, MA) supplemented with 2.6 g/L tryptose phosphate broth (Becton, Dickinson and Company, Franklin Lakes, NJ), 10% (v/v) fetal bovine serum, and 0.05% (v/v) of an antibiotic and antimycotic mixture (penicillin, amphotericin B, and streptomycin; MilliporeSigma, Burlington, MA)^5^, designated hereafter as complete IPL-41 medium.

### Expression vectors

The SpCas9-expressing vector was constructed by replacing the AcGFP1 region of pPv121-AcGFP1 using a HiFi Assembly kit (New England BioLabs, Ipswich, MA)^56^. The SpCas9 gene was cloned from pBS-Hsp70-Cas9, which was a gift from Melissa Harrison, Kate O'Connor-Giles and Jill Wildonger (Addgene plasmid # 46294; http://n2t.net/addgene:46294; RRID: Addgene_46294).

The PvU6b promoter was cloned after reference to the gene model in MidgeBase (http://bertone.nises-f.affrc.go.jp/midgebase/). The pPvU6b plasmid was constructed by replacing the DmU6 promoter of pU6-BbsI-chiRNA with the PvU6b promoter and inserting the DmtRNA sequence described previously^44,45^. pU6-BbsI-chiRNA was a gift from Melissa Harrison, Kate O'Connor-Giles and Jill Wildonger (Addgene plasmid # 45946; http://n2t.net/addgene:45946; RRID:Addgene_45946)^92^, and we named the vector pPvU6b-DmtRNA-BbsI (the complete sequence is shown in Supplementary Data 1). To express the gRNA targeting the 5′-flanking site of the stop codon of *Pv.00443* (Fig. 1), pPvU6b-DmtRNA-Pv.00443#1 was constructed as follows: (i) pPvU6b-DmtRNA-BbsI was restricted with BbsI, (ii) two oligonucleotides were annealed (Supplementary Table 2), and (iii) the annealed DNA was ligated into the cut vector (the complete sequences of the gRNA-expression vectors used in the current study are shown in Supplementary Data 8, 9 and 10).

### Donor vectors

The donor vector containing the AcGFP1-P2A-ZeoR sequence (Fig. 1) was constructed using PCR, a HiFi Assembly kit (New England BioLabs) and a TOPO cloning kit (Thermo Fisher Scientific). Briefly, the polycistronic sequence of AcGFP1-P2A-ZeoR was inserted into the pPv121 vector using a HiFi Assembly kit (New England BioLabs). Then, the vector was used as a PCR template to add another P2A sequence to the 5′ end of AcGFP1, and the PCR product was inserted into the pCR-Blunt II-TOPO vector (Thermo Fisher Scientific). Next, the vector was used as a PCR template to add the gRNA-target and microhomology sequences, and the PCR product was also cloned into pCR-Blunt II-TOPO (the complete sequence is shown in Supplementary Data 2). To construct the donor vector for inserting the HaloTag gene (Fig. S4), pCR4 Blunt-TOPO was used for cloning. To construct the donor vector for inserting the other genes, we created the basic vector, pCR4-Pv.00443#1µH-P2A-BbsI, which contains the gRNA-target, microhomology, P2A, and BbsI sequences; first, a synthetic gene with the sequences was acquired for the PCR template (eurofins Genomics, Tokyo, Japan), and the PCR product was cloned into pCR4 Blunt-TOPO (the complete sequence is shown in Supplementary Data 11). To construct the donor vectors for inserting the BlaR, ZeoR, and GCaMP3 genes (Fig. S4 and Fig. 2), the basic vector was restricted with BbsI (New England BioLabs) and HiFi Assembly was performed. The HaloTag, BlaR and GCaMP3 genes were cloned from pFN19K HaloTag T7 SP6 (Promega, Fitchburg, WI), pYES6/CT (Thermo Fisher Scientific) and G-CaMP3 (a gift from Loren Looger; Addgene plasmid # 22692; http://n2t.net/addgene:22692; RRID:Addgene_22692)^93^, respectively (the complete sequences are shown in Supplementary Data 12, 13, 14 and 15).

### Transfection, drug selection and cell sorting

The cells used in each experiment were seeded at a density of 3 × 10^5^ cells per mL into a 25 cm^2^ cell culture flask and grown at 25 °C for 4–6 days before transfection. Transfection was carried out using a NEPA21 Super Electroporator (Nepa Gene, Chiba, Japan) as described previously^4^. Five µg each of the gRNA- and SpCas9-expression vectors plus 0.03–0.1 pmol donor vectors were transfected into the cells. Five days after transfection, the cells at a density of 1 × 10^5^ cells per mL were treated with 400 µg/mL zeocin or 200 µg/mL blasticidin. After 1 week of drug selection, the medium was changed to normal IPL-41 medium and the cells were incubated for a further 2 weeks. To establish clonal cell lines, single cell sorting was performed using a MoFlo Astrios cell-sorter (Beckman Coulter, Brea, CA) equipped with 355-, 488- and 640-nm lasers. One thousand intact Pv11 cells were seeded as a feeder layer in each well of a 96-well plate prior to sorting. HaloTag-positive cells were labeled with fluorescent Janelia Fluor 646 HaloTag Ligand (Promega). GCaMP3-KI cells exhibited strong fluorescence when passed through a flow cytometer, perhaps due to shear or/and mechanical stress (Fig. S5). The cells were stained with DAPI (Dojindo, Kumamoto, Japan), and DAPI, GCaMP3 and Janelia Fluor 646 HaloTag Ligand were excited with 355-nm, 488-nm and 640-nm lasers, respectively. After single cell sorting, the cells were grown for 2 weeks, and then treated with zeocin or blasticidin to eliminate feeder cells. If the drug selection was not completely effective, bulk cell sorting was performed.

### Genomic PCR and sequencing analysis

To confirm precise gene knock-in, genomic PCR and sequencing analysis were performed (Figs. 1, 2, Fig. S4). For genomic PCR, the genomes of Pv11 cells and clonal cell lines were extracted with a NucleoSpin Tissue kit (Takara Bio, Shiga, Japan) and subjected to PCR using the following primer set: 5′-GCCAAAGCGAGCCAATTCAA-3′ and 5′-GGGTGTTATTGCTACTTTAATGCGT-3′. PCR images were acquired using a ChemiDoc Touch imaging system (Bio-Rad, Hercules, CA). After gel purification of the PCR products, sub-cloning was carried out using a TOPO cloning kit (Thermo Fisher Scientific) and plasmids were sequenced.

### Desiccation and rehydration

Pv11 cells were subjected to desiccation-rehydration (Figs. 1d, 2d, 4, Fig. S4d) as described previously^3^. Briefly, cells were incubated in preconditioning medium (600 mM trehalose containing 10% (v/v) complete IPL-41 medium) for 48 h at 25 °C, and then suspended in 400 µL preconditioning medium. Forty microliter aliquots of the cell suspension were dropped into 35-mm petri dishes, and the dishes were desiccated and maintained at < 10% relative humidity and 25 °C for more than 10 days. An hour after rehydration by complete IPL-41 medium, cells were stained with propidium iodide (PI; Dojindo) for dead-cell detection and Hoechst 33342 (Dojindo) for whole-cell detection, and images were acquired using a conventional fluorescence microscope (BZ-X700; Keyence, Osaka, Japan). The survival rate was calculated as the ratio of the number of live cells (Hoechst-positive and PI-negative) to that of total cells (Hoechst-positive).

### GCaMP3 fluorescence quantification

Images for GCaMP3 fluorescence quantification of the cells were acquired as follows: (i) 50 µL of complete IPL-41 media with 10 µg/mL Hoechst 33342 (Dojindo) was placed in a well of a 48-well plate, (ii) 50 µL of cell suspension was added to the well, and cells were allowed to attach to the culture surface (usually 7–10 min), (iii) a “0 min” image was acquired, (iv) 900 µL of 600 mM trehalose, complete IPL-41 or complete IPL-41 with ionomycin was added into the well, and time course images were acquired. The brightness of GCaMP3 fluorescence was quantified using Hybrid Cell Count BZ-H2C software (Keyence). Cell numbers were also counted using Hoechst 33342 fluorescence and the same software.

### Ionomycin or inhibitor treatment

GCaMP3-KI cells were treated with ionomycin at 10 µM (Fig. 2b, Figs. S9 and S10, and Supplementary Table 1). To inhibit calcium signaling due to trehalose treatment, Pv11 cells were pretreated with inhibitors for 1 h, and then treated with trehalose or complete IPL-41 plus inhibitors (Figs. 4 and S11b, and Supplementary Table 1).

### mRNA-seq data processing

RNA-Seq data of Pv11 cells exposed to 600 mM trehalose were obtained from our previous study^9^ (accession number DRA008948). Reads were mapped to Pv_5.2 CDS sequences (https://midgebase2.dna.affrc.go.jp/) and expression values were quantified with bowtie2 v2.4.2^94^ and RSEM v1.2.30^95^ using the Trinity v2.9.1^96,97^ utility. Mapped read counts were tested for differential expression with DESeq2 v1.26.0^98^. Transcripts with FDR below 0.05 were determined as differentially expressed. Z-scaled TPM values were visualized as a heat map, by clustering 1-Spearman correlation with the Ward’s method in R.

### Motif enrichment analysis

To identify genes with NFAT or CREB binding motifs in the promoter region, we utilized HOMER2 v4.10.0^98^. Binding motifs were obtained from the HOMER2 motif database [NFAT(RHD), CRE(bZIP)] and the − 2000 bp to + 150 bp genomic regions surrounding transcription start sites were searched with the annotatePeaks.pl utility in HOMER2. Transcripts with either motif were analyzed with GOstat v2.52.0^99^ to identify enriched gene ontologies using the Pv_5.2 annotation. Enriched ontologies were summarized and visualized with REVIGO^100^, accessed February 2021, using tiny settings.

### Statistics and reproducibility

All data were expressed as mean ± SD. Differences between two groups were examined for statistical significance by the two-sided Student *t*-test in Figs. 2d, 4 and Fig. S4d. Differences among more than three groups were examined by ANOVA followed by a Tukey test (Figs. 1d and 4). A *P*-value < 0.05 denoted a statistically significant difference. GraphPad Prism 8 software (GraphPad, San Diego, CA) was used for statistical analyses. Experiments were independently repeated at least three times in general. The raw data underlying plots in the figures are available in Supplementary Data 16.

## Supplementary Information

Supplementary Information 1.

Supplementary Information 2.

Supplementary Information 3.

Supplementary Information 4.

Supplementary Information 5.

Supplementary Information 6.

Supplementary Information 7.

## Data Availability

The datasets shown in the current study are available from the corresponding author on reasonable request. The source data of Figs. 1d, 2d, 3b, 4a–g and 5a, and Supplementary Figures S1b, S2b, S4d, S5b,c, S10, S11c, and S12 are provided in Supplementary Data 16.
