# Supplementary material for: Cas9-mediated genome editing reveals a significant contribution of calcium signaling pathways to anhydrobiosis in Pv11 cells
Source: Sci Rep. 2021 Oct 5;11:19698. doi: 10.1038/s41598-021-98905-w (PMC8492635; doi:10.1038/s41598-021-98905-w)
Supplement: Supplementary file 1 — Supplementary Information 1. [file 41598_2021_98905_MOESM1_ESM.pdf]

Supplementary Data 1. The full-length sequence of pPvU6b-DmtRNA-BbsI plasmid.

```
LOCUS       pPvU6b-DmtRNA-BbsI_sequence      3097 bp    DNA     circular UNA
FEATURES             Location/Qualifiers
     promoter        62..747
                     /label="PvU6b promoter"
     tRNA            748..844
                     /label="Dm tRNA_gly"
     Site            complement(847..852)
                     /site_type="restriction site"
                     /label="BbsI"
     Site            855..860
                     /site_type="restriction site"
                     /label="BbsI"
     misc_feature    863..938
                     /feature_type="Zinc finger"
                     /label="gRNA core"
     terminator      939..944
                     /label="pol III terminator"
     promoter        complement(1064..1185)
                     /label="lac promoter"
     primer          complement(1108..1137)
                     /label="lac promoter-R"
     rep_origin      1405..2024
                     /label="pUC ori"
     promoter        2645..2722
                     /label="EM7 promoter"
     CDS             2723..3097
                     /label="bleo"
                     /gene="bleo"
                     /translation="MRKEKIPHQEIVSVNNSEELVKKAIEGDALRIGSGDTVKHEEAV
                     SPFAAKLFSNITGSQRYVLIAVRHTQPATVDESRKAAIFHHDIRQAGIAMGHDEILAV
                     GHARLEPGEQFGWREPLMLFVQIILIDKTGFHPSTCSLDAMFRLVVEWAGSRIKRMQP
                     PHCISHDGYFLGRSKVR*"
                     /label="Zeo+/Bleo+"
ORIGIN
        1 CCGACGCCGA CCAACACCGC CGGTCCGACG CGGCCCGACG GGTCCGAGGG GGGTCGACCT
       61 CAAAATTCTA AAGCTGACTT TTTAAATTTT TTACTTTTCT TTAATTCTTT CAATTAAAAT
      121 TTAAATAAAA AATTCAAACA TTTTTTCTCT TCTTTTTATA TTTAGTTTCA GTTCATTTTG
      181 TACTCAAATA TTGACATAAT AAAATAAATT TCATATTAAT TAATCGAAAT ATTTGTGTCA
      241 TAAATAAATA ACTAAGAAAA AAAAGGGTTT GTCATGTCGT GTGTCGAATG TCCAAGTGAA
      301 AATGTTTGTT TAACAAATTG AAATTTCATT TAATTAACAC CGCATTGTGC ATAAGAGAAA
      361 GGAAAGAAAG AAAAGAAAAT TAGCTTGGTC TGATTACAAA TAAAAAAGGG TTCAGGTAAT
      421 AAAAAAAATT CCTTTCACTC GTTCATCATC AAAATCACTT CTGGTTTACA AGTTGAGGTC
      481 AAATGAAGTC AAATGAATAT CAAAAAATAA ATTGCAGTTG ATTGACTGTT TATAGCAGCT
      541 CATATATGAT CAGTAGACGA TTATCGATAA ATTTTTATTA AGCACGAGCT GTTTGAGGTC
      601 AATTTCAGTT GATTGCAGTG CAAAACCAGC TGCAAAATTG TTTTCATATT TCACTTGTTC
      661 CAAATGTTTT GTTTTGTTTC ATTTTCCATT TCGTTTCATT TCAATAGCAG CAGTGGTTTA
      721 AGTAGCATTT CTGCCAAATA CAAAATTGGG CTTTGAGTGT GTGTAGACAT CAAGCATCGG
      781 TGGTTCAGTG GTAGAATGCT CGCCTGCCAC GCGGGCGGCC CGGGTTCGAT TCCCGGCCGA
      841 TGCAGGGTCT TCGAGAAGAC CTGTTTTAGA GCTAGAAATA GCAAGTTAAA ATAAGGCTAG
      901 TCCGTTATCA ACTTGAAAAA GTGGCACCGA GTCGGTGCTT TTTTatcgaa ttcctgcagc
      961 ccggGGGATC CACTAGTTCT AGAGCGGCCG CCACCGCGGT GGAGCTCCAG CTTTTGTTCC
     1021 CTTTAGTGAG GGTTAATTTC GAGCTTGGCG TAATCATGGT CATAGCTGTT TCCTGTGTGA
     1081 AATTGTTATC CGCTCACAAT TCCACACAAC ATACGAGCCG GAAGCATAAA GTGTAAAGCC
     1141 TGGGGTGCCT AATGAGTGAG CTAACTCACA TTAATTGCGT TGCGCTCACT GCCCGCTTTC
     1201 CAGTCGGGAA ACCTGTCGTG CCAGCTGCAT TAATGAATCG GCCAACGCGC GGGGAGAGGC
     1261 GGTTTGCGTA TTGGGCGCTC TTCCGCTTCC TCGCTCACTG ACTCGCTGCG CTCGGTCGTT
     1321 CGGCTGCGGC GAGCGGTATC AGCTCACTCA AAGGCGGTAA TACGGTTATC CACAGAATCA
     1381 GGGGATAACG CAGGAAAGAA CATGCGCGTT GCTGGCGTTT TTCCATAGGC TCCGCCCCCC
     1441 TGACGAGCAT CACAAAAATC GACGCTCAAG TCAGAGGTGG CGAAACCCGA CAGGACTATA
     1501 AAGATACCAG GCGTTTCCCC CTGGAAGCTC CCTCGTGCGC TCTCCTGTTC CGACCCTGCC
     1561 GCTTACCGGA TACCTGTCCG CCTTTCTCCC TTCGGGAAGC GTGGCGCTTT CTCATAGCTC
     1621 ACGCTGTAGG TATCTCAGTT CGGTGTAGGT CGTTCGCTCC AAGCTGGGCT GTGTGCACGA
     1681 ACCCCCCGTT CAGCCCGACC GCTGCGCCTT ATCCGGTAAC TATCGTCTTG AGTCCAACCC
     1741 GGTAAGACAC GACTTATCGC CACTGGCAGC AGCCACTGGT AACAGGATTA GCAGAGCGAG
     1801 GTATGTAGGC GGTGCTACAG AGTTCTTGAA GTGGTGGCCT AACTACGGCT ACACTAGAAG
     1861 AACAGTATTT GGTATCTGCG CTCTGCTGAA GCCAGTTACC TTCGGAAAAA GAGTTGGTAG
     1921 CTCTTGATCC GGCAAACAAA CCACCGCTGG TAGCGGTGGT TTTTTTGTTT GCAAGCAGCA
     1981 GATTACGCGC AGAAAAAAAG GATCTCAAGA AGATCCTTTG ATCTTTTCTA CGGGGTCTGA
     2041 CGCTCAGTGG AACGAAAACT CACGTTAAGG GATTTTGGTC ATGATGATAA ACAATGTATG
     2101 GTGCTAATGT TGCTTCAACA ACAATTCTGT TGAACTGTGT TTTCATGTTT GCCAACAAGC
     2161 ACCTTTATAC TCGGTGGCCT CCCCACCACC AACTTTTTTG CACTGCAAAA AAACACGCTT
     2221 TTGCACGCGG GCCCATACAT AGTACAAACT CTACGTTTCG TAGACTATTT TACATAAATA
     2281 GTCTACACCG TTGTATACGC TCCAAATACA CTACCACACA TTGAACCTTT TTGCAGTGCA
     2341 AAAAAGTACG TGTCGGCAGT CACGTAGGCC GGCCTTATCG GGTCGCGTCC TGTCACGTAC
     2401 GAATCACATT ATCGGACCGG ACGAGTGTTG TCTTATCGTG ACAGGACGCC AGCTTCCTGT
     2461 GTTGCTAACC GCAGCCGGAC GCAACTCCTT ATCGGAACAG GACGCGCCTC CATATCAGCC
     2521 GCGCGTTATC TCATGCGCGT GACCGGACAC GAGGCGCCCG TCCCGCTTAT CGCGCCTATA
     2581 AATACAGCCC GCAACGATCT GGTAAACACA GTTGAACAGC ATCTGTTCGA ATTAATTCGG
     2641 ATCTCTGCAG CACGTGTTGA CAATTAATCA TCGGCATAGT ATATCGGCAT AGTATAATAC
     2701 GACTCACTAT AGGAGGGCCA CCATGGCCAA GTTGACCAGT GCCGTTCCGG TGCTCACCGC
     2761 GCGCGACGTC GCCGGAGCGG TCGAGTTCTG GACCGACCGG CTCGGGTTCT CCCGGGACTT
     2821 CGTGGAGGAC GACTTCGCCG GTGTGGTCCG GGACGACGTG ACCCTGTTCA TCAGCGCGGT
     2881 CCAGGACCAG GTGGTGCCGG ACAACACCCT GGCCTGGGTG TGGGTGCGCG GCCTGGACGA
     2941 GCTGTACGCC GAGTGGTCGG AGGTCGTGTC CACGAACTTC CGGGACGCCT CCGGGCCGGC
     3001 CATGACCGAG ATCGGCGAGC AGCCGTGGGG GCGGGAGTTC GCCCTGCGCG ACCCGGCCGG
     3061 CAACTGCGTG CACTTCGTGG CCGAGGAGCA GGACTGA
//
```

Supplementary Data 2. The full-length sequence of the donor vector, pCRII-Pv.00443#1ｷH-P2A-AcGFP1-P2A-ZeoR.

```
LOCUS       pCRII-Pv.00443#1-P2A-AcGFP1-P2A-ZeoR_sequence      4869 bp    DNA     circular UNA 21-11-2017
DEFINITION
FEATURES             Location/Qualifiers
     promoter        143..172
                     /label="lac_promoter"
     primer          186..208
                     /label="M13_pUC_rev_primer"
     primer          207..225
                     /label="M13_reverse_primer"
     primer          239..256
                     /label="Sp6_primer"
     misc_feature    337..359
                     /feature_type="Zinc finger"
                     /label="Pv.00443-gRNA#1"
     misc_feature    337..420
                     /feature_type="Primer Region"
                     /PCR_conditions="Tm=63.6"
                     /label="Pv.00443#1-Asn-P2A-PITCh_F"
     microhomology   360..399
                     /label="Left microhomology of Pv.00443"
     Site            403..468
                     /site_type="restriction site"
                     /label="Linker + DmP2A"
     CDS             469..1185
                     /cds_type="ORF"
                     /label="AcGFP1 (stop codon less)"
     Site            1186..1251
                     /site_type="restriction site"
                     /label="Linker + DmP2A"
     CDS             1252..1623
                     /cds_type="ORF"
                     /label="Zeo+/Bleo+ (Stop codon less)"
     microhomology   1624..1663
                     /label="Right microhomology of Pv.00443"
     misc_feature    complement(1664..1686)
                     /feature_type="Zinc finger"
                     /label="Pv.00443-gRNA#1"
     primer          complement(1757..1775)
                     /label="T7_promoter"
     primer          complement(1782..1798)
                     /label="M13_forward20_primer"
     primer          complement(1791..1813)
                     /label="M13_pUC_fwd_primer"
     CDS             2590..3378
                     /cds_type="ORF"
                     /label="NeoR/KanR"
     CDS             3588..3959
                     /cds_type="ORF"
                     /label="Bleo"
     rep_origin      4084..4703
                     /label="pBR322_origin"
ORIGIN
        1 AGCGCCCAAT ACGCAAACCG CCTCTCCCCG CGCGTTGGCC GATTCATTAA TGCAGCTGGC
       61 ACGACAGGTT TCCCGACTGG AAAGCGGGCA GTGAGCGCAA CGCAATTAAT GTGAGTTAGC
      121 TCACTCATTA GGCACCCCAG GCTTTACACT TTATGCTTCC GGCTCGTATG TTGTGTGGAA
      181 TTGTGAGCGG ATAACAATTT CACACAGGAA ACAGCTATGA CCATGATTAC GCCAAGCTAT
      241 TTAGGTGACA CTATAGAATA CTCAAGCTAT GCATCAAGCT TGGTACCGAG CTCGGATCCA
      301 CTAGTAACGG CCGCCAGTGT GCTGGAATTC GCCCTTTTAT AAGATTTTAA TTATGATGGT
      361 CATCATCATA GAGTAGGCAG AAAACATCAT TGCCATCATA ATGGTTCCGG TGCCACAAAC
      421 TTCAGTTTGT TAAAGCAGGC CGGAGACGTC GAAGAGAACC CCGGACCGAT GGTGAGCAAG
      481 GGCGCCGAGC TGTTCACCGG CATCGTGCCC ATCCTGATCG AGCTGAATGG CGATGTGAAT
      541 GGCCACAAGT TCAGCGTGAG CGGCGAGGGC GAGGGCGATG CCACCTACGG CAAGCTGACC
      601 CTGAAGTTCA TCTGCACCAC CGGCAAGCTG CCTGTGCCCT GGCCCACCCT GGTGACCACC
      661 CTGAGCTACG GCGTGCAGTG CTTCTCACGC TACCCCGATC ACATGAAGCA GCACGACTTC
      721 TTCAAGAGCG CCATGCCTGA GGGCTACATC CAGGAGCGCA CCATCTTCTT CGAGGATGAC
      781 GGCAACTACA AGTCGCGCGC CGAGGTGAAG TTCGAGGGCG ATACCCTGGT GAATCGCATC
      841 GAGCTGACCG GCACCGATTT CAAGGAGGAT GGCAACATCC TGGGCAATAA GATGGAGTAC
      901 AACTACAACG CCCACAATGT GTACATCATG ACCGACAAGG CCAAGAATGG CATCAAGGTG
      961 AACTTCAAGA TCCGCCACAA CATCGAGGAT GGCAGCGTGC AGCTGGCCGA CCACTACCAG
     1021 CAGAATACCC CCATCGGCGA TGGCCCTGTG CTGCTGCCCG ATAACCACTA CCTGTCCACC
     1081 CAGAGCGCCC TGTCCAAGGA CCCCAACGAG AAGCGCGATC ACATGATCTA CTTCGGCTTC
     1141 GTGACCGCCG CCGCCATCAC CCACGGCATG GATGAGCTGT ACAAGGGTTC CGGTGCCACA
     1201 AACTTCAGTT TGTTAAAGCA GGCCGGAGAC GTCGAAGAGA ACCCCGGACC GATGGCCAAG
     1261 TTGACCAGTG CCGTTCCGGT GCTCACCGCG CGCGACGTCG CCGGAGCGGT CGAGTTCTGG
     1321 ACCGACCGGC TCGGGTTCTC CCGGGACTTC GTGGAGGACG ACTTCGCCGG TGTGGTCCGG
     1381 GACGACGTGA CCCTGTTCAT CAGCGCGGTC CAGGACCAGG TGGTGCCGGA CAACACCCTG
     1441 GCCTGGGTGT GGGTGCGCGG CCTGGACGAG CTGTACGCCG AGTGGTCGGA GGTCGTGTCC
     1501 ACGAACTTCC GGGACGCCTC CGGGCCGGCC ATGACCGAGA TCGGCGAGCA GCCGTGGGGG
     1561 CGGGAGTTCG CCCTGCGCGA CCCGGCCGGC AACTGCGTGC ACTTCGTGGC CGAGGAGCAG
     1621 GACTAAAATC TTATAACAAC ATCCTAAAGT ATTATAACAC CAACCATCAT AATTAAAATC
     1681 TTATAAAAGG GCGAATTCTG CAGATATCCA TCACACTGGC GGCCGCTCGA GCATGCATCT
     1741 AGAGGGCCCA ATTCGCCCTA TAGTGAGTCG TATTACAATT CACTGGCCGT CGTTTTACAA
     1801 CGTCGTGACT GGGAAAACCC TGGCGTTACC CAACTTAATC GCCTTGCAGC ACATCCCCCT
     1861 TTCGCCAGCT GGCGTAATAG CGAAGAGGCC CGCACCGATC GCCCTTCCCA ACAGTTGCGC
     1921 AGCCTATACG TACGGCAGTT TAAGGTTTAC ACCTATAAAA GAGAGAGCCG TTATCGTCTG
     1981 TTTGTGGATG TACAGAGTGA TATTATTGAC ACGCCGGGGC GACGGATGGT GATCCCCCTG
     2041 GCCAGTGCAC GTCTGCTGTC AGATAAAGTC TCCCGTGAAC TTTACCCGGT GGTGCATATC
     2101 GGGGATGAAA GCTGGCGCAT GATGACCACC GATATGGCCA GTGTGCCGGT CTCCGTTATC
     2161 GGGGAAGAAG TGGCTGATCT CAGCCACCGC GAAAATGACA TCAAAAACGC CATTAACCTG
     2221 ATGTTCTGGG GAATATAAAT GTCAGGCATG AGATTATCAA AAAGGATCTT CACCTAGATC
     2281 CTTTTCACGT AGAAAGCCAG TCCGCAGAAA CGGTGCTGAC CCCGGATGAA TGTCAGCTAC
     2341 TGGGCTATCT GGACAAGGGA AAACGCAAGC GCAAAGAGAA AGCAGGTAGC TTGCAGTGGG
     2401 CTTACATGGC GATAGCTAGA CTGGGCGGTT TTATGGACAG CAAGCGAACC GGAATTGCCA
     2461 GCTGGGGCGC CCTCTGGTAA GGTTGGGAAG CCCTGCAAAG TAAACTGGAT GGCTTTCTCG
     2521 CCGCCAAGGA TCTGATGGCG CAGGGGATCA AGCTCTGATC AAGAGACAGG ATGAGGATCG
     2581 TTTCGCATGA TTGAACAAGA TGGATTGCAC GCAGGTTCTC CGGCCGCTTG GGTGGAGAGG
     2641 CTATTCGGCT ATGACTGGGC ACAACAGACA ATCGGCTGCT CTGATGCCGC CGTGTTCCGG
     2701 CTGTCAGCGC AGGGGCGCCC GGTTCTTTTT GTCAAGACCG ACCTGTCCGG TGCCCTGAAT
     2761 GAACTGCAAG ACGAGGCAGC GCGGCTATCG TGGCTGGCCA CGACGGGCGT TCCTTGCGCA
     2821 GCTGTGCTCG ACGTTGTCAC TGAAGCGGGA AGGGACTGGC TGCTATTGGG CGAAGTGCCG
     2881 GGGCAGGATC TCCTGTCATC TCACCTTGCT CCTGCCGAGA AAGTATCCAT CATGGCTGAT
     2941 GCAATGCGGC GGCTGCATAC GCTTGATCCG GCTACCTGCC CATTCGACCA CCAAGCGAAA
     3001 CATCGCATCG AGCGAGCACG TACTCGGATG GAAGCCGGTC TTGTCGATCA GGATGATCTG
     3061 GACGAAGAGC ATCAGGGGCT CGCGCCAGCC GAACTGTTCG CCAGGCTCAA GGCGAGCATG
     3121 CCCGACGGCG AGGATCTCGT CGTGACCCAT GGCGATGCCT GCTTGCCGAA TATCATGGTG
     3181 GAAAATGGCC GCTTTTCTGG ATTCATCGAC TGTGGCCGGC TGGGTGTGGC GGACCGCTAT
     3241 CAGGACATAG CGTTGGCTAC CCGTGATATT GCTGAAGAGC TTGGCGGCGA ATGGGCTGAC
     3301 CGCTTCCTCG TGCTTTACGG TATCGCCGCT CCCGATTCGC AGCGCATCGC CTTCTATCGC
     3361 CTTCTTGACG AGTTCTTCTG AATTATTAAC GCTTACAATT TCCTGATGCG GTATTTTCTC
     3421 CTTACGCATC TGTGCGGTAT TTCACACCGC ATACAGGTGG CACTTTTCGG GGAAATGTGC
     3481 GCGGAACCCC TATTTGTTTA TTTTTCTAAA TACATTCAAA TATGTATCCG CTCATGAGAC
     3541 AATAACCCTG ATAAATGCTT CAATAATAGC ACGTGAGGAG GGCCACCATG GCCAAGTTGA
     3601 CCAGTGCCGT TCCGGTGCTC ACCGCGCGCG ACGTCGCCGG AGCGGTCGAG TTCTGGACCG
     3661 ACCGGCTCGG GTTCTCCCGG GACTTCGTGG AGGACGACTT CGCCGGTGTG GTCCGGGACG
     3721 ACGTGACCCT GTTCATCAGC GCGGTCCAGG ACCAGGTGGT GCCGGACAAC ACCCTGGCCT
     3781 GGGTGTGGGT GCGCGGCCTG GACGAGCTGT ACGCCGAGTG GTCGGAGGTC GTGTCCACGA
     3841 ACTTCCGGGA CGCCTCCGGG CCGGCCATGA CCGAGATCGG CGAGCAGCCG TGGGGGCGGG
     3901 AGTTCGCCCT GCGCGACCCG GCCGGCAACT GCGTGCACTT CGTGGCCGAG GAGCAGGACT
     3961 GACACGTGCT AAAACTTCAT TTTTAATTTA AAAGGATCTA GGTGAAGATC CTTTTTGATA
```

```
     4021 ATCTCATGAC CAAAATCCCT TAACGTGAGT TTTCGTTCCA CTGAGCGTCA GACCCCGTAG
     4081 AAAAGATCAA AGGATCTTCT TGAGATCCTT TTTTTCTGCG CGTAATCTGC TGCTTGCAAA
     4141 CAAAAAAACC ACCGCTACCA GCGGTGGTTT GTTTGCCGGA TCAAGAGCTA CCAACTCTTT
     4201 TTCCGAAGGT AACTGGCTTC AGCAGAGCGC AGATACCAAA TACTGTCCTT CTAGTGTAGC
     4261 CGTAGTTAGG CCACCACTTC AAGAACTCTG TAGCACCGCC TACATACCTC GCTCTGCTAA
     4321 TCCTGTTACC AGTGGCTGCT GCCAGTGGCG ATAAGTCGTG TCTTACCGGG TTGGACTCAA
     4381 GACGATAGTT ACCGGATAAG GCGCAGCGGT CGGGCTGAAC GGGGGGTTCG TGCACACAGC
     4441 CCAGCTTGGA GCGAACGACC TACACCGAAC TGAGATACCT ACAGCGTGAG CTATGAGAAA
     4501 GCGCCACGCT TCCCGAAGGG AGAAAGGCGG ACAGGTATCC GGTAAGCGGC AGGGTCGGAA
     4561 CAGGAGAGCG CACGAGGGAG CTTCCAGGGG GAAACGCCTG GTATCTTTAT AGTCCTGTCG
     4621 GGTTTCGCCA CCTCTGACTT GAGCGTCGAT TTTTGTGATG CTCGTCAGGG GGGCGGAGCC
     4681 TATGGAAAAA CGCCAGCAAC GCGGCCTTTT TACGGTTCCT GGGCTTTTGC TGGCCTTTTG
     4741 CTCACATGTT CTTTCCTGCG TTATCCCCTG ATTCTGTGGA TAACCGTATT ACCGCCTTTG
     4801 AGTGAGCTGA TACCGCTCGC CGCAGCCGAA CGACCGAGCG CAGCGAGTCA GTGAGCGAGG
     4861 AAGCGGAAG
//
```

Supplementary Data 3. The genome sequence of AcGFP1-KI cell.

```
LOCUS       genome_sequence_of_AcGFP1-KI      1840 bp    DNA     linear   UNA 06-2-2018
DEFINITION
FEATURES             Location/Qualifiers
     primer_bind     1..20
                     /PCR_conditions="60.04 'C"
                     /label="YM_Pv.00443-F1"
     CDS             1..332
                     /cds_type="ORF"
                     /label="Pv.00443"
     microhomology   290..329
                     /label="Left homology"
     Site            333..398
                     /site_type="restriction site"
                     /label="P2A"
     CDS             399..1115
                     /cds_type="ORF"
                     /label="AcGFP1"
     Site            1116..1181
                     /site_type="restriction site"
                     /label="P2A"
     CDS             1182..1553
                     /cds_type="ORF"
                     /label="Zeocin"
     microhomology   1554..1593
                     /label="Right microhomology"
     primer          complement(1816..1840)
                     /PCR_conditions="60.16 'C"
                     /label="YM_Pv.00443-R1"
ORIGIN
        1 GCCAAAGCGA GCCAATTCAA CCACCTCGCT ATTCCTTCGC TGAGCGACAA GAATCATCTA
       61 GTCGAGATCA TGGAGAAGGT CCATCACACG ATTGTGGAAC AATGACAATG AAACAGCATT
      121 GTGGTCATCA ACAACTACGG CAACAAAATT CGGGCTGCAT ACATCGTCAA CTAAATGAAA
      181 ATCGTTTTAG TTGCATGCGA AGATTTAGAG AAAACAGTCC ACGCTGTCAC AGACATCATG
      241 CATCTAAAGG ACCTCATGAA CATCACCATC ATAGTTCAAG AGGAACTCAT CATCATCATA
      301 GAGTAGGCAG AAAACATCAT TGCCATCATA ATGGTTCCGG TGCCACAAAC TTCAGTTTGT
      361 TAAAGCAGGC CGGAGACGTC GAAGAGAACC CCGGACCGAT GGTGAGCAAG GGCGCCGAGC
      421 TGTTCACCGG CATCGTGCCC ATCCTGATCG AGCTGAATGG CGATGTGAAT GGCCACAAGT
      481 TCAGCGTGAG CGGCGAGGGC GAGGGCGATG CCACCTACGG CAAGCTGACC CTGAAGTTCA
      541 TCTGCACCAC CGGCAAGCTG CCTGTGCCCT GGCCCACCCT GGTGACCACC CTGAGCTACG
      601 GCGTGCAGTG CTTCTCACGC TACCCCGATC ACATGAAGCA GCACGACTTC TTCAAGAGCG
      661 CCATGCCTGA GGGCTACATC CAGGAGCGCA CCATCTTCTT CGAGGATGAC GGCAACTACA
      721 AGTCGCGCGC CGAGGTGAAG TTCGAGGGCG ATACCCTGGT GAATCGCATC GAGCTGACCG
      781 GCACCGATTT CAAGGAGGAT GGCAACATCC TGGGCAATAA GATGGAGTAC AACTACAACG
      841 CCCACAATGT GTACATCATG ACCGACAAGG CCAAGAATGG CATCAAGGTG AACTTCAAGA
      901 TCCGCCACAA CATCGAGGAT GGCAGCGTGC AGCTGGCCGA CCACTACCAG CAGAATACCC
      961 CCATCGGCGA TGGCCCTGTG CTGCTGCCCG ATAACCACTA CCTGTCCACC CAGAGCGCCC
     1021 TGTCCAAGGA CCCCAACGAG AAGCGCGATC ACATGATCTA CTTCGGCTTC GTGACCGCCG
     1081 CCGCCATCAC CCACGGCATG GATGAGCTGT ACAAGGGTTC CGGTGCCACA AACTTCAGTT
     1141 TGTTAAAGCA GGCCGGAGAC GTCGAAGAGA ACCCCGGACC GATGGCCAAG TTGACCAGTG
     1201 CCGTTCCGGT GCTCACCGCG CGCGACGTCG CCGGAGCGGT CGAGTTCTGG ACCGACCGGC
     1261 TCGGGTTCTC CCGGGACTTC GTGGAGGACG ACTTCGCCGG TGTGGTCCGG GACGACGTGA
     1321 CCCTGTTCAT CAGCGCGGTC CAGGACCAGG TGGTGCCGGA CAACACCCTG GCCTGGGTGT
     1381 GGGTGCGCGG CCTGGACGAG CTGTACGCCG AGTGGTCGGA GGTCGTGTCC ACGAACTTCC
     1441 GGGACGCCTC CGGGCCGGCC ATGACCGAGA TCGGCGAGCA GCCGTGGGGG CGGGAGTTCG
     1501 CCCTGCGCGA CCCGGCCGGC AACTGCGTGC ACTTCGTGGC CGAGGAGCAG GACTAAAATC
     1561 TTATAACAAC ATCCTAAAGT ATTATAACAC CAAAGAATTG CAACAACATC GTCAACTCAA
     1621 AACAACATCT ATAAAAAATA AGACATAAGT CCAACAATTG TCGAAATAAC ATCAGAAAAT
     1681 AAAATCTTGA ACATCATGAA AAAAATCTTG ATTGAAAAAA AGACATATGT GTATTATTCC
     1741 AATTCCTTTC AGATTATTTT TTTTTTCAAG TTTCTGATAA TTTTATTTCA CATGTTTTCA
     1801 AACGTTTTTA CTGATACGCA TTAAAGTAGC AATAACACCC
//
```

Supplementary Data 4. The genome sequence of HaloTag-knock-in allele.

```
LOCUS       genome_sequence_of_HaloTag-KI-allele      1576 bp    DNA     linear   UNA 06-2-2018
DEFINITION
FEATURES             Location/Qualifiers
     primer_bind     1..20
                     /PCR_conditions="60.04 'C"
                     /label="YM_Pv.00443-F1"
     CDS             1..332
                     /cds_type="ORF"
                     /label="Pv.00443"
     microhomology   290..329
                     /label="Left homology"
     Site            333..398
                     /site_type="restriction site"
                     /label="P2A"
     CDS             399..1289
                     /cds_type="ORF"
                     /label="HaloTag (stop less)"
     microhomology   1290..1329
                     /label="Right homology"
     primer          complement(1552..1576)
                     /PCR_conditions="60.16 'C"
                     /label="YM_Pv.00443-R1"
ORIGIN
        1 GCCAAAGCGA GCCAATTCAA CCACCTCGCT ATTCCTTCGC TGAGCGACAA GAATCATCTA
       61 GTCGAGATCA TGGAGAAGGT CCATCACACG ATTGTGGAAC AATGACAATG AAACAGCATT
      121 GTGGTCATCA ACAACTACGG CAACAAAATT CGGGCTGCAT ACATCGTCAA CTAAATGAAA
      181 ATCGTTTTAG TTGCATGCGA AGATTTAGAG AAAACAGTCC ACGCTGTCAC AGACATCATG
      241 CATCTAAAGG ACCTCATGAA CATCACCATC ATAGTTCAAG AGGAACTCAT CATCATCATA
      301 GAGTAGGCAG AAAACATCAT TGCCATCATA ATGGTTCCGG TGCCACAAAC TTCAGTTTGT
      361 TAAAGCAGGC CGGAGACGTC GAAGAGAACC CCGGACCGAT GGCAGAAATC GGTACTGGCT
      421 TTCCATTCGA CCCCCATTAT GTGGAAGTCC TGGGCGAGCG CATGCACTAC GTCGATGTTG
      481 GTCCGCGCGA TGGCACCCCT GTGCTGTTCC TGCACGGTAA CCCGACCTCC TCCTACGTGT
      541 GGCGCAACAT CATCCCGCAT GTTGCACCGA CCCATCGCTG CATTGCTCCA GACCTGATCG
      601 GTATGGGCAA ATCCGACAAA CCAGACCTGG GTTATTTCTT CGACGACCAC GTCCGCTTCA
      661 TGGATGCCTT CATCGAAGCC CTGGGTCTGG AAGAGGTCGT CCTGGTCATT CACGACTGGG
      721 GCTCCGCTCT GGGTTTCCAC TGGGCCAAGC GCAATCCAGA GCGCGTCAAA GGTATTGCAT
      781 TTATGGAGTT CATCCGCCCT ATCCCGACCT GGGACGAATG GCCAGAATTT GCCCGCGAGA
      841 CCTTCCAGGC CTTCCGCACC ACCGACGTCG GCCGCAAGCT GATCATCGAT CAGAACGTTT
      901 TTATCGAGGG TACGCTGCCG ATGGGTGTCG TCCGCCCGCT GACTGAAGTC GAGATGGACC
      961 ATTACCGCGA GCCGTTCCTG AATCCTGTTG ACCGCGAGCC ACTGTGGCGC TTCCCAAACG
     1021 AGCTGCCAAT CGCCGGTGAG CCAGCGAACA TCGTCGCGCT GGTCGAAGAA TACATGGACT
     1081 GGCTGCACCA GTCCCCTGTC CCGAAGCTGC TGTTCTGGGG CACCCCAGGC GTTCTGATCC
     1141 CACCGGCCGA AGCCGCTCGC CTGGCCAAAA GCCTGCCTAA CTGCAAGGCT GTGGACATCG
     1201 GCCCGGGTCT GAATCTGCTG CAAGAAGACA ACCCGGACCT GATCGGCAGC GAGATCGCGC
     1261 GCTGGCTGTC GACGCTCGAG ATTTCCGGCT AAAATCTTAT AACAACATCC TAAAGTATTA
     1321 TAACACCAAA GAATTGCAAC AACATCGTCA ACTCAAAACA ACATCTATAA AAAATAAGAC
     1381 ATAAGTCCAA CAATTGTCGA AATAACATCA GAAAATAAAA TCTTGAACAT CATGAAAAAA
     1441 ATCTTGATTG AAAAAAAGAC ATATGTGTAT TATTCCAATT CCTTTCAGAT TATTTTTTTT
     1501 TTCAAGTTTC TGATAATTTT ATTTCACATG TTTTCAAACG TTTTTACTGA TACGCATTAA
     1561 AGTAGCAATA ACACCC
//
```

Supplementary Data 5. The genome sequence of BlaR-knock-in allele.

```
LOCUS       genome_sequence_of_BlaR-KI-allele      1081 bp    DNA     linear   UNA 06-2-2018
DEFINITION
FEATURES             Location/Qualifiers
     primer_bind     1..20
                     /PCR_conditions="60.04 'C"
                     /label="YM_Pv.00443-F1"
     CDS             1..332
                     /cds_type="ORF"
                     /label="Pv.00443"
     microhomology   290..329
                     /label="Left homology"
     Site            333..398
                     /site_type="restriction site"
                     /label="P2A"
     CDS             399..794
                     /cds_type="ORF"
                     /label="Blasticidin (stop less)"
     microhomology   795..834
                     /label="Right homology"
     primer          complement(1057..1081)
                     /PCR_conditions="60.16 'C"
                     /label="YM_Pv.00443-R1"
ORIGIN
        1 GCCAAAGCGA GCCAATTCAA CCACCTCGCT ATTCCTTCGC TGAGCGACAA GAATCATCTA
       61 GTCGAGATCA TGGAGAAGGT CCATCACACG ATTGTGGAAC AATGACAATG AAACAGCATT
      121 GTGGTCATCA ACAACTACGG CAACAAAATT CGGGCTGCAT ACATCGTCAA CTAAATGAAA
      181 ATCGTTTTAG TTGCATGCGA AGATTTAGAG AAAACAGTCC ACGCTGTCAC AGACATCATG
      241 CATCTAAAGG ACCTCATGAA CATCACCATC ATAGTTCAAG AGGAACTCAT CATCATCATA
      301 GAGTAGGCAG AAAACATCAT TGCCATCATA ATGGTTCCGG TGCCACAAAC TTCAGTTTGT
      361 TAAAGCAGGC CGGAGACGTC GAAGAGAACC CCGGACCGAT GGCCAAGCCT TTGTCTCAAG
      421 AAGAATCCAC CCTCATTGAA AGAGCAACGG CTACAATCAA CAGCATCCCC ATCTCTGAAG
      481 ACTACAGCGT CGCCAGCGCA GCTCTCTCTA GCGACGGCCG CATCTTCACT GGTGTCAATG
      541 TATATCATTT TACTGGGGGA CCTTGCGCAG AACTCGTGGT GCTGGGCACT GCTGCTGCTG
      601 CGGCAGCTGG CAACCTGACT TGTATCGTCG CGATCGGAAA TGAGAACAGG GGCATCTTGA
      661 GcCCCTGCGG ACGGTGCCGA CAGGTTCTTC TCGATCTGCA TCCTGGGATC AAAGCCATAG
      721 TGAAGGACAG TGATGGACAG CCGACGGCAG TTGGGATTCG TGAATTGCTG CCCTCTGGTT
      781 ATGTGTGGGA GGGCTAAAAT CTTATAACAA CATCCTAAAG TATTATAACA CCAAAGAATT
      841 GCAACAACAT CGTCAACTCA AAACAACATC TATAAAAAAT AAGACATAAG TCCAACAATT
      901 GTCGAAATAA CATCAGAAAA TAAAATCTTG AACATCATGA AAAAAATCTT GATTGAAAAA
      961 AAGACATATG TGTATTATTC CAATTCCTTT CAGATTATTT TTTTTTTCAA GTTTCTGATA
     1021 ATTTTATTTC ACATGTTTTC AAACGTTTTT ACTGATACGC ATTAAAGTAG CAATAACACC
     1081 C
//
```

Supplementary Data 6. The genome sequence of GCaMP3-knock-in allele.

```
LOCUS       genome_sequence_of_GCaMP3-KI-allele      2035 bp    DNA     linear   UNA 06-2-2018
DEFINITION
FEATURES             Location/Qualifiers
     primer_bind     1..20
                     /PCR_conditions="60.04 'C"
                     /label="YM_Pv.00443-F1"
     CDS             1..332
                     /cds_type="ORF"
                     /label="Pv.00443"
     microhomology   290..329
                     /label="left microhomology of Pv.00443"
     Site            333..398
                     /site_type="restriction site"
                     /label="P2A"
     CDS             399..1751
                     /cds_type="ORF"
                     /label="GCaMP3"
     microhomology   1749..1788
                     /label="Right microhomology of Pv.00443"
     primer          complement(2011..2035)
                     /PCR_conditions="60.16 'C"
                     /label="YM_Pv.00443-R1"
ORIGIN
        1 GCCAAAGCGA GCCAATTCAA CCACCTCGCT ATTCCTTCGC TGAGCGACAA GAATCATCTA
       61 GTCGAGATCA TGGAGAAGGT CCATCACACG ATTGTGGAAC AATGACAATG AAACAGCATT
      121 GTGGTCATCA ACAACTACGG CAACAAAATT CGGGCTGCAT ACATCGTCAA CTAAATGAAA
      181 ATCGTTTTAG TTGCATGCGA AGATTTAGAG AAAACAGTCC ACGCTGTCAC AGACATCATG
      241 CATCTAAAGG ACCTCATGAA CATCACCATC ATAGTTCAAG AGGAACTCAT CATCATCATA
      301 GAGTAGGCAG AAAACATCAT TGCCATCATA ATGGTTCCGG TGCCACAAAC TTCAGTTTGT
      361 TAAAGCAGGC CGGAGACGTC GAAGAGAACC CCGGACCGAT GGGTTCTCAT CATCATCATC
      421 ATCATGGTAT GGCTAGCATG ACTGGTGGAC AGCAAATGGG TCGGGATCTG TACGACGATG
      481 ACGATAAGGA TCTCGCCACC ATGGTCGACT CATCACGTCG TAAGTGGAAT AAGACAGGTC
      541 ACGCAGTCAG AGCTATAGGT CGGCTGAGCT CACTCGAGAA CGTCTATATC AAGGCCGACA
      601 AGCAGAAGAA CGGCATCAAG GCGAACTTCA AGATCCGCCA CAACATCGAG GACGGCGGCG
      661 TGCAGCTCGC CTACCACTAC CAGCAGAACA CCCCCATCGG CGACGGCCCC GTGCTGCTGC
      721 CCGACAACCA CTACCTGAGC GTGCAGTCCA AACTTTCGAA AGACCCCAAC GAGAAGCGCG
      781 ATCACATGGT CCTGCTGGAG TTCGTGACCG CCGCCGGGAT CACTCTCGGC ATGGACGAGC
      841 TGTACAAGGG CGGTACCGGA GGGAGCATGG TGAGCAAGGG CGAGGAGCTG TTCACCGGGG
      901 TGGTGCCCAT CCTGGTCGAG CTGGACGGCG ACGTAAACGG CCACAAGTTC AGCGTGTCCG
      961 GCGAGGGCGA GGGCGATGCC ACCTACGGCA AGCTGACCCT GAAGTTCATC TGCACCACCG
     1021 GCAAGCTGCC CGTGCCCTGG CCCACCCTCG TGACCACCCT GACCTACGGC GTGCAGTGCT
     1081 TCAGCCGCTA CCCCGACCAC ATGAAGCAGC ACGACTTCTT CAAGTCCGCC ATGCCCGAAG
     1141 GCTACATCCA GGAGCGCACC ATCTTCTTCA AGGACGACGG CAACTACAAG ACCCGCGCCG
     1201 AGGTGAAGTT CGAGGGCGAC ACCCTGGTGA ACCGCATCGA GCTGAAGGGC ATCGACTTCA
     1261 AGGAGGACGG CAACATCCTG GGGCACAAGC TGGAGTACAA CACGCGTGAC CAACTGACTG
     1321 AAGAGCAGAT CGCAGAATTT AAAGAGGCTT TCTCCCTATT TGACAAGGAC GGGGATGGGA
     1381 CAATAACAAC CAAGGAGCTG GGGACGGTGA TGCGGTCTCT GGGGCAGAAC CCCACAGAAG
     1441 CAGAGCTGCA GGACATGATC AATGAAGTAG ATGCCGACGG TGACGGCACA ATCGACTTCC
     1501 CTGAGTTCCT GACAATGATG GCAAGAAAAA TGAAAGACAC AGACAGTGAA GAAGAAATTA
     1561 GAGAAGCGTT CCGTGTGTTT GATAAGGATG GCAATGGCTA CATCAGTGCA GCAGAGCTTC
     1621 GCCACGTGAT GACAAACCTT GGAGAGAAGT TAACAGATGA AGAGGTTGAT GAAATGATCA
     1681 GGGAAGCAGA CATCGATGGG GATGGTCAGG TAAACTACGA AGAGTTTGTA CAAATGATGA
     1741 CAGCGAAGTA AAATCTTATA ACAACATCCT AAAGTATTAT AACACCAAAG AATTGCAACA
     1801 ACATCGTCAA CTCAAAACAA CATCTATAAA AAATAAGACA TAAGTCCAAC AATTGTCGAA
     1861 ATAACATCAG AAAATAAAAT CTTGAACATC ATGAAAAAAA TCTTGATTGA AAAAAAGACA
     1921 TATGTGTATT ATTCCAATTC CTTTCAGATT ATTTTTTTTT TCAAGTTTCT GATAATTTTA
     1981 TTTCACATGT TTTCAAACGT TTTTACTGAT ACGCATTAAA GTAGCAATAA CACCC
//
```

Supplementary Data 7. The genome sequence of ZeoR-knock-in allele.

```
LOCUS       genome_sequence_of_ZeoR-KI-allele      1062 bp    DNA     linear   UNA 06-2-2018
DEFINITION
FEATURES             Location/Qualifiers
     primer_bind     1..20
                     /PCR_conditions="60.04 'C"
                     /label="YM_Pv.00443-F1"
     CDS             1..332
                     /cds_type="ORF"
                     /label="Pv.00443"
     microhomology   290..329
                     /label="left microhomology of Pv.00443"
     Site            333..398
                     /site_type="restriction site"
                     /label="P2A"
     CDS             399..773
                     /cds_type="ORF"
                     /label="Zeocin"
     microhomology   771..810
                     /label="right microhomology of Pv.00443"
     primer          complement(1033..1057)
                     /PCR_conditions="60.16 'C"
                     /label="YM_Pv.00443-R1"
ORIGIN
        1 GCCAAAGCGA GCCAATTCAA CCACCTCGCT ATTCCTTCGC TGAGCGACAA GAATCATCTA
       61 GTCGAGATCA TGGAGAAGGT CCATCACACG ATTGTGGAAC AATGACAATG AAACAGCATT
      121 GTGGTCATCA ACAACTACGG CAACAAAATT CGGGCTGCAT ACATCGTCAA CTAAATGAAA
      181 ATCGTTTTAG TTGCATGCGA AGATTTAGAG AAAACAGTCC ACGCTGTCAC AGACATCATG
      241 CATCTAAAGG ACCTCATGAA TATCACCATC ATAGTTCAAG AGGAACTCAT CATCATCATA
      301 GAGTAGGCAG AAAACATCAT TGCCATCATA ATGGTTCCGG TGCCACAAAC TTCAGTTTGT
      361 TAAAGCAGGC CGGAGACGTC GAAGAGAACC CCGGACCGAT GGCCAAGTTG ACCAGTGCCG
      421 TTCCGGTGCT CACCGCGCGC GACGTCGCCG GAGCGGTCGA GTTCTGGACC GACCGGCTCG
      481 GGTTCTCCCG GGACTTCGTG GAGGACGACT TCGCCGGTGT GGTCCGGGAC GACGTGACCC
      541 TGTTCATCAG CGCGGTCCAG GACCAGGTGG TGCCGGACAA CACCCTGGCC TGGGTGTGGG
      601 TGCGCGGCCT GGACGAGCTG TACGCCGAGT GGTCGGAGGT CGTGTCCACG AACTTCCGGG
      661 ACGCCTCCGG GCCGGCCATG ACCGAGATCG GCGAGCAGCC GTGGGGGCGG GAGTTCGCCC
      721 TGCGCGACCC GGCCGGCAAC TGCGTGCACT TCGTGGCCGA GGAGCAGGAC TAAAATCTTA
      781 TAACAACATC CTAAAGTATT ATAACACCAA AGAATTGCAA CAACATCGTC AACTCAAAAC
      841 AACATCTATA AAAAATAAGA CATAAGTCCA ACAATTGTCG AAATAACATC AGAAAATAAA
      901 ATCTTGAACA TCATGAAAAA AATCTTGATT GAAAAAAAGA CATATGTGTA TTATTCCAAT
      961 TCCTTTCAGA TTATTTTTTT TTTCAAGTTT CTGATAATTT TATTTCACAT GTTTTCAAAC
     1021 GTTTTTACTG ATACGCATTA AAGTAGCAAT AACACCCAAT AA
//
```

Supplementary Data 8. The full-length sequence of pPvU6b-DmtRNA-AcGFP1#1 plasmid.

```
LOCUS       pPvU6b-DmtRNA-AcGFP1#1_sequence      3099 bp    DNA     circular UNA
FEATURES             Location/Qualifiers
     promoter        62..747
                     /label="PvU6b promoter"
     tRNA            748..844
                     /label="Dm tRNA_gly"
     misc_feature    845..864
                     /feature_type="Zinc finger"
                     /label="AcGFP1#1"
     misc_feature    865..940
                     /feature_type="Zinc finger"
                     /label="gRNA core"
     terminator      941..946
                     /label="pol III terminator"
     promoter        complement(1066..1187)
                     /label="lac promoter"
     primer          complement(1110..1139)
                     /label="lac promoter-R"
     rep_origin      1407..2026
                     /label="pUC ori"
     promoter        2647..2724
                     /label="EM7 promoter"
     CDS             2725..3099
                     /label="bleo"
                     /gene="bleo"
                     /translation="MRKEKIPHQEIVSVNNSEELVKKAIEGDALRIGSGDTVKHEEAV
                     SPFAAKLFSNITGSQRYVLIAVRHTQPATVDESRKAAIFHHDIRQAGIAMGHDEILAV
                     GHARLEPGEQFGWREPLMLFVQIILIDKTGFHPSTCSLDAMFRLVVEWAGSRIKRMQP
                     PHCISHDGYFLGRSKVR*"
                     /label="Zeo+/Bleo+"
ORIGIN
        1 CCGACGCCGA CCAACACCGC CGGTCCGACG CGGCCCGACG GGTCCGAGGG GGGTCGACCT
       61 CAAAATTCTA AAGCTGACTT TTTAAATTTT TTACTTTTCT TTAATTCTTT CAATTAAAAT
      121 TTAAATAAAA AATTCAAACA TTTTTTCTCT TCTTTTTATA TTTAGTTTCA GTTCATTTTG
      181 TACTCAAATA TTGACATAAT AAAATAAATT TCATATTAAT TAATCGAAAT ATTTGTGTCA
      241 TAAATAAATA ACTAAGAAAA AAAAGGGTTT GTCATGTCGT GTGTCGAATG TCCAAGTGAA
      301 AATGTTTGTT TAACAAATTG AAATTTCATT TAATTAACAC CGCATTGTGC ATAAGAGAAA
      361 GGAAAGAAAG AAAAGAAAAT TAGCTTGGTC TGATTACAAA TAAAAAAGGG TTCAGGTAAT
      421 AAAAAAAATT CCTTTCACTC GTTCATCATC AAAATCACTT CTGGTTTACA AGTTGAGGTC
      481 AAATGAAGTC AAATGAATAT CAAAAAATAA ATTGCAGTTG ATTGACTGTT TATAGCAGCT
      541 CATATATGAT CAGTAGACGA TTATCGATAA ATTTTTATTA AGCACGAGCT GTTTGAGGTC
      601 AATTTCAGTT GATTGCAGTG CAAAACCAGC TGCAAAATTG TTTTCATATT TCACTTGTTC
      661 CAAATGTTTT GTTTTGTTTC ATTTTCCATT TCGTTTCATT TCAATAGCAG CAGTGGTTTA
      721 AGTAGCATTT CTGCCAAATA CAAAATTGGG CTTTGAGTGT GTGTAGACAT CAAGCATCGG
      781 TGGTTCAGTG GTAGAATGCT CGCCTGCCAC GCGGGCGGCC CGGGTTCGAT TCCCGGCCGA
      841 TGCAAAGGGC GCCGAGCTGT TCACGTTTTA GAGCTAGAAA TAGCAAGTTA AAATAAGGCT
      901 AGTCCGTTAT CAACTTGAAA AAGTGGCACC GAGTCGGTGC TTTTTTatcg aattcctgca
      961 gcccggGGGA TCCACTAGTT CTAGAGCGGC CGCCACCGCG GTGGAGCTCC AGCTTTTGTT
     1021 CCCTTTAGTG AGGGTTAATT TCGAGCTTGG CGTAATCATG GTCATAGCTG TTTCCTGTGT
     1081 GAAATTGTTA TCCGCTCACA ATTCCACACA ACATACGAGC CGGAAGCATA AAGTGTAAAG
     1141 CCTGGGGTGC CTAATGAGTG AGCTAACTCA CATTAATTGC GTTGCGCTCA CTGCCCGCTT
     1201 TCCAGTCGGG AAACCTGTCG TGCCAGCTGC ATTAATGAAT CGGCCAACGC GCGGGGAGAG
     1261 GCGGTTTGCG TATTGGGCGC TCTTCCGCTT CCTCGCTCAC TGACTCGCTG CGCTCGGTCG
     1321 TTCGGCTGCG GCGAGCGGTA TCAGCTCACT CAAAGGCGGT AATACGGTTA TCCACAGAAT
     1381 CAGGGGATAA CGCAGGAAAG AACATGCGCG TTGCTGGCGT TTTTCCATAG GCTCCGCCCC
     1441 CCTGACGAGC ATCACAAAAA TCGACGCTCA AGTCAGAGGT GGCGAAACCC GACAGGACTA
     1501 TAAAGATACC AGGCGTTTCC CCCTGGAAGC TCCCTCGTGC GCTCTCCTGT TCCGACCCTG
     1561 CCGCTTACCG GATACCTGTC CGCCTTTCTC CCTTCGGGAA GCGTGGCGCT TTCTCATAGC
     1621 TCACGCTGTA GGTATCTCAG TTCGGTGTAG GTCGTTCGCT CCAAGCTGGG CTGTGTGCAC
     1681 GAACCCCCCG TTCAGCCCGA CCGCTGCGCC TTATCCGGTA ACTATCGTCT TGAGTCCAAC
     1741 CCGGTAAGAC ACGACTTATC GCCACTGGCA GCAGCCACTG GTAACAGGAT TAGCAGAGCG
     1801 AGGTATGTAG GCGGTGCTAC AGAGTTCTTG AAGTGGTGGC CTAACTACGG CTACACTAGA
     1861 AGAACAGTAT TTGGTATCTG CGCTCTGCTG AAGCCAGTTA CCTTCGGAAA AAGAGTTGGT
     1921 AGCTCTTGAT CCGGCAAACA AACCACCGCT GGTAGCGGTG GTTTTTTTGT TTGCAAGCAG
     1981 CAGATTACGC GCAGAAAAAA AGGATCTCAA GAAGATCCTT TGATCTTTTC TACGGGGTCT
     2041 GACGCTCAGT GGAACGAAAA CTCACGTTAA GGGATTTTGG TCATGATGAT AAACAATGTA
     2101 TGGTGCTAAT GTTGCTTCAA CAACAATTCT GTTGAACTGT GTTTTCATGT TTGCCAACAA
     2161 GCACCTTTAT ACTCGGTGGC CTCCCCACCA CCAACTTTTT TGCACTGCAA AAAAACACGC
     2221 TTTTGCACGC GGGCCCATAC ATAGTACAAA CTCTACGTTT CGTAGACTAT TTTACATAAA
     2281 TAGTCTACAC CGTTGTATAC GCTCCAAATA CACTACCACA CATTGAACCT TTTTGCAGTG
     2341 CAAAAAAGTA CGTGTCGGCA GTCACGTAGG CCGGCCTTAT CGGGTCGCGT CCTGTCACGT
     2401 ACGAATCACA TTATCGGACC GGACGAGTGT TGTCTTATCG TGACAGGACG CCAGCTTCCT
     2461 GTGTTGCTAA CCGCAGCCGG ACGCAACTCC TTATCGGAAC AGGACGCGCC TCCATATCAG
     2521 CCGCGCGTTA TCTCATGCGC GTGACCGGAC ACGAGGCGCC CGTCCCGCTT ATCGCGCCTA
     2581 TAAATACAGC CCGCAACGAT CTGGTAAACA CAGTTGAACA GCATCTGTTC GAATTAATTC
     2641 GGATCTCTGC AGCACGTGTT GACAATTAAT CATCGGCATA GTATATCGGC ATAGTATAAT
     2701 ACGACTCACT ATAGGAGGGC CACCATGGCC AAGTTGACCA GTGCCGTTCC GGTGCTCACC
     2761 GCGCGCGACG TCGCCGGAGC GGTCGAGTTC TGGACCGACC GGCTCGGGTT CTCCCGGGAC
     2821 TTCGTGGAGG ACGACTTCGC CGGTGTGGTC CGGGACGACG TGACCCTGTT CATCAGCGCG
     2881 GTCCAGGACC AGGTGGTGCC GGACAACACC CTGGCCTGGG TGTGGGTGCG CGGCCTGGAC
     2941 GAGCTGTACG CCGAGTGGTC GGAGGTCGTG TCCACGAACT TCCGGGACGC CTCCGGGCCG
     3001 GCCATGACCG AGATCGGCGA GCAGCCGTGG GGGCGGGAGT TCGCCCTGCG CGACCCGGCC
     3061 GGCAACTGCG TGCACTTCGT GGCCGAGGAG CAGGACTGA
//
```

Supplementary Data 9. The full-length sequence of pPvU6b-DmtRNA-AcGFP1#3 plasmid.

```
LOCUS       pPvU6b-DmtRNA-AcGFP1#3_sequence      3099 bp    DNA     circular UNA
FEATURES             Location/Qualifiers
     promoter        62..747
                     /label="PvU6b promoter"
     tRNA            748..844
                     /label="Dm tRNA_gly"
     misc_feature    845..864
                     /feature_type="Zinc finger"
                     /label="AcGFP1#3"
     misc_feature    865..940
                     /feature_type="Zinc finger"
                     /label="gRNA core"
     terminator      941..946
                     /label="pol III terminator"
     promoter        complement(1066..1187)
                     /label="lac promoter"
     primer          complement(1110..1139)
                     /label="lac promoter-R"
     rep_origin      1407..2026
                     /label="pUC ori"
     promoter        2647..2724
                     /label="EM7 promoter"
     CDS             2725..3099
                     /label="bleo"
                     /gene="bleo"
                     /translation="MRKEKIPHQEIVSVNNSEELVKKAIEGDALRIGSGDTVKHEEAV
                     SPFAAKLFSNITGSQRYVLIAVRHTQPATVDESRKAAIFHHDIRQAGIAMGHDEILAV
                     GHARLEPGEQFGWREPLMLFVQIILIDKTGFHPSTCSLDAMFRLVVEWAGSRIKRMQP
                     PHCISHDGYFLGRSKVR*"
                     /label="Zeo+/Bleo+"
ORIGIN
        1 CCGACGCCGA CCAACACCGC CGGTCCGACG CGGCCCGACG GGTCCGAGGG GGGTCGACCT
       61 CAAAATTCTA AAGCTGACTT TTTAAATTTT TTACTTTTCT TTAATTCTTT CAATTAAAAT
      121 TTAAATAAAA AATTCAAACA TTTTTTCTCT TCTTTTTATA TTTAGTTTCA GTTCATTTTG
      181 TACTCAAATA TTGACATAAT AAAATAAATT TCATATTAAT TAATCGAAAT ATTTGTGTCA
      241 TAAATAAATA ACTAAGAAAA AAAAGGGTTT GTCATGTCGT GTGTCGAATG TCCAAGTGAA
      301 AATGTTTGTT TAACAAATTG AAATTTCATT TAATTAACAC CGCATTGTGC ATAAGAGAAA
      361 GGAAAGAAAG AAAAGAAAAT TAGCTTGGTC TGATTACAAA TAAAAAAGGG TTCAGGTAAT
      421 AAAAAAAATT CCTTTCACTC GTTCATCATC AAAATCACTT CTGGTTTACA AGTTGAGGTC
      481 AAATGAAGTC AAATGAATAT CAAAAAATAA ATTGCAGTTG ATTGACTGTT TATAGCAGCT
      541 CATATATGAT CAGTAGACGA TTATCGATAA ATTTTTATTA AGCACGAGCT GTTTGAGGTC
      601 AATTTCAGTT GATTGCAGTG CAAAACCAGC TGCAAAATTG TTTTCATATT TCACTTGTTC
      661 CAAATGTTTT GTTTTGTTTC ATTTTCCATT TCGTTTCATT TCAATAGCAG CAGTGGTTTA
      721 AGTAGCATTT CTGCCAAATA CAAAATTGGG CTTTGAGTGT GTGTAGACAT CAAGCATCGG
      781 TGGTTCAGTG GTAGAATGCT CGCCTGCCAC GCGGGCGGCC CGGGTTCGAT TCCCGGCCGA
      841 TGCAGTGAAT CGCATCGAGC TGACGTTTTA GAGCTAGAAA TAGCAAGTTA AAATAAGGCT
      901 AGTCCGTTAT CAACTTGAAA AAGTGGCACC GAGTCGGTGC TTTTTTatcg aattcctgca
      961 gcccggGGGA TCCACTAGTT CTAGAGCGGC CGCCACCGCG GTGGAGCTCC AGCTTTTGTT
     1021 CCCTTTAGTG AGGGTTAATT TCGAGCTTGG CGTAATCATG GTCATAGCTG TTTCCTGTGT
     1081 GAAATTGTTA TCCGCTCACA ATTCCACACA ACATACGAGC CGGAAGCATA AAGTGTAAAG
     1141 CCTGGGGTGC CTAATGAGTG AGCTAACTCA CATTAATTGC GTTGCGCTCA CTGCCCGCTT
     1201 TCCAGTCGGG AAACCTGTCG TGCCAGCTGC ATTAATGAAT CGGCCAACGC GCGGGGAGAG
     1261 GCGGTTTGCG TATTGGGCGC TCTTCCGCTT CCTCGCTCAC TGACTCGCTG CGCTCGGTCG
     1321 TTCGGCTGCG GCGAGCGGTA TCAGCTCACT CAAAGGCGGT AATACGGTTA TCCACAGAAT
     1381 CAGGGGATAA CGCAGGAAAG AACATGCGCG TTGCTGGCGT TTTTCCATAG GCTCCGCCCC
     1441 CCTGACGAGC ATCACAAAAA TCGACGCTCA AGTCAGAGGT GGCGAAACCC GACAGGACTA
     1501 TAAAGATACC AGGCGTTTCC CCCTGGAAGC TCCCTCGTGC GCTCTCCTGT TCCGACCCTG
     1561 CCGCTTACCG GATACCTGTC CGCCTTTCTC CCTTCGGGAA GCGTGGCGCT TTCTCATAGC
     1621 TCACGCTGTA GGTATCTCAG TTCGGTGTAG GTCGTTCGCT CCAAGCTGGG CTGTGTGCAC
     1681 GAACCCCCCG TTCAGCCCGA CCGCTGCGCC TTATCCGGTA ACTATCGTCT TGAGTCCAAC
     1741 CCGGTAAGAC ACGACTTATC GCCACTGGCA GCAGCCACTG GTAACAGGAT TAGCAGAGCG
     1801 AGGTATGTAG GCGGTGCTAC AGAGTTCTTG AAGTGGTGGC CTAACTACGG CTACACTAGA
     1861 AGAACAGTAT TTGGTATCTG CGCTCTGCTG AAGCCAGTTA CCTTCGGAAA AAGAGTTGGT
     1921 AGCTCTTGAT CCGGCAAACA AACCACCGCT GGTAGCGGTG GTTTTTTTGT TTGCAAGCAG
     1981 CAGATTACGC GCAGAAAAAA AGGATCTCAA GAAGATCCTT TGATCTTTTC TACGGGGTCT
     2041 GACGCTCAGT GGAACGAAAA CTCACGTTAA GGGATTTTGG TCATGATGAT AAACAATGTA
     2101 TGGTGCTAAT GTTGCTTCAA CAACAATTCT GTTGAACTGT GTTTTCATGT TTGCCAACAA
     2161 GCACCTTTAT ACTCGGTGGC CTCCCCACCA CCAACTTTTT TGCACTGCAA AAAAACACGC
     2221 TTTTGCACGC GGGCCCATAC ATAGTACAAA CTCTACGTTT CGTAGACTAT TTTACATAAA
     2281 TAGTCTACAC CGTTGTATAC GCTCCAAATA CACTACCACA CATTGAACCT TTTTGCAGTG
     2341 CAAAAAAGTA CGTGTCGGCA GTCACGTAGG CCGGCCTTAT CGGGTCGCGT CCTGTCACGT
     2401 ACGAATCACA TTATCGGACC GGACGAGTGT TGTCTTATCG TGACAGGACG CCAGCTTCCT
     2461 GTGTTGCTAA CCGCAGCCGG ACGCAACTCC TTATCGGAAC AGGACGCGCC TCCATATCAG
     2521 CCGCGCGTTA TCTCATGCGC GTGACCGGAC ACGAGGCGCC CGTCCCGCTT ATCGCGCCTA
     2581 TAAATACAGC CCGCAACGAT CTGGTAAACA CAGTTGAACA GCATCTGTTC GAATTAATTC
     2641 GGATCTCTGC AGCACGTGTT GACAATTAAT CATCGGCATA GTATATCGGC ATAGTATAAT
     2701 ACGACTCACT ATAGGAGGGC CACCATGGCC AAGTTGACCA GTGCCGTTCC GGTGCTCACC
     2761 GCGCGCGACG TCGCCGGAGC GGTCGAGTTC TGGACCGACC GGCTCGGGTT CTCCCGGGAC
     2821 TTCGTGGAGG ACGACTTCGC CGGTGTGGTC CGGGACGACG TGACCCTGTT CATCAGCGCG
     2881 GTCCAGGACC AGGTGGTGCC GGACAACACC CTGGCCTGGG TGTGGGTGCG CGGCCTGGAC
     2941 GAGCTGTACG CCGAGTGGTC GGAGGTCGTG TCCACGAACT TCCGGGACGC CTCCGGGCCG
     3001 GCCATGACCG AGATCGGCGA GCAGCCGTGG GGGCGGGAGT TCGCCCTGCG CGACCCGGCC
     3061 GGCAACTGCG TGCACTTCGT GGCCGAGGAG CAGGACTGA
//
```

Supplementary Data 10. The full-length sequence of pPvU6b-DmtRNA-Pv.00443#1 plasmid.

```
LOCUS       pPvU6b-DmtRNA-Pv.00443#1_sequence      3099 bp    DNA     circular UNA
FEATURES             Location/Qualifiers
     promoter        62..747
                     /label="PvU6b promoter"
     tRNA            748..844
                     /label="Dm tRNA_gly"
     misc_feature    845..864
                     /feature_type="Zinc finger"
                     /label="Pv.00443#1"
     misc_feature    865..940
                     /feature_type="Zinc finger"
                     /label="gRNA core"
     terminator      941..946
                     /label="pol III terminator"
     promoter        complement(1066..1187)
                     /label="lac promoter"
     primer          complement(1110..1139)
                     /label="lac promoter-R"
     rep_origin      1407..2026
                     /label="pUC ori"
     promoter        2647..2724
                     /label="EM7 promoter"
     CDS             2725..3099
                     /label="bleo"
                     /gene="bleo"
                     /translation="MRKEKIPHQEIVSVNNSEELVKKAIEGDALRIGSGDTVKHEEAV
                     SPFAAKLFSNITGSQRYVLIAVRHTQPATVDESRKAAIFHHDIRQAGIAMGHDEILAV
                     GHARLEPGEQFGWREPLMLFVQIILIDKTGFHPSTCSLDAMFRLVVEWAGSRIKRMQP
                     PHCISHDGYFLGRSKVR*"
                     /label="Zeo+/Bleo+"
ORIGIN
        1 CCGACGCCGA CCAACACCGC CGGTCCGACG CGGCCCGACG GGTCCGAGGG GGGTCGACCT
       61 CAAAATTCTA AAGCTGACTT TTTAAATTTT TTACTTTTCT TTAATTCTTT CAATTAAAAT
      121 TTAAATAAAA AATTCAAACA TTTTTTCTCT TCTTTTTATA TTTAGTTTCA GTTCATTTTG
      181 TACTCAAATA TTGACATAAT AAAATAAATT TCATATTAAT TAATCGAAAT ATTTGTGTCA
      241 TAAATAAATA ACTAAGAAAA AAAAGGGTTT GTCATGTCGT GTGTCGAATG TCCAAGTGAA
      301 AATGTTTGTT TAACAAATTG AAATTTCATT TAATTAACAC CGCATTGTGC ATAAGAGAAA
      361 GGAAAGAAAG AAAAGAAAAT TAGCTTGGTC TGATTACAAA TAAAAAAGGG TTCAGGTAAT
      421 AAAAAAAATT CCTTTCACTC GTTCATCATC AAAATCACTT CTGGTTTACA AGTTGAGGTC
      481 AAATGAAGTC AAATGAATAT CAAAAAATAA ATTGCAGTTG ATTGACTGTT TATAGCAGCT
      541 CATATATGAT CAGTAGACGA TTATCGATAA ATTTTTATTA AGCACGAGCT GTTTGAGGTC
      601 AATTTCAGTT GATTGCAGTG CAAAACCAGC TGCAAAATTG TTTTCATATT TCACTTGTTC
      661 CAAATGTTTT GTTTTGTTTC ATTTTCCATT TCGTTTCATT TCAATAGCAG CAGTGGTTTA
      721 AGTAGCATTT CTGCCAAATA CAAAATTGGG CTTTGAGTGT GTGTAGACAT CAAGCATCGG
      781 TGGTTCAGTG GTAGAATGCT CGCCTGCCAC GCGGGCGGCC CGGGTTCGAT TCCCGGCCGA
      841 TGCATTATAA GATTTTAATT ATGAGTTTTA GAGCTAGAAA TAGCAAGTTA AAATAAGGCT
      901 AGTCCGTTAT CAACTTGAAA AAGTGGCACC GAGTCGGTGC TTTTTTatcg aattcctgca
      961 gcccggGGGA TCCACTAGTT CTAGAGCGGC CGCCACCGCG GTGGAGCTCC AGCTTTTGTT
     1021 CCCTTTAGTG AGGGTTAATT TCGAGCTTGG CGTAATCATG GTCATAGCTG TTTCCTGTGT
     1081 GAAATTGTTA TCCGCTCACA ATTCCACACA ACATACGAGC CGGAAGCATA AAGTGTAAAG
     1141 CCTGGGGTGC CTAATGAGTG AGCTAACTCA CATTAATTGC GTTGCGCTCA CTGCCCGCTT
     1201 TCCAGTCGGG AAACCTGTCG TGCCAGCTGC ATTAATGAAT CGGCCAACGC GCGGGGAGAG
     1261 GCGGTTTGCG TATTGGGCGC TCTTCCGCTT CCTCGCTCAC TGACTCGCTG CGCTCGGTCG
     1321 TTCGGCTGCG GCGAGCGGTA TCAGCTCACT CAAAGGCGGT AATACGGTTA TCCACAGAAT
     1381 CAGGGGATAA CGCAGGAAAG AACATGCGCG TTGCTGGCGT TTTTCCATAG GCTCCGCCCC
     1441 CCTGACGAGC ATCACAAAAA TCGACGCTCA AGTCAGAGGT GGCGAAACCC GACAGGACTA
     1501 TAAAGATACC AGGCGTTTCC CCCTGGAAGC TCCCTCGTGC GCTCTCCTGT TCCGACCCTG
     1561 CCGCTTACCG GATACCTGTC CGCCTTTCTC CCTTCGGGAA GCGTGGCGCT TTCTCATAGC
     1621 TCACGCTGTA GGTATCTCAG TTCGGTGTAG GTCGTTCGCT CCAAGCTGGG CTGTGTGCAC
     1681 GAACCCCCCG TTCAGCCCGA CCGCTGCGCC TTATCCGGTA ACTATCGTCT TGAGTCCAAC
     1741 CCGGTAAGAC ACGACTTATC GCCACTGGCA GCAGCCACTG GTAACAGGAT TAGCAGAGCG
     1801 AGGTATGTAG GCGGTGCTAC AGAGTTCTTG AAGTGGTGGC CTAACTACGG CTACACTAGA
     1861 AGAACAGTAT TTGGTATCTG CGCTCTGCTG AAGCCAGTTA CCTTCGGAAA AAGAGTTGGT
     1921 AGCTCTTGAT CCGGCAAACA AACCACCGCT GGTAGCGGTG GTTTTTTTGT TTGCAAGCAG
     1981 CAGATTACGC GCAGAAAAAA AGGATCTCAA GAAGATCCTT TGATCTTTTC TACGGGGTCT
     2041 GACGCTCAGT GGAACGAAAA CTCACGTTAA GGGATTTTGG TCATGATGAT AAACAATGTA
     2101 TGGTGCTAAT GTTGCTTCAA CAACAATTCT GTTGAACTGT GTTTTCATGT TTGCCAACAA
     2161 GCACCTTTAT ACTCGGTGGC CTCCCCACCA CCAACTTTTT TGCACTGCAA AAAAACACGC
     2221 TTTTGCACGC GGGCCCATAC ATAGTACAAA CTCTACGTTT CGTAGACTAT TTTACATAAA
     2281 TAGTCTACAC CGTTGTATAC GCTCCAAATA CACTACCACA CATTGAACCT TTTTGCAGTG
     2341 CAAAAAAGTA CGTGTCGGCA GTCACGTAGG CCGGCCTTAT CGGGTCGCGT CCTGTCACGT
     2401 ACGAATCACA TTATCGGACC GGACGAGTGT TGTCTTATCG TGACAGGACG CCAGCTTCCT
     2461 GTGTTGCTAA CCGCAGCCGG ACGCAACTCC TTATCGGAAC AGGACGCGCC TCCATATCAG
     2521 CCGCGCGTTA TCTCATGCGC GTGACCGGAC ACGAGGCGCC CGTCCCGCTT ATCGCGCCTA
     2581 TAAATACAGC CCGCAACGAT CTGGTAAACA CAGTTGAACA GCATCTGTTC GAATTAATTC
     2641 GGATCTCTGC AGCACGTGTT GACAATTAAT CATCGGCATA GTATATCGGC ATAGTATAAT
     2701 ACGACTCACT ATAGGAGGGC CACCATGGCC AAGTTGACCA GTGCCGTTCC GGTGCTCACC
     2761 GCGCGCGACG TCGCCGGAGC GGTCGAGTTC TGGACCGACC GGCTCGGGTT CTCCCGGGAC
     2821 TTCGTGGAGG ACGACTTCGC CGGTGTGGTC CGGGACGACG TGACCCTGTT CATCAGCGCG
     2881 GTCCAGGACC AGGTGGTGCC GGACAACACC CTGGCCTGGG TGTGGGTGCG CGGCCTGGAC
     2941 GAGCTGTACG CCGAGTGGTC GGAGGTCGTG TCCACGAACT TCCGGGACGC CTCCGGGCCG
     3001 GCCATGACCG AGATCGGCGA GCAGCCGTGG GGGCGGGAGT TCGCCCTGCG CGACCCGGCC
     3061 GGCAACTGCG TGCACTTCGT GGCCGAGGAG CAGGACTGA
//
```

Supplementary Data 11. The full-length sequence of the donor vector, pCR4-Pv.00443#1μH-P2A-BbsI.

```
LOCUS       pCR4-Pv.00443#1uH-P2A-BbsI_sequence      4169 bp    DNA     circular UNA 18-6-2018
DEFINITION
FEATURES             Location/Qualifiers
     primer          207..225
                     /label="M13_reverse_primer"
     primer          242..261
                     /label="T3 priming site"
     misc_feature    295..317
                     /feature_type="Zinc finger"
                     /label="Pv.00443-gRNA#1"
     microhomology   318..357
                     /label="Left microhomology of Pv.00443"
     Site            361..426
                     /site_type="restriction site"
                     /label="Linker + DmP2A"
     Site            complement(429..434)
                     /site_type="restriction site"
                     /label="BbsI recognition site"
     Site            437..442
                     /site_type="restriction site"
                     /label="BbsI recognition site"
     microhomology   445..484
                     /label="Right microhomology of Pv.00443"
     misc_feature    complement(485..507)
                     /feature_type="Zinc finger"
                     /label="Pv.00443-gRNA#1"
     primer          complement(542..560)
                     /label="T7_promoter"
     primer          complement(567..583)
                     /label="M13_forward20_primer"
     CDS             1375..2163
                     /cds_type="ORF"
                     /label="KanR/NeoR"
     CDS             complement(2416..3276)
                     /cds_type="ORF"
                     /label="Amp"
     rep_origin      3377..4020
                     /label="pUC origin"
ORIGIN
        1 AGCGCCCAAT ACGCAAACCG CCTCTCCCCG CGCGTTGGCC GATTCATTAA TGCAGCTGGC
       61 ACGACAGGTT TCCCGACTGG AAAGCGGGCA GTGAGCGCAA CGCAATTAAT GTGAGTTAGC
      121 TCACTCATTA GGCACCCCAG GCTTTACACT TTATGCTTCC GGCTCGTATG TTGTGTGGAA
      181 TTGTGAGCGG ATAACAATTT CACACAGGAA ACAGCTATGA CCATGATTAC GCCAAGCTCA
      241 GAATTAACCC TCACTAAAGG GACTAGTCCT GCAGGTTTAA ACGAATTCGC CCTTTTATAA
      301 GATTTTAATT ATGATGGTCA TCATCATAGA GTAGGCAGAA AACATCATTG CCATCATAAT
      361 GGTTCCGGTG CCACAAACTT CAGTTTGTTA AAGCAGGCCG GAGACGTCGA AGAGAACCCC
      421 GGACCGGGGT CTTCGAGAAG ACCTTAAAAT CTTATAACAA CATCCTAAAG TATTATAACA
      481 CCAACCATCA TAATTAAAAT CTTATAAAAG GGCGAATTCG CGGCCGCTAA ATTCAATTCG
      541 CCCTATAGTG AGTCGTATTA CAATTCACTG GCCGTCGTTT TACAACGTCG TGACTGGGAA
      601 AACCCTGGCG TTACCCAACT TAATCGCCTT GCAGCACATC CCCCTTTCGC CAGCTGGCGT
      661 AATAGCGAAG AGGCCCGCAC CGATCGCCCT TCCCAACAGT TGCGCAGCCT ATACGTACGG
      721 CAGTTTAAGG TTTACACCTA TAAAAGAGAG AGCCGTTATC GTCTGTTTGT GGATGTACAG
      781 AGTGATATTA TTGACACGCC GGGGCGACGG ATGGTGATCC CCCTGGCCAG TGCACGTCTG
      841 CTGTCAGATA AAGTCTCCCG TGAACTTTAC CCGGTGGTGC ATATCGGGGA TGAAAGCTGG
      901 CGCATGATGA CCACCGATAT GGCCAGTGTG CCGGTCTCCG TTATCGGGGA AGAAGTGGCT
      961 GATCTCAGCC ACCGCGAAAA TGACATCAAA AACGCCATTA ACCTGATGTT CTGGGGAATA
     1021 TAAATGTCAG GCATGAGATT ATCAAAAAGG ATCTTCACCT AGATCCTTTT CACGTAGAAA
     1081 GCCAGTCCGC AGAAACGGTG CTGACCCCGG ATGAATGTCA GCTACTGGGC TATCTGGACA
     1141 AGGGAAAACG CAAGCGCAAA GAGAAAGCAG GTAGCTTGCA GTGGGCTTAC ATGGCGATAG
     1201 CTAGACTGGG CGGTTTTATG GACAGCAAGC GAACCGGAAT TGCCAGCTGG GGCGCCCTCT
     1261 GGTAAGGTTG GGAAGCCCTG CAAAGTAAAC TGGATGGCTT TCTTGCCGCC AAGGATCTGA
     1321 TGGCGCAGGG GATCAAGCTC TGATCAAGAG ACAGGATGAG GATCGTTTCG CATGATTGAA
     1381 CAAGATGGAT TGCACGCAGG TTCTCCGGCC GCTTGGGTGG AGAGGCTATT CGGCTATGAC
     1441 TGGGCACAAC AGACAATCGG CTGCTCTGAT GCCGCCGTGT TCCGGCTGTC AGCGCAGGGG
     1501 CGCCCGGTTC TTTTTGTCAA GACCGACCTG TCCGGTGCCC TGAATGAACT GCAAGACGAG
     1561 GCAGCGCGGC TATCGTGGCT GGCCACGACG GGCGTTCCTT GCGCAGCTGT GCTCGACGTT
     1621 GTCACTGAAG CGGGAAGGGA CTGGCTGCTA TTGGGCGAAG TGCCGGGGCA GGATCTCCTG
     1681 TCATCTCACC TTGCTCCTGC CGAGAAAGTA TCCATCATGG CTGATGCAAT GCGGCGGCTG
     1741 CATACGCTTG ATCCGGCTAC CTGCCCATTC GACCACCAAG CGAAACATCG CATCGAGCGA
     1801 GCACGTACTC GGATGGAAGC CGGTCTTGTC GATCAGGATG ATCTGGACGA AGAGCATCAG
     1861 GGGCTCGCGC CAGCCGAACT GTTCGCCAGG CTCAAGGCGA GCATGCCCGA CGGCGAGGAT
     1921 CTCGTCGTGA CCCATGGCGA TGCCTGCTTG CCGAATATCA TGGTGGAAAA TGGCCGCTTT
     1981 TCTGGATTCA TCGACTGTGG CCGGCTGGGT GTGGCGGACC GCTATCAGGA CATAGCGTTG
     2041 GCTACCCGTG ATATTGCTGA AGAGCTTGGC GGCGAATGGG CTGACCGCTT CCTCGTGCTT
     2101 TACGGTATCG CCGCTCCCGA TTCGCAGCGC ATCGCCTTCT ATCGCCTTCT TGACGAGTTC
     2161 TTCTGAATTA TTAACGCTTA CAATTTCCTG ATGCGGTATT TTCTCCTTAC GCATCTGTGC
     2221 GGTATTTCAC ACCGCATCAG GTGGCACTTT TCGGGGAAAT GTGCGCGGAA CCCCTATTTG
     2281 TTTATTTTTC TAAATACATT CAAATATGTA TCCGCTCATG AGATTATCAA AAAGGATCTT
     2341 CACCTAGATC CTTTTAAATT AAAAATGAAG TTTTAAATCA ATCTAAAGTA TATATGAGTA
     2401 AACTTGGTCT GACAGTTACC AATGCTTAAT CAGTGAGGCA CCTATCTCAG CGATCTGTCT
     2461 ATTTCGTTCA TCCATAGTTG CCTGACTCCC CGTCGTGTAG ATAACTACGA TACGGGAGGG
     2521 CTTACCATCT GGCCCCAGTG CTGCAATGAT ACCGCGAGAC CCACGCTCAC CGGCTCCAGA
     2581 TTTATCAGCA ATAAACCAGC CAGCCGGAAG GGCCGAGCGC AGAAGTGGTC CTGCAACTTT
     2641 ATCCGCCTCC ATCCAGTCTA TTAATTGTTG CCGGGAAGCT AGAGTAAGTA GTTCGCCAGT
     2701 TAATAGTTTG CGCAACGTTG TTGCCATTGC TACAGGCATC GTGGTGTCAC GCTCGTCGTT
     2761 TGGTATGGCT TCATTCAGCT CCGGTTCCCA ACGATCAAGG CGAGTTACAT GATCCCCCAT
     2821 GTTGTGCAAA AAAGCGGTTA GCTCCTTCGG TCCTCCGATC GTTGTCAGAA GTAAGTTGGC
     2881 CGCAGTGTTA TCACTCATGG TTATGGCAGC ACTGCATAAT TCTCTTACTG TCATGCCATC
     2941 CGTAAGATGC TTTTCTGTGA CTGGTGAGTA CTCAACCAAG TCATTCTGAG AATAGTGTAT
     3001 GCGGCGACCG AGTTGCTCTT GCCCGGCGTC AATACGGGAT AATACCGCGC CACATAGCAG
     3061 AACTTTAAAA GTGCTCATCA TTGGAAAACG TTCTTCGGGG CGAAAACTCT CAAGGATCTT
     3121 ACCGCTGTTG AGATCCAGTT CGATGTAACC CACTCGTGCA CCCAACTGAT CTTCAGCATC
     3181 TTTTACTTTC ACCAGCGTTT CTGGGTGAGC AAAAACAGGA AGGCAAAATG CCGCAAAAAA
     3241 GGGAATAAGG GCGACACGGA AATGTTGAAT ACTCATACTC TTCCTTTTTC AATATTATTG
     3301 AAGCATTTAT CAGGGTTATT GTCTCATGAC CAAAATCCCT TAACGTGAGT TTTCGTTCCA
     3361 CTGAGCGTCA GACCCCGTAG AAAAGATCAA AGGATCTTCT TGAGATCCTT TTTTTCTGCG
     3421 CGTAATCTGC TGCTTGCAAA CAAAAAAACC ACCGCTACCA GCGGTGGTTT GTTTGCCGGA
     3481 TCAAGAGCTA CCAACTCTTT TTCCGAAGGT AACTGGCTTC AGCAGAGCGC AGATACCAAA
     3541 TACTGTTCTT CTAGTGTAGC CGTAGTTAGG CCACCACTTC AAGAACTCTG TAGCACCGCC
     3601 TACATACCTC GCTCTGCTAA TCCTGTTACC AGTGGCTGCT GCCAGTGGCG ATAAGTCGTG
     3661 TCTTACCGGG TTGGACTCAA GACGATAGTT ACCGGATAAG GCGCAGCGGT CGGGCTGAAC
     3721 GGGGGGTTCG TGCACACAGC CCAGCTTGGA GCGAACGACC TACACCGAAC TGAGATACCT
     3781 ACAGCGTGAG CTATGAGAAA GCGCCACGCT TCCCGAAGGG AGAAAGGCGG ACAGGTATCC
     3841 GGTAAGCGGC AGGGTCGGAA CAGGAGAGCG CACGAGGGAG CTTCCAGGGG GAAACGCCTG
     3901 GTATCTTTAT AGTCCTGTCG GGTTTCGCCA CCTCTGACTT GAGCGTCGAT TTTTGTGATG
     3961 CTCGTCAGGG GGGCGGAGCC TATGGAAAAA CGCCAGCAAC GCGGCCTTTT TACGGTTCCT
     4021 GGCCTTTTGC TGGCCTTTTG CTCACATGTT CTTTCCTGCG TTATCCCCTG ATTCTGTGGA
     4081 TAACCGTATT ACCGCCTTTG AGTGAGCTGA TACCGCTCGC CGCAGCCGAA CGACCGAGCG
     4141 CAGCGAGTCA GTGAGCGAGG AAGCGGAAG
//
```

Supplementary Data 12. The full-length sequence of the donor vector, pCR4-Pv.00443#1μH-P2A-BlaR.

```
LOCUS       pCR4-Pv.00443#1uH-P2A-HaloTag_sequence      5042 bp    DNA     circular UNA 18-6-2018
DEFINITION
FEATURES             Location/Qualifiers
     primer          207..225
                     /label="M13_reverse_primer"
     primer          242..261
                     /label="T3 priming site"
     misc_feature    295..317
                     /feature_type="Zinc finger"
                     /label="Pv.00443-gRNA#1"
     microhomology   318..357
                     /label="Left microhomology of Pv.00443"
     Site            361..426
                     /site_type="restriction site"
                     /label="Linker + DmP2A"
     CDS             427..1317
                     /cds_type="ORF"
                     /label="HaloTag (stop less)"
     microhomology   1318..1357
                     /label="Right microhomology of Pv.00443"
     misc_feature    complement(1358..1380)
                     /feature_type="Zinc finger"
                     /label="Pv.00443-gRNA#1"
     primer          complement(1415..1433)
                     /label="T7_promoter"
     primer          complement(1440..1456)
                     /label="M13_forward20_primer"
     CDS             2248..3036
                     /cds_type="ORF"
                     /label="KanR/NeoR"
     CDS             complement(3289..4149)
                     /cds_type="ORF"
                     /label="Amp"
     rep_origin      4250..4893
                     /label="pUC origin"
ORIGIN
        1 AGCGCCCAAT ACGCAAACCG CCTCTCCCCG CGCGTTGGCC GATTCATTAA TGCAGCTGGC
       61 ACGACAGGTT TCCCGACTGG AAAGCGGGCA GTGAGCGCAA CGCAATTAAT GTGAGTTAGC
      121 TCACTCATTA GGCACCCCAG GCTTTACACT TTATGCTTCC GGCTCGTATG TTGTGTGGAA
      181 TTGTGAGCGG ATAACAATTT CACACAGGAA ACAGCTATGA CCATGATTAC GCCAAGCTCA
      241 GAATTAACCC TCACTAAAGG GACTAGTCCT GCAGGTTTAA ACGAATTCGC CCTTTTATAA
      301 GATTTTAATT ATGATGGTCA TCATCATAGA GTAGGCAGAA AACATCATTG CCATCATAAT
      361 GGTTCCGGTG CCACAAACTT CAGTTTGTTA AAGCAGGCCG GAGACGTCGA AGAGAACCCC
      421 GGACCGATGG CAGAAATCGG TACTGGCTTT CCATTCGACC CCCATTATGT GGAAGTCCTG
      481 GGCGAGCGCA TGCACTACGT CGATGTTGGT CCGCGCGATG GCACCCCTGT GCTGTTCCTG
      541 CACGGTAACC CGACCTCCTC CTACGTGTGG CGCAACATCA TCCCGCATGT TGCACCGACC
      601 CATCGCTGCA TTGCTCCAGA CCTGATCGGT ATGGGCAAAT CCGACAAACC AGACCTGGGT
      661 TATTTCTTCG ACGACCACGT CCGCTTCATG GATGCCTTCA TCGAAGCCCT GGGTCTGGAA
      721 GAGGTCGTCC TGGTCATTCA CGACTGGGGC TCCGCTCTGG GTTTCCACTG GGCCAAGCGC
      781 AATCCAGAGC GCGTCAAAGG TATTGCATTT ATGGAGTTCA TCCGCCCTAT CCCGACCTGG
      841 GACGAATGGC CAGAATTTGC CCGCGAGACC TTCCAGGCCT TCCGCACCAC CGACGTCGGC
      901 CGCAAGCTGA TCATCGATCA GAACGTTTTT ATCGAGGGTA CGCTGCCGAT GGGTGTCGTC
      961 CGCCCGCTGA CTGAAGTCGA GATGGACCAT TACCGCGAGC CGTTCCTGAA TCCTGTTGAC
     1021 CGCGAGCCAC TGTGGCGCTT CCCAAACGAG CTGCCAATCG CCGGTGAGCC AGCGAACATC
     1081 GTCGCGCTGG TCGAAGAATA CATGGACTGG CTGCACCAGT CCCCTGTCCC GAAGCTGCTG
     1141 TTCTGGGGCA CCCCAGGCGT TCTGATCCCA CCGGCCGAAG CCGCTCGCCT GGCCAAAAGC
     1201 CTGCCTAACT GCAAGGCTGT GGACATCGGC CCGGGTCTGA ATCTGCTGCA AGAAGACAAC
     1261 CCGGACCTGA TCGGCAGCGA GATCGCGCGC TGGCTGTCGA CGCTCGAGAT TTCCGGCTAA
     1321 AATCTTATAA CAACATCCTA AAGTATTATA ACACCAACCA TCATAATTAA AATCTTATAA
     1381 AAGGGCGAAT TCGCGGCCGC TAAATTCAAT TCGCCCTATA GTGAGTCGTA TTACAATTCA
     1441 CTGGCCGTCG TTTTACAACG TCGTGACTGG GAAAACCCTG GCGTTACCCA ACTTAATCGC
     1501 CTTGCAGCAC ATCCCCCTTT CGCCAGCTGG CGTAATAGCG AAGAGGCCCG CACCGATCGC
     1561 CCTTCCCAAC AGTTGCGCAG CCTATACGTA CGGCAGTTTA AGGTTTACAC CTATAAAAGA
     1621 GAGAGCCGTT ATCGTCTGTT TGTGGATGTA CAGAGTGATA TTATTGACAC GCCGGGGCGA
     1681 CGGATGGTGA TCCCCCTGGC CAGTGCACGT CTGCTGTCAG ATAAAGTCTC CCGTGAACTT
     1741 TACCCGGTGG TGCATATCGG GGATGAAAGC TGGCGCATGA TGACCACCGA TATGGCCAGT
     1801 GTGCCGGTCT CCGTTATCGG GGAAGAAGTG GCTGATCTCA GCCACCGCGA AAATGACATC
     1861 AAAAACGCCA TTAACCTGAT GTTCTGGGGA ATATAAATGT CAGGCATGAG ATTATCAAAA
     1921 AGGATCTTCA CCTAGATCCT TTTCACGTAG AAAGCCAGTC CGCAGAAACG GTGCTGACCC
     1981 CGGATGAATG TCAGCTACTG GGCTATCTGG ACAAGGGAAA ACGCAAGCGC AAAGAGAAAG
     2041 CAGGTAGCTT GCAGTGGGCT TACATGGCGA TAGCTAGACT GGGCGGTTTT ATGGACAGCA
     2101 AGCGAACCGG AATTGCCAGC TGGGGCGCCC TCTGGTAAGG TTGGGAAGCC CTGCAAAGTA
     2161 AACTGGATGG CTTTCTTGCC GCCAAGGATC TGATGGCGCA GGGGATCAAG CTCTGATCAA
     2221 GAGACAGGAT GAGGATCGTT TCGCATGATT GAACAAGATG GATTGCACGC AGGTTCTCCG
     2281 GCCGCTTGGG TGGAGAGGCT ATTCGGCTAT GACTGGGCAC AACAGACAAT CGGCTGCTCT
     2341 GATGCCGCCG TGTTCCGGCT GTCAGCGCAG GGGCGCCCGG TTCTTTTTGT CAAGACCGAC
     2401 CTGTCCGGTG CCCTGAATGA ACTGCAAGAC GAGGCAGCGC GGCTATCGTG GCTGGCCACG
     2461 ACGGGCGTTC CTTGCGCAGC TGTGCTCGAC GTTGTCACTG AAGCGGGAAG GGACTGGCTG
     2521 CTATTGGGCG AAGTGCCGGG GCAGGATCTC CTGTCATCTC ACCTTGCTCC TGCCGAGAAA
     2581 GTATCCATCA TGGCTGATGC AATGCGGCGG CTGCATACGC TTGATCCGGC TACCTGCCCA
     2641 TTCGACCACC AAGCGAAACA TCGCATCGAG CGAGCACGTA CTCGGATGGA AGCCGGTCTT
     2701 GTCGATCAGG ATGATCTGGA CGAAGAGCAT CAGGGGCTCG CGCCAGCCGA ACTGTTCGCC
     2761 AGGCTCAAGG CGAGCATGCC CGACGGCGAG GATCTCGTCG TGACCCATGG CGATGCCTGC
     2821 TTGCCGAATA TCATGGTGGA AAATGGCCGC TTTTCTGGAT TCATCGACTG TGGCCGGCTG
     2881 GGTGTGGCGG ACCGCTATCA GGACATAGCG TTGGCTACCC GTGATATTGC TGAAGAGCTT
     2941 GGCGGCGAAT GGGCTGACCG CTTCCTCGTG CTTTACGGTA TCGCCGCTCC CGATTCGCAG
     3001 CGCATCGCCT TCTATCGCCT TCTTGACGAG TTCTTCTGAA TTATTAACGC TTACAATTTC
     3061 CTGATGCGGT ATTTTCTCCT TACGCATCTG TGCGGTATTT CACACCGCAT CAGGTGGCAC
     3121 TTTTCGGGGA AATGTGCGCG GAACCCCTAT TTGTTTATTT TTCTAAATAC ATTCAAATAT
     3181 GTATCCGCTC ATGAGATTAT CAAAAAGGAT CTTCACCTAG ATCCTTTTAA ATTAAAAATG
     3241 AAGTTTTAAA TCAATCTAAA GTATATATGA GTAAACTTGG TCTGACAGTT ACCAATGCTT
     3301 AATCAGTGAG GCACCTATCT CAGCGATCTG TCTATTTCGT TCATCCATAG TTGCCTGACT
     3361 CCCCGTCGTG TAGATAACTA CGATACGGGA GGGCTTACCA TCTGGCCCCA GTGCTGCAAT
     3421 GATACCGCGA GACCCACGCT CACCGGCTCC AGATTTATCA GCAATAAACC AGCCAGCCGG
     3481 AAGGGCCGAG CGCAGAAGTG GTCCTGCAAC TTTATCCGCC TCCATCCAGT CTATTAATTG
     3541 TTGCCGGGAA GCTAGAGTAA GTAGTTCGCC AGTTAATAGT TTGCGCAACG TTGTTGCCAT
     3601 TGCTACAGGC ATCGTGGTGT CACGCTCGTC GTTTGGTATG GCTTCATTCA GCTCCGGTTC
     3661 CCAACGATCA AGGCGAGTTA CATGATCCCC CATGTTGTGC AAAAAAGCGG TTAGCTCCTT
     3721 CGGTCCTCCG ATCGTTGTCA GAAGTAAGTT GGCCGCAGTG TTATCACTCA TGGTTATGGC
     3781 AGCACTGCAT AATTCTCTTA CTGTCATGCC ATCCGTAAGA TGCTTTTCTG TGACTGGTGA
     3841 GTACTCAACC AAGTCATTCT GAGAATAGTG TATGCGGCGA CCGAGTTGCT CTTGCCCGGC
     3901 GTCAATACGG GATAATACCG CGCCACATAG CAGAACTTTA AAAGTGCTCA TCATTGGAAA
     3961 ACGTTCTTCG GGGCGAAAAC TCTCAAGGAT CTTACCGCTG TTGAGATCCA GTTCGATGTA
     4021 ACCCACTCGT GCACCCAACT GATCTTCAGC ATCTTTTACT TTCACCAGCG TTTCTGGGTG
     4081 AGCAAAAACA GGAAGGCAAA ATGCCGCAAA AAAGGGAATA AGGGCGACAC GGAAATGTTG
     4141 AATACTCATA CTCTTCCTTT TTCAATATTA TTGAAGCATT TATCAGGGTT ATTGTCTCAT
     4201 GACCAAAATC CCTTAACGTG AGTTTTCGTT CCACTGAGCG TCAGACCCCG TAGAAAAGAT
     4261 CAAAGGATCT TCTTGAGATC CTTTTTTTCT GCGCGTAATC TGCTGCTTGC AAACAAAAAA
     4321 ACCACCGCTA CCAGCGGTGG TTTGTTTGCC GGATCAAGAG CTACCAACTC TTTTTCCGAA
     4381 GGTAACTGGC TTCAGCAGAG CGCAGATACC AAATACTGTT CTTCTAGTGT AGCCGTAGTT
     4441 AGGCCACCAC TTCAAGAACT CTGTAGCACC GCCTACATAC CTCGCTCTGC TAATCCTGTT
     4501 ACCAGTGGCT GCTGCCAGTG GCGATAAGTC GTGTCTTACC GGGTTGGACT CAAGACGATA
     4561 GTTACCGGAT AAGGCGCAGC GGTCGGGCTG AACGGGGGGT TCGTGCACAC AGCCCAGCTT
     4621 GGAGCGAACG ACCTACACCG AACTGAGATA CCTACAGCGT GAGCTATGAG AAAGCGCCAC
     4681 GCTTCCCGAA GGGAGAAAGG CGGACAGGTA TCCGGTAAGC GGCAGGGTCG GAACAGGAGA
     4741 GCGCACGAGG GAGCTTCCAG GGGGAAACGC CTGGTATCTT TATAGTCCTG TCGGGTTTCG
     4801 CCACCTCTGA CTTGAGCGTC GATTTTTGTG ATGCTCGTCA GGGGGGCGGA GCCTATGGAA
     4861 AAACGCCAGC AACGCGGCCT TTTTACGGTT CCTGGCCTTT TGCTGGCCTT TTGCTCACAT
     4921 GTTCTTTCCT GCGTTATCCC CTGATTCTGT GGATAACCGT ATTACCGCCT TTGAGTGAGC
```

```
     4981 TGATACCGCT CGCCGCAGCC GAACGACCGA GCGCAGCGAG TCAGTGAGCG AGGAAGCGGA
     5041 AG
//
```

Supplementary Data 13. The full-length sequence of the donor vector, pCR4-Pv.00443#1µH-P2A-BlaR.

```
LOCUS       pCR4-Pv.00443#1uH-P2A-BlaR_sequence      4547 bp    DNA     circular UNA 18-6-2018
DEFINITION
FEATURES             Location/Qualifiers
     primer          207..225
                     /label="M13_reverse_primer"
     primer          242..261
                     /label="T3 priming site"
     misc_feature    295..317
                     /feature_type="Zinc finger"
                     /label="Pv.00443-gRNA#1"
     microhomology   318..357
                     /label="Left microhomology of Pv.00443"
     Site            361..426
                     /site_type="restriction site"
                     /label="Linker + DmP2A"
     CDS             427..822
                     /cds_type="ORF"
                     /label="Blasticidin (stop less)"
     microhomology   823..862
                     /label="Right microhomology of Pv.00443"
     misc_feature    complement(863..885)
                     /feature_type="Zinc finger"
                     /label="Pv.00443-gRNA#1"
     primer          complement(920..938)
                     /label="T7_promoter"
     primer          complement(945..961)
                     /label="M13_forward20_primer"
     CDS             1753..2541
                     /cds_type="ORF"
                     /label="KanR/NeoR"
     CDS             complement(2794..3654)
                     /cds_type="ORF"
                     /label="Amp"
     rep_origin      3755..4398
                     /label="pUC origin"
ORIGIN
        1 AGCGCCCAAT ACGCAAACCG CCTCTCCCCG CGCGTTGGCC GATTCATTAA TGCAGCTGGC
       61 ACGACAGGTT TCCCGACTGG AAAGCGGGCA GTGAGCGCAA CGCAATTAAT GTGAGTTAGC
      121 TCACTCATTA GGCACCCCAG GCTTTACACT TTATGCTTCC GGCTCGTATG TTGTGTGGAA
      181 TTGTGAGCGG ATAACAATTT CACACAGGAA ACAGCTATGA CCATGATTAC GCCAAGCTCA
      241 GAATTAACCC TCACTAAAGG GACTAGTCCT GCAGGTTTAA ACGAATTCGC CCTTTTATAA
      301 GATTTTAATT ATGATGGTCA TCATCATAGA GTAGGCAGAA AACATCATTG CCATCATAAT
      361 GGTTCCGGTG CCACAAACTT CAGTTTGTTA AAGCAGGCCG GAGACGTCGA AGAGAACCCC
      421 GGACCGATGG CCAAGCCTTT GTCTCAAGAA GAATCCACCC TCATTGAAAG AGCAACGGCT
      481 ACAATCAACA GCATCCCCAT CTCTGAAGAC TACAGCGTCG CCAGCGCAGC TCTCTCTAGC
      541 GACGGCCGCA TCTTCACTGG TGTCAATGTA TATCATTTTA CTGGGGGACC TTGCGCAGAA
      601 CTCGTGGTGC TGGGCACTGC TGCTGCTGCG GCAGCTGGCA ACCTGACTTG TATCGTCGCG
      661 ATCGGAAATG AGAACAGGGG CATCTTGAGc CCCTGCGGAC GGTGCCGACA GGTTCTTCTC
      721 GATCTGCATC CTGGGATCAA AGCCATAGTG AAGGACAGTG ATGGACAGCC GACGGCAGTT
      781 GGGATTCGTG AATTGCTGCC CTCTGGTTAT GTGTGGGAGG GCTAAAATCT TATAACAACA
      841 TCCTAAAGTA TTATAACACC AACCATCATA ATTAAAATCT TATAAAAGGG CGAATTCGCG
      901 GCCGCTAAAT TCAATTCGCC CTATAGTGAG TCGTATTACA ATTCACTGGC CGTCGTTTTA
      961 CAACGTCGTG ACTGGGAAAA CCCTGGCGTT ACCCAACTTA ATCGCCTTGC AGCACATCCC
     1021 CCTTTCGCCA GCTGGCGTAA TAGCGAAGAG GCCCGCACCG ATCGCCCTTC CCAACAGTTG
     1081 CGCAGCCTAT ACGTACGGCA GTTTAAGGTT TACACCTATA AAAGAGAGAG CCGTTATCGT
     1141 CTGTTTGTGG ATGTACAGAG TGATATTATT GACACGCCGG GGCGACGGAT GGTGATCCCC
     1201 CTGGCCAGTG CACGTCTGCT GTCAGATAAA GTCTCCCGTG AACTTTACCC GGTGGTGCAT
     1261 ATCGGGGATG AAAGCTGGCG CATGATGACC ACCGATATGG CCAGTGTGCC GGTCTCCGTT
     1321 ATCGGGGAAG AAGTGGCTGA TCTCAGCCAC CGCGAAAATG ACATCAAAAA CGCCATTAAC
     1381 CTGATGTTCT GGGGAATATA AATGTCAGGC ATGAGATTAT CAAAAAGGAT CTTCACCTAG
     1441 ATCCTTTTCA CGTAGAAAGC CAGTCCGCAG AAACGGTGCT GACCCCGGAT GAATGTCAGC
     1501 TACTGGGCTA TCTGGACAAG GGAAAACGCA AGCGCAAAGA GAAAGCAGGT AGCTTGCAGT
     1561 GGGCTTACAT GGCGATAGCT AGACTGGGCG GTTTTATGGA CAGCAAGCGA ACCGGAATTG
     1621 CCAGCTGGGG CGCCCTCTGG TAAGGTTGGG AAGCCCTGCA AAGTAAACTG GATGGCTTTC
     1681 TTGCCGCCAA GGATCTGATG GCGCAGGGGA TCAAGCTCTG ATCAAGAGAC AGGATGAGGA
     1741 TCGTTTCGCA TGATTGAACA AGATGGATTG CACGCAGGTT CTCCGGCCGC TTGGGTGGAG
     1801 AGGCTATTCG GCTATGACTG GGCACAACAG ACAATCGGCT GCTCTGATGC CGCCGTGTTC
     1861 CGGCTGTCAG CGCAGGGGCG CCCGGTTCTT TTTGTCAAGA CCGACCTGTC CGGTGCCCTG
     1921 AATGAACTGC AAGACGAGGC AGCGCGGCTA TCGTGGCTGG CCACGACGGG CGTTCCTTGC
     1981 GCAGCTGTGC TCGACGTTGT CACTGAAGCG GGAAGGGACT GGCTGCTATT GGGCGAAGTG
     2041 CCGGGGCAGG ATCTCCTGTC ATCTCACCTT GCTCCTGCCG AGAAAGTATC CATCATGGCT
     2101 GATGCAATGC GGCGGCTGCA TACGCTTGAT CCGGCTACCT GCCCATTCGA CCACCAAGCG
     2161 AAACATCGCA TCGAGCGAGC ACGTACTCGG ATGGAAGCCG GTCTTGTCGA TCAGGATGAT
     2221 CTGGACGAAG AGCATCAGGG GCTCGCGCCA GCCGAACTGT TCGCCAGGCT CAAGGCGAGC
     2281 ATGCCCGACG GCGAGGATCT CGTCGTGACC CATGGCGATG CCTGCTTGCC GAATATCATG
     2341 GTGGAAAATG GCCGCTTTTC TGGATTCATC GACTGTGGCC GGCTGGGTGT GGCGGACCGC
     2401 TATCAGGACA TAGCGTTGGC TACCCGTGAT ATTGCTGAAG AGCTTGGCGG CGAATGGGCT
     2461 GACCGCTTCC TCGTGCTTTA CGGTATCGCC GCTCCCGATT CGCAGCGCAT CGCCTTCTAT
     2521 CGCCTTCTTG ACGAGTTCTT CTGAATTATT AACGCTTACA ATTTCCTGAT GCGGTATTTT
     2581 CTCCTTACGC ATCTGTGCGG TATTTCACAC CGCATCAGGT GGCACTTTTC GGGGAAATGT
     2641 GCGCGGAACC CCTATTTGTT TATTTTTCTA AATACATTCA AATATGTATC CGCTCATGAG
     2701 ATTATCAAAA AGGATCTTCA CCTAGATCCT TTTAAATTAA AAATGAAGTT TTAAATCAAT
     2761 CTAAAGTATA TATGAGTAAA CTTGGTCTGA CAGTTACCAA TGCTTAATCA GTGAGGCACC
     2821 TATCTCAGCG ATCTGTCTAT TTCGTTCATC CATAGTTGCC TGACTCCCCG TCGTGTAGAT
     2881 AACTACGATA CGGGAGGGCT TACCATCTGG CCCCAGTGCT GCAATGATAC CGCGAGACCC
     2941 ACGCTCACCG GCTCCAGATT TATCAGCAAT AAACCAGCCA GCCGGAAGGG CCGAGCGCAG
     3001 AAGTGGTCCT GCAACTTTAT CCGCCTCCAT CCAGTCTATT AATTGTTGCC GGGAAGCTAG
     3061 AGTAAGTAGT TCGCCAGTTA ATAGTTTGCG CAACGTTGTT GCCATTGCTA CAGGCATCGT
     3121 GGTGTCACGC TCGTCGTTTG GTATGGCTTC ATTCAGCTCC GGTTCCCAAC GATCAAGGCG
     3181 AGTTACATGA TCCCCCATGT TGTGCAAAAA AGCGGTTAGC TCCTTCGGTC CTCCGATCGT
     3241 TGTCAGAAGT AAGTTGGCCG CAGTGTTATC ACTCATGGTT ATGGCAGCAC TGCATAATTC
     3301 TCTTACTGTC ATGCCATCCG TAAGATGCTT TTCTGTGACT GGTGAGTACT CAACCAAGTC
     3361 ATTCTGAGAA TAGTGTATGC GGCGACCGAG TTGCTCTTGC CCGGCGTCAA TACGGGATAA
     3421 TACCGCGCCA CATAGCAGAA CTTTAAAAGT GCTCATCATT GGAAAACGTT CTTCGGGGCG
     3481 AAAACTCTCA AGGATCTTAC CGCTGTTGAG ATCCAGTTCG ATGTAACCCA CTCGTGCACC
     3541 CAACTGATCT TCAGCATCTT TTACTTTCAC CAGCGTTTCT GGGTGAGCAA AAACAGGAAG
     3601 GCAAAATGCC GCAAAAAAGG GAATAAGGGC GACACGGAAA TGTTGAATAC TCATACTCTT
     3661 CCTTTTTCAA TATTATTGAA GCATTTATCA GGGTTATTGT CTCATGACCA AAATCCCTTA
     3721 ACGTGAGTTT TCGTTCCACT GAGCGTCAGA CCCCGTAGAA AAGATCAAAG GATCTTCTTG
     3781 AGATCCTTTT TTTCTGCGCG TAATCTGCTG CTTGCAAACA AAAAAACCAC CGCTACCAGC
     3841 GGTGGTTTGT TTGCCGGATC AAGAGCTACC AACTCTTTTT CCGAAGGTAA CTGGCTTCAG
     3901 CAGAGCGCAG ATACCAAATA CTGTTCTTCT AGTGTAGCCG TAGTTAGGCC ACCACTTCAA
     3961 GAACTCTGTA GCACCGCCTA CATACCTCGC TCTGCTAATC CTGTTACCAG TGGCTGCTGC
     4021 CAGTGGCGAT AAGTCGTGTC TTACCGGGTT GGACTCAAGA CGATAGTTAC CGGATAAGGC
     4081 GCAGCGGTCG GGCTGAACGG GGGGTTCGTG CACACAGCCC AGCTTGGAGC GAACGACCTA
     4141 CACCGAACTG AGATACCTAC AGCGTGAGCT ATGAGAAAGC GCCACGCTTC CCGAAGGGAG
     4201 AAAGGCGGAC AGGTATCCGG TAAGCGGCAG GGTCGGAACA GGAGAGCGCA CGAGGGAGCT
     4261 TCCAGGGGGA AACGCCTGGT ATCTTTATAG TCCTGTCGGG TTTCGCCACC TCTGACTTGA
     4321 GCGTCGATTT TTGTGATGCT CGTCAGGGGG GCGGAGCCTA TGGAAAAACG CCAGCAACGC
     4381 GGCCTTTTTA CGGTTCCTGG CCTTTTGCTG GCCTTTTGCT CACATGTTCT TTCCTGCGTT
     4441 ATCCCCTGAT TCTGTGGATA ACCGTATTAC CGCCTTTGAG TGAGCTGATA CCGCTCGCCG
     4501 CAGCCGAACG ACCGAGCGCA GCGAGTCAGT GAGCGAGGAA GCGGAAG
//
```

Supplementary Data 14. The full-length sequence of the donor vector, pCR4-Pv.00443#1μH-P2A-GCaMP3.

```
LOCUS       pCR4-Pv.00443#1uH-P2A-GCaMP3_sequence      5501 bp    DNA     circular UNA 18-6-2018
DEFINITION
FEATURES             Location/Qualifiers
     primer          207..225
                     /label="M13_reverse_primer"
     primer          242..261
                     /label="T3 priming site"
     misc_feature    295..317
                     /feature_type="Zinc finger"
                     /label="Pv.00443-gRNA#1"
     microhomology   318..357
                     /label="Left microhomology of Pv.00443"
     Site            361..426
                     /site_type="restriction site"
                     /label="Linker + DmP2A"
     CDS             427..1776
                     /cds_type="ORF"
                     /label="GCaMP3"
     CDS             436..453
                     /label="6xHis"
     CDS             457..489
                     /label="T7 tag (gene 10 leader)"
     CDS             493..516
                     /label="Xpress(TM) tag"
     CDS             544..600
                     /label="calmodulin-binding peptide"
     microhomology   1777..1816
                     /label="Right microhomology of Pv.00443"
     misc_feature    complement(1817..1839)
                     /feature_type="Zinc finger"
                     /label="Pv.00443-gRNA#1"
     primer          complement(1874..1892)
                     /label="T7_promoter"
     primer          complement(1899..1915)
                     /label="M13_forward20_primer"
     CDS             2707..3495
                     /cds_type="ORF"
                     /label="KanR/NeoR"
     CDS             complement(3748..4608)
                     /cds_type="ORF"
                     /label="Amp"
     rep_origin      4709..5352
                     /label="pUC origin"
ORIGIN
        1 AGCGCCCAAT ACGCAAACCG CCTCTCCCCG CGCGTTGGCC GATTCATTAA TGCAGCTGGC
       61 ACGACAGGTT TCCCGACTGG AAAGCGGGCA GTGAGCGCAA CGCAATTAAT GTGAGTTAGC
      121 TCACTCATTA GGCACCCCAG GCTTTACACT TTATGCTTCC GGCTCGTATG TTGTGTGGAA
      181 TTGTGAGCGG ATAACAATTT CACACAGGAA ACAGCTATGA CCATGATTAC GCCAAGCTCA
      241 GAATTAACCC TCACTAAAGG GACTAGTCCT GCAGGTTTAA ACGAATTCGC CCTTTTATAA
      301 GATTTTAATT ATGATGGTCA TCATCATAGA GTAGGCAGAA AACATCATTG CCATCATAAT
      361 GGTTCCGGTG CCACAAACTT CAGTTTGTTA AAGCAGGCCG GAGACGTCGA AGAGAACCCC
      421 GGACCGATGG GTTCTCATCA TCATCATCAT CATGGTATGG CTAGCATGAC TGGTGGACAG
      481 CAAATGGGTC GGGATCTGTA CGACGATGAC GATAAGGATC TCGCCACCAT GGTCGACTCA
      541 TCACGTCGTA AGTGGAATAA GACAGGTCAC GCAGTCAGAG CTATAGGTCG GCTGAGCTCA
      601 CTCGAGAACG TCTATATCAA GGCCGACAAG CAGAAGAACG GCATCAAGGC GAACTTCAAG
      661 ATCCGCCACA ACATCGAGGA CGGCGGCGTG CAGCTCGCCT ACCACTACCA GCAGAACACC
      721 CCCATCGGCG ACGGCCCCGT GCTGCTGCCC GACAACCACT ACCTGAGCGT GCAGTCCAAA
      781 CTTTCGAAAG ACCCCAACGA GAAGCGCGAT CACATGGTCC TGCTGGAGTT CGTGACCGCC
      841 GCCGGGATCA CTCTCGGCAT GGACGAGCTG TACAAGGGCG GTACCGGAGG GAGCATGGTG
      901 AGCAAGGGCG AGGAGCTGTT CACCGGGGTG GTGCCCATCC TGGTCGAGCT GGACGGCGAC
      961 GTAAACGGCC ACAAGTTCAG CGTGTCCGGC GAGGGCGAGG GCGATGCCAC CTACGGCAAG
     1021 CTGACCCTGA AGTTCATCTG CACCACCGGC AAGCTGCCCG TGCCCTGGCC CACCCTCGTG
     1081 ACCACCCTGA CCTACGGCGT GCAGTGCTTC AGCCGCTACC CCGACCACAT GAAGCAGCAC
     1141 GACTTCTTCA AGTCCGCCAT GCCCGAAGGC TACATCCAGG AGCGCACCAT CTTCTTCAAG
     1201 GACGACGGCA ACTACAAGAC CCGCGCCGAG GTGAAGTTCG AGGGCGACAC CCTGGTGAAC
     1261 CGCATCGAGC TGAAGGGCAT CGACTTCAAG GAGGACGGCA ACATCCTGGG GCACAAGCTG
     1321 GAGTACAACA CGCGTGACCA ACTGACTGAA GAGCAGATCG CAGAATTTAA AGAGGCTTTC
     1381 TCCCTATTTG ACAAGGACGG GGATGGGACA ATAACAACCA AGGAGCTGGG GACGGTGATG
     1441 CGGTCTCTGG GGCAGAACCC CACAGAAGCA GAGCTGCAGG ACATGATCAA TGAAGTAGAT
     1501 GCCGACGGTG ACGGCACAAT CGACTTCCCT GAGTTCCTGA CAATGATGGC AAGAAAAATG
     1561 AAAGACACAG ACAGTGAAGA AGAAATTAGA GAAGCGTTCC GTGTGTTTGA TAAGGATGGC
     1621 AATGGCTACA TCAGTGCAGC AGAGCTTCGC CACGTGATGA CAAACCTTGG AGAGAAGTTA
     1681 ACAGATGAAG AGGTTGATGA AATGATCAGG GAAGCAGACA TCGATGGGGA TGGTCAGGTA
     1741 AACTACGAAG AGTTTGTACA AATGATGACA GCGAAGTAAA ATCTTATAAC AACATCCTAA
     1801 AGTATTATAA CACCAACCAT CATAATTAAA ATCTTATAAA AGGGCGAATT CGCGGCCGCT
     1861 AAATTCAATT CGCCCTATAG TGAGTCGTAT TACAATTCAC TGGCCGTCGT TTTACAACGT
     1921 CGTGACTGGG AAAACCCTGG CGTTACCCAA CTTAATCGCC TTGCAGCACA TCCCCCTTTC
     1981 GCCAGCTGGC GTAATAGCGA AGAGGCCCGC ACCGATCGCC CTTCCCAACA GTTGCGCAGC
     2041 CTATACGTAC GGCAGTTTAA GGTTTACACC TATAAAAGAG AGAGCCGTTA TCGTCTGTTT
     2101 GTGGATGTAC AGAGTGATAT TATTGACACG CCGGGGCGAC GGATGGTGAT CCCCCTGGCC
     2161 AGTGCACGTC TGCTGTCAGA TAAAGTCTCC CGTGAACTTT ACCCGGTGGT GCATATCGGG
     2221 GATGAAAGCT GGCGCATGAT GACCACCGAT ATGGCCAGTG TGCCGGTCTC CGTTATCGGG
     2281 GAAGAAGTGG CTGATCTCAG CCACCGCGAA AATGACATCA AAAACGCCAT TAACCTGATG
     2341 TTCTGGGGAA TATAAATGTC AGGCATGAGA TTATCAAAAA GGATCTTCAC CTAGATCCTT
     2401 TTCACGTAGA AAGCCAGTCC GCAGAAACGG TGCTGACCCC GGATGAATGT CAGCTACTGG
     2461 GCTATCTGGA CAAGGGAAAA CGCAAGCGCA AAGAGAAAGC AGGTAGCTTG CAGTGGGCTT
     2521 ACATGGCGAT AGCTAGACTG GGCGGTTTTA TGGACAGCAA GCGAACCGGA ATTGCCAGCT
     2581 GGGGCGCCCT CTGGTAAGGT TGGGAAGCCC TGCAAAGTAA ACTGGATGGC TTTCTTGCCG
     2641 CCAAGGATCT GATGGCGCAG GGGATCAAGC TCTGATCAAG AGACAGGATG AGGATCGTTT
     2701 CGCATGATTG AACAAGATGG ATTGCACGCA GGTTCTCCGG CCGCTTGGGT GGAGAGGCTA
     2761 TTCGGCTATG ACTGGGCACA ACAGACAATC GGCTGCTCTG ATGCCGCCGT GTTCCGGCTG
     2821 TCAGCGCAGG GGCGCCCGGT TCTTTTTGTC AAGACCGACC TGTCCGGTGC CCTGAATGAA
     2881 CTGCAAGACG AGGCAGCGCG GCTATCGTGG CTGGCCACGA CGGGCGTTCC TTGCGCAGCT
     2941 GTGCTCGACG TTGTCACTGA AGCGGGAAGG GACTGGCTGC TATTGGGCGA AGTGCCGGGG
     3001 CAGGATCTCC TGTCATCTCA CCTTGCTCCT GCCGAGAAAG TATCCATCAT GGCTGATGCA
     3061 ATGCGGCGGC TGCATACGCT TGATCCGGCT ACCTGCCCAT TCGACCACCA AGCGAAACAT
     3121 CGCATCGAGC GAGCACGTAC TCGGATGGAA GCCGGTCTTG TCGATCAGGA TGATCTGGAC
     3181 GAAGAGCATC AGGGGCTCGC GCCAGCCGAA CTGTTCGCCA GGCTCAAGGC GAGCATGCCC
     3241 GACGGCGAGG ATCTCGTCGT GACCCATGGC GATGCCTGCT TGCCGAATAT CATGGTGGAA
     3301 AATGGCCGCT TTTCTGGATT CATCGACTGT GGCCGGCTGG GTGTGGCGGA CCGCTATCAG
     3361 GACATAGCGT TGGCTACCCG TGATATTGCT GAAGAGCTTG GCGGCGAATG GGCTGACCGC
     3421 TTCCTCGTGC TTTACGGTAT CGCCGCTCCC GATTCGCAGC GCATCGCCTT CTATCGCCTT
     3481 CTTGACGAGT TCTTCTGAAT TATTAACGCT TACAATTTCC TGATGCGGTA TTTTCTCCTT
     3541 ACGCATCTGT GCGGTATTTC ACACCGCATC AGGTGGCACT TTTCGGGGAA ATGTGCGCGG
     3601 AACCCCTATT TGTTTATTTT TCTAAATACA TTCAAATATG TATCCGCTCA TGAGATTATC
     3661 AAAAAGGATC TTCACCTAGA TCCTTTTAAA TTAAAAATGA AGTTTTAAAT CAATCTAAAG
     3721 TATATATGAG TAAACTTGGT CTGACAGTTA CCAATGCTTA ATCAGTGAGG CACCTATCTC
     3781 AGCGATCTGT CTATTTCGTT CATCCATAGT TGCCTGACTC CCCGTCGTGT AGATAACTAC
     3841 GATACGGGAG GGCTTACCAT CTGGCCCCAG TGCTGCAATG ATACCGCGAG ACCCACGCTC
     3901 ACCGGCTCCA GATTTATCAG CAATAAACCA GCCAGCCGGA AGGGCCGAGC GCAGAAGTGG
     3961 TCCTGCAACT TTATCCGCCT CCATCCAGTC TATTAATTGT TGCCGGGAAG CTAGAGTAAG
     4021 TAGTTCGCCA GTTAATAGTT TGCGCAACGT TGTTGCCATT GCTACAGGCA TCGTGGTGTC
     4081 ACGCTCGTCG TTTGGTATGG CTTCATTCAG CTCCGGTTCC CAACGATCAA GGCGAGTTAC
     4141 ATGATCCCCC ATGTTGTGCA AAAAAGCGGT TAGCTCCTTC GGTCCTCCGA TCGTTGTCAG
     4201 AAGTAAGTTG GCCGCAGTGT TATCACTCAT GGTTATGGCA GCACTGCATA ATTCTCTTAC
     4261 TGTCATGCCA TCCGTAAGAT GCTTTTCTGT GACTGGTGAG TACTCAACCA AGTCATTCTG
     4321 AGAATAGTGT ATGCGGCGAC CGAGTTGCTC TTGCCCGGCG TCAATACGGG ATAATACCGC
     4381 GCCACATAGC AGAACTTTAA AAGTGCTCAT CATTGGAAAA CGTTCTTCGG GGCGAAAACT
     4441 CTCAAGGATC TTACCGCTGT TGAGATCCAG TTCGATGTAA CCCACTCGTG CACCCAACTG
```

```
     4501 ATCTTCAGCA TCTTTTACTT TCACCAGCGT TTCTGGGTGA GCAAAAACAG GAAGGCAAAA
     4561 TGCCGCAAAA AAGGGAATAA GGGCGACACG GAAATGTTGA ATACTCATAC TCTTCCTTTT
     4621 TCAATATTAT TGAAGCATTT ATCAGGGTTA TTGTCTCATG ACCAAAATCC CTTAACGTGA
     4681 GTTTTCGTTC CACTGAGCGT CAGACCCCGT AGAAAAGATC AAAGGATCTT CTTGAGATCC
     4741 TTTTTTTCTG CGCGTAATCT GCTGCTTGCA AACAAAAAAA CCACCGCTAC CAGCGGTGGT
     4801 TTGTTTGCCG GATCAAGAGC TACCAACTCT TTTTCCGAAG GTAACTGGCT TCAGCAGAGC
     4861 GCAGATACCA AATACTGTTC TTCTAGTGTA GCCGTAGTTA GGCCACCACT TCAAGAACTC
     4921 TGTAGCACCG CCTACATACC TCGCTCTGCT AATCCTGTTA CCAGTGGCTG CTGCCAGTGG
     4981 CGATAAGTCG TGTCTTACCG GGTTGGACTC AAGACGATAG TTACCGGATA AGGCGCAGCG
     5041 GTCGGGCTGA ACGGGGGGTT CGTGCACACA GCCCAGCTTG GAGCGAACGA CCTACACCGA
     5101 ACTGAGATAC CTACAGCGTG AGCTATGAGA AAGCGCCACG CTTCCCGAAG GGAGAAAGGC
     5161 GGACAGGTAT CCGGTAAGCG GCAGGGTCGG AACAGGAGAG CGCACGAGGG AGCTTCCAGG
     5221 GGGAAACGCC TGGTATCTTT ATAGTCCTGT CGGGTTTCGC CACCTCTGAC TTGAGCGTCG
     5281 ATTTTTGTGA TGCTCGTCAG GGGGGCGGAG CCTATGGAAA AACGCCAGCA ACGCGGCCTT
     5341 TTTACGGTTC CTGGCCTTTT GCTGGCCTTT TGCTCACATG TTCTTTCCTG CGTTATCCCC
     5401 TGATTCTGTG GATAACCGTA TTACCGCCTT TGAGTGAGCT GATACCGCTC GCCGCAGCCG
     5461 AACGACCGAG CGCAGCGAGT CAGTGAGCGA GGAAGCGGAA G
//
```

Supplementary Data 15. The full-length sequence of the donor vector, pCR4-Pv.00443#1μH-P2A-ZeoR.

```
LOCUS       pCR4-Pv.00443#1uH-P2A-ZeoR_sequence      4523 bp    DNA     circular UNA 21-11-2017
DEFINITION
FEATURES             Location/Qualifiers
     primer          207..225
                     /label="M13_reverse_primer"
     primer          242..261
                     /label="T3 priming site"
     misc_feature    295..317
                     /feature_type="Zinc finger"
                     /label="Pv.00443-gRNA#1"
     microhomology   318..357
                     /label="Left microhomology of Pv.00443"
     Site            361..426
                     /site_type="restriction site"
                     /label="Linker + DmP2A"
     CDS             427..798
                     /cds_type="ORF"
                     /label="Zeo+/Bleo+ (Stop codon less)"
     microhomology   799..838
                     /label="Right microhomology of Pv.00443"
     misc_feature    complement(839..861)
                     /feature_type="Zinc finger"
                     /label="Pv.00443-gRNA#1"
     primer          complement(896..914)
                     /label="T7_promoter"
     primer          complement(921..937)
                     /label="M13_forward20_primer"
     CDS             1729..2517
                     /cds_type="ORF"
                     /label="KanR/NeoR"
     CDS             complement(2770..3630)
                     /cds_type="ORF"
                     /label="Amp"
     rep_origin      3731..4374
                     /label="pUC origin"
ORIGIN
        1 AGCGCCCAAT ACGCAAACCG CCTCTCCCCG CGCGTTGGCC GATTCATTAA TGCAGCTGGC
       61 ACGACAGGTT TCCCGACTGG AAAGCGGGCA GTGAGCGCAA CGCAATTAAT GTGAGTTAGC
      121 TCACTCATTA GGCACCCCAG GCTTTACACT TTATGCTTCC GGCTCGTATG TTGTGTGGAA
      181 TTGTGAGCGG ATAACAATTT CACACAGGAA ACAGCTATGA CCATGATTAC GCCAAGCTCA
      241 GAATTAACCC TCACTAAAGG GACTAGTCCT GCAGGTTTAA ACGAATTCGC CCTTTTATAA
      301 GATTTTAATT ATGATGGTCA TCATCATAGA GTAGGCAGAA AACATCATTG CCATCATAAT
      361 GGTTCCGGTG CCACAAACTT CAGTTTGTTA AAGCAGGCCG GAGACGTCGA AGAGAACCCC
      421 GGACCGATGG CCAAGTTGAC CAGTGCCGTT CCGGTGCTCA CCGCGCGCGA CGTCGCCGGA
      481 GCGGTCGAGT TCTGGACCGA CCGGCTCGGG TTCTCCCGGG ACTTCGTGGA GGACGACTTC
      541 GCCGGTGTGG TCCGGGACGA CGTGACCCTG TTCATCAGCG CGGTCCAGGA CCAGGTGGTG
      601 CCGGACAACA CCCTGGCCTG GGTGTGGGTG CGCGGCCTGG ACGAGCTGTA CGCCGAGTGG
      661 TCGGAGGTCG TGTCCACGAA CTTCCGGGAC GCCTCCGGGC CGGCCATGAC CGAGATCGGC
      721 GAGCAGCCGT GGGGGCGGGA GTTCGCCCTG CGCGACCCGG CCGGCAACTG CGTGCACTTC
      781 GTGGCCGAGG AGCAGGACTA AAATCTTATA ACAACATCCT AAAGTATTAT AACACCAACC
      841 ATCATAATTA AAATCTTATA AAAGGGCGAA TTCGCGGCCG CTAAATTCAA TTCGCCCTAT
      901 AGTGAGTCGT ATTACAATTC ACTGGCCGTC GTTTTACAAC GTCGTGACTG GGAAAACCCT
      961 GGCGTTACCC AACTTAATCG CCTTGCAGCA CATCCCCCTT TCGCCAGCTG GCGTAATAGC
     1021 GAAGAGGCCC GCACCGATCG CCCTTCCCAA CAGTTGCGCA GCCTATACGT ACGGCAGTTT
     1081 AAGGTTTACA CCTATAAAAG AGAGAGCCGT TATCGTCTGT TTGTGGATGT ACAGAGTGAT
     1141 ATTATTGACA CGCCGGGGCG ACGGATGGTG ATCCCCCTGG CCAGTGCACG TCTGCTGTCA
     1201 GATAAAGTCT CCCGTGAACT TTACCCGGTG GTGCATATCG GGGATGAAAG CTGGCGCATG
     1261 ATGACCACCG ATATGGCCAG TGTGCCGGTC TCCGTTATCG GGGAAGAAGT GGCTGATCTC
     1321 AGCCACCGCG AAAATGACAT CAAAAACGCC ATTAACCTGA TGTTCTGGGG AATATAAATG
     1381 TCAGGCATGA GATTATCAAA AAGGATCTTC ACCTAGATCC TTTTCACGTA GAAAGCCAGT
     1441 CCGCAGAAAC GGTGCTGACC CCGGATGAAT GTCAGCTACT GGGCTATCTG GACAAGGGAA
     1501 AACGCAAGCG CAAAGAGAAA GCAGGTAGCT TGCAGTGGGC TTACATGGCG ATAGCTAGAC
     1561 TGGGCGGTTT TATGGACAGC AAGCGAACCG GAATTGCCAG CTGGGGCGCC CTCTGGTAAG
     1621 GTTGGGAAGC CCTGCAAAGT AAACTGGATG GCTTTCTTGC CGCCAAGGAT CTGATGGCGC
     1681 AGGGGATCAA GCTCTGATCA AGAGACAGGA TGAGGATCGT TTCGCATGAT TGAACAAGAT
     1741 GGATTGCACG CAGGTTCTCC GGCCGCTTGG GTGGAGAGGC TATTCGGCTA TGACTGGGCA
     1801 CAACAGACAA TCGGCTGCTC TGATGCCGCC GTGTTCCGGC TGTCAGCGCA GGGGCGCCCG
     1861 GTTCTTTTTG TCAAGACCGA CCTGTCCGGT GCCCTGAATG AACTGCAAGA CGAGGCAGCG
     1921 CGGCTATCGT GGCTGGCCAC GACGGGCGTT CCTTGCGCAG CTGTGCTCGA CGTTGTCACT
     1981 GAAGCGGGAA GGGACTGGCT GCTATTGGGC GAAGTGCCGG GGCAGGATCT CCTGTCATCT
     2041 CACCTTGCTC CTGCCGAGAA AGTATCCATC ATGGCTGATG CAATGCGGCG GCTGCATACG
     2101 CTTGATCCGG CTACCTGCCC ATTCGACCAC CAAGCGAAAC ATCGCATCGA GCGAGCACGT
     2161 ACTCGGATGG AAGCCGGTCT TGTCGATCAG GATGATCTGG ACGAAGAGCA TCAGGGGCTC
     2221 GCGCCAGCCG AACTGTTCGC CAGGCTCAAG GCGAGCATGC CCGACGGCGA GGATCTCGTC
     2281 GTGACCCATG GCGATGCCTG CTTGCCGAAT ATCATGGTGG AAAATGGCCG CTTTTCTGGA
     2341 TTCATCGACT GTGGCCGGCT GGGTGTGGCG GACCGCTATC AGGACATAGC GTTGGCTACC
     2401 CGTGATATTG CTGAAGAGCT TGGCGGCGAA TGGGCTGACC GCTTCCTCGT GCTTTACGGT
     2461 ATCGCCGCTC CCGATTCGCA GCGCATCGCC TTCTATCGCC TTCTTGACGA GTTCTTCTGA
     2521 ATTATTAACG CTTACAATTT CCTGATGCGG TATTTTCTCC TTACGCATCT GTGCGGTATT
     2581 TCACACCGCA TCAGGTGGCA CTTTTCGGGG AAATGTGCGC GGAACCCCTA TTTGTTTATT
     2641 TTTCTAAATA CATTCAAATA TGTATCCGCT CATGAGATTA TCAAAAAGGA TCTTCACCTA
     2701 GATCCTTTTA AATTAAAAAT GAAGTTTTAA ATCAATCTAA AGTATATATG AGTAAACTTG
     2761 GTCTGACAGT TACCAATGCT TAATCAGTGA GGCACCTATC TCAGCGATCT GTCTATTTCG
     2821 TTCATCCATA GTTGCCTGAC TCCCCGTCGT GTAGATAACT ACGATACGGG AGGGCTTACC
     2881 ATCTGGCCCC AGTGCTGCAA TGATACCGCG AGACCCACGC TCACCGGCTC CAGATTTATC
     2941 AGCAATAAAC CAGCCAGCCG GAAGGGCCGA GCGCAGAAGT GGTCCTGCAA CTTTATCCGC
     3001 CTCCATCCAG TCTATTAATT GTTGCCGGGA AGCTAGAGTA AGTAGTTCGC CAGTTAATAG
     3061 TTTGCGCAAC GTTGTTGCCA TTGCTACAGG CATCGTGGTG TCACGCTCGT CGTTTGGTAT
     3121 GGCTTCATTC AGCTCCGGTT CCCAACGATC AAGGCGAGTT ACATGATCCC CCATGTTGTG
     3181 CAAAAAAGCG GTTAGCTCCT TCGGTCCTCC GATCGTTGTC AGAAGTAAGT TGGCCGCAGT
     3241 GTTATCACTC ATGGTTATGG CAGCACTGCA TAATTCTCTT ACTGTCATGC CATCCGTAAG
     3301 ATGCTTTTCT GTGACTGGTG AGTACTCAAC CAAGTCATTC TGAGAATAGT GTATGCGGCG
     3361 ACCGAGTTGC TCTTGCCCGG CGTCAATACG GGATAATACC GCGCCACATA GCAGAACTTT
     3421 AAAAGTGCTC ATCATTGGAA AACGTTCTTC GGGGCGAAAA CTCTCAAGGA TCTTACCGCT
     3481 GTTGAGATCC AGTTCGATGT AACCCACTCG TGCACCCAAC TGATCTTCAG CATCTTTTAC
     3541 TTTCACCAGC GTTTCTGGGT GAGCAAAAAC AGGAAGGCAA AATGCCGCAA AAAAGGGAAT
     3601 AAGGGCGACA CGGAAATGTT GAATACTCAT ACTCTTCCTT TTTCAATATT ATTGAAGCAT
     3661 TTATCAGGGT TATTGTCTCA TGACCAAAAT CCCTTAACGT GAGTTTTCGT TCCACTGAGC
     3721 GTCAGACCCC GTAGAAAAGA TCAAAGGATC TTCTTGAGAT CCTTTTTTTC TGCGCGTAAT
     3781 CTGCTGCTTG CAAACAAAAA AACCACCGCT ACCAGCGGTG GTTTGTTTGC CGGATCAAGA
     3841 GCTACCAACT CTTTTTCCGA AGGTAACTGG CTTCAGCAGA GCGCAGATAC CAAATACTGT
     3901 TCTTCTAGTG TAGCCGTAGT TAGGCCACCA CTTCAAGAAC TCTGTAGCAC CGCCTACATA
     3961 CCTCGCTCTG CTAATCCTGT TACCAGTGGC TGCTGCCAGT GGCGATAAGT CGTGTCTTAC
     4021 CGGGTTGGAC TCAAGACGAT AGTTACCGGA TAAGGCGCAG CGGTCGGGCT GAACGGGGGG
     4081 TTCGTGCACA CAGCCCAGCT TGGAGCGAAC GACCTACACC GAACTGAGAT ACCTACAGCG
     4141 TGAGCTATGA GAAAGCGCCA CGCTTCCCGA AGGGAGAAAG GCGGACAGGT ATCCGGTAAG
     4201 CGGCAGGGTC GGAACAGGAG AGCGCACGAG GGAGCTTCCA GGGGGAAACG CCTGGTATCT
     4261 TTATAGTCCT GTCGGGTTTC GCCACCTCTG ACTTGAGCGT CGATTTTTGT GATGCTCGTC
     4321 AGGGGGGCGG AGCCTATGGA AAAACGCCAG CAACGCGGCC TTTTTACGGT TCCTGGCCTT
     4381 TTGCTGGCCT TTTGCTCACA TGTTCTTTCC TGCGTTATCC CCTGATTCTG TGGATAACCG
     4441 TATTACCGCC TTTGAGTGAG CTGATACCGC TCGCCGCAGC CGAACGACCG AGCGCAGCGA
     4501 GTCAGTGAGC GAGGAAGCGG AAG
//
```
