# Supplementary material for: Cas9-mediated genome editing reveals a significant contribution of calcium signaling pathways to anhydrobiosis in Pv11 cells
Source: Sci Rep. 2021 Oct 5;11:19698. doi: 10.1038/s41598-021-98905-w (PMC8492635; doi:10.1038/s41598-021-98905-w)
Supplement: Supplementary file 3 — Supplementary Information 3. [file 41598_2021_98905_MOESM3_ESM.pdf]

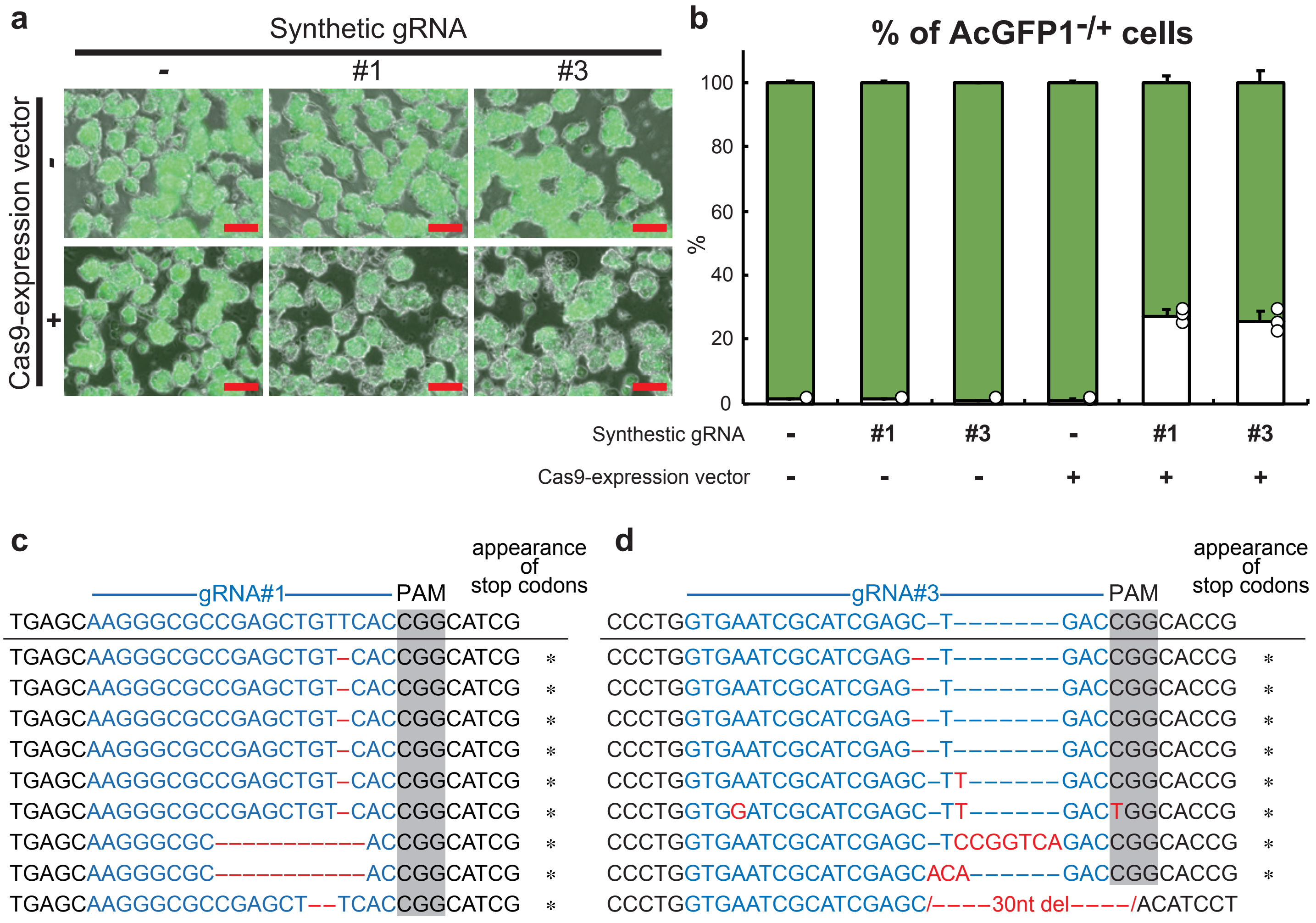

Supplementary Figure 1

The CRISPR/Cas9 system using synthetic gRNAs in Pv11 cells.

a) The Cas9-expression vector and the synthetic gRNAs targeting the AcGFP1 gene were transfected into Pv11-KH cells, and the cells were grown for more than four weeks. b) The proportions of AcGFP1-negative/-positive cells were analyzed by a flow cytometer. White and green colors indicate AcGFP1-negatie/-positive cells, respectively. c, d) The genomic sequences were analyzed in the AcGFP1-negative cells which were transfected with synthetic gRNA #1 (c) or #3 (d). Scale bars, 100 µm. The values are expressed as mean ± SD; n = 3 in each group. Red letters and dashes indicate insertion and deletion mutations, respectively.

Fig. S2

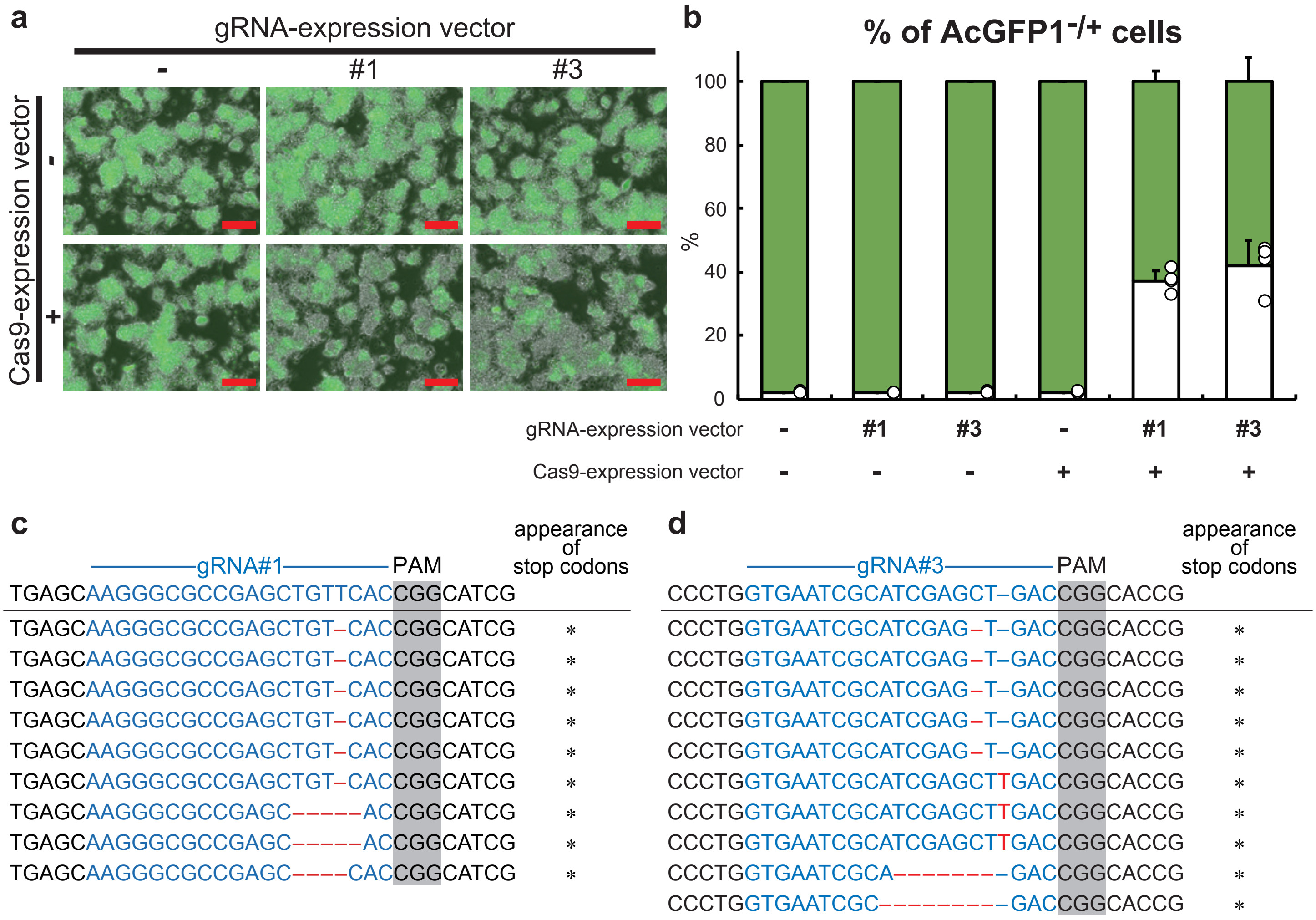

Supplementary Figure 2

The CRISPR/Cas9 system using gRNA-expression vectors in Pv11 cells.

a) The Cas9- and gRNA-expression vectors were transfected into Pv11-KH cells, and the cells were grown for more than four weeks. b) The proportions of AcGFP1-negative/-positive cells were analyzed by a flow cytometer. White and green colors indicate AcGFP1-negatie/-positive cells, respectively. c, d) The genomic sequences were analyzed in AcGFP1-negative cells which were transfected with #1- (c) or #3- (d) gRNA expression vectors. Scale bars, 100 µm. The values are expressed as mean ± SD; n = 4 in each group. Red letters and dashes indicate insertion and deletion mutations, respectively.

Fig. S3

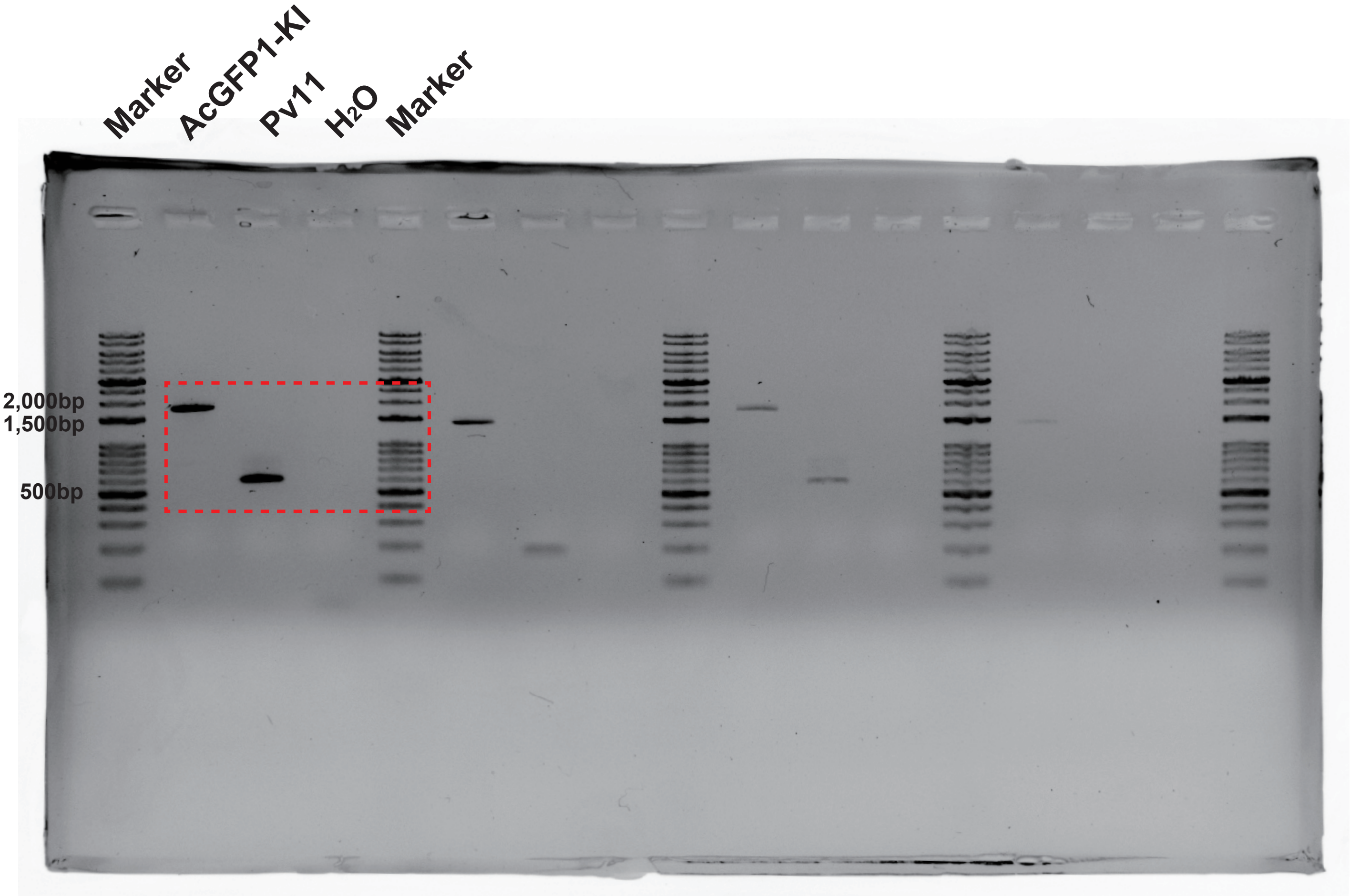

Supplementary Figure 3
Original PCR image in Figure 1c.
The cropped region in Figure 1c is enclosed in red dash line.

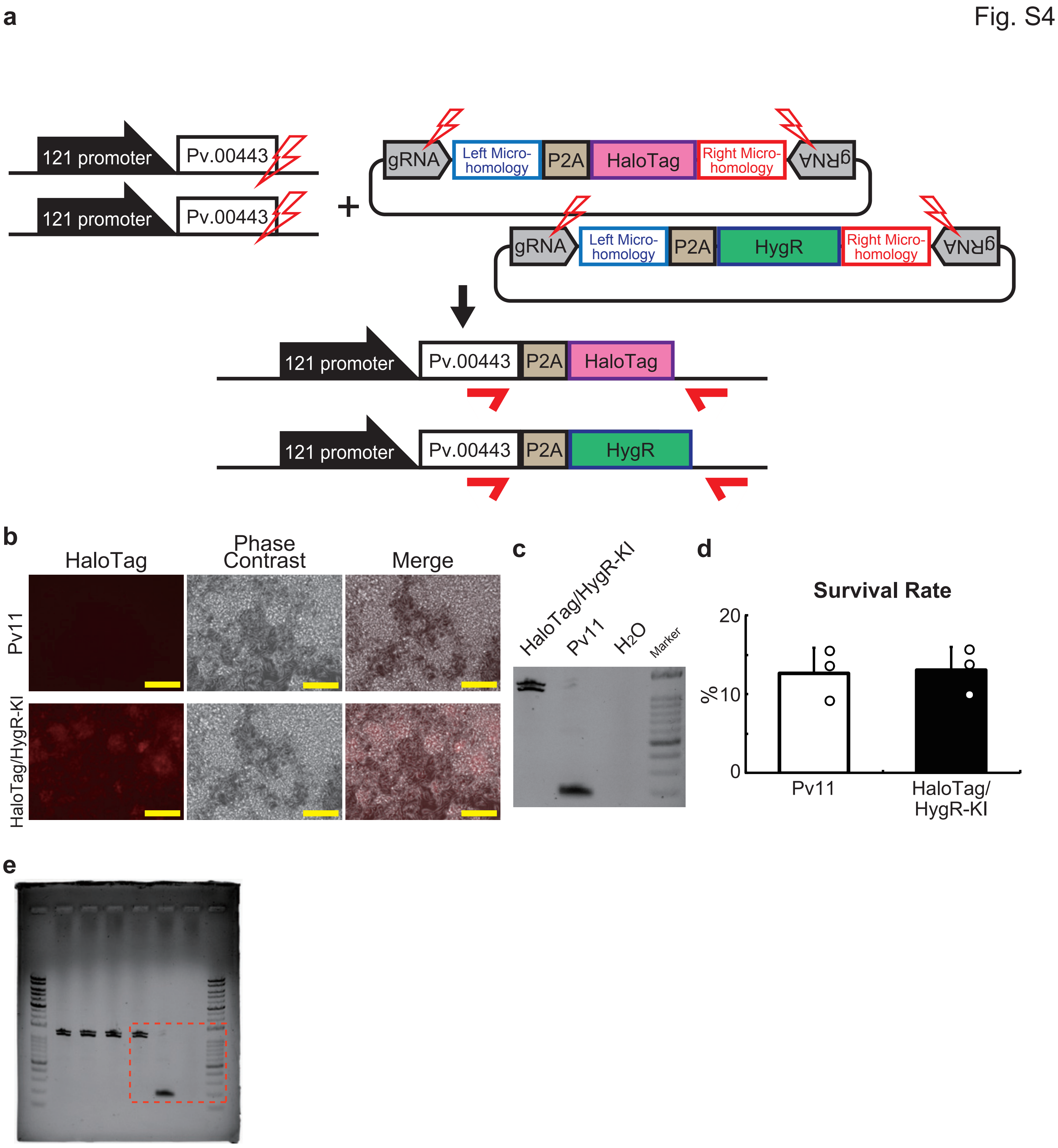

Supplementary Figure S4

The HaloTag or hygromycin resistance (HygR) gene was knocked in into 5' flanking site of the stop codon of *Pv.00443* gene.

a) The scheme of PITCh for HaloTag- or HygR-knock-in is shown; the Cas9- and gRNA-expression vectors and the donor vector harboring HaloTag or HygR gene was transfected into Pv11 cells. Red arrows indicate the primers used in genomic PCR for either intact Pv11 cells or the HaloTag/HygR- KI cells after selection by hygromycin treatment and sorting with fluorescence of HaloTag Ligand. **b)** The images of the cells were acquired by a BZ-X700 microscope. **c)** Genomic PCR in the cell line was carried out. **d)** The survival rate after desiccation-rehydration treatment was compared between Pv11 and HaloTag-/HygR-KI cells. Scale bars, 100 µm. The values are expressed as mean ± SD; n = 3 in each group. **e)** Uncropped original PCR image for the panel d. The cropped region in the panel d is enclosed in red dash line.

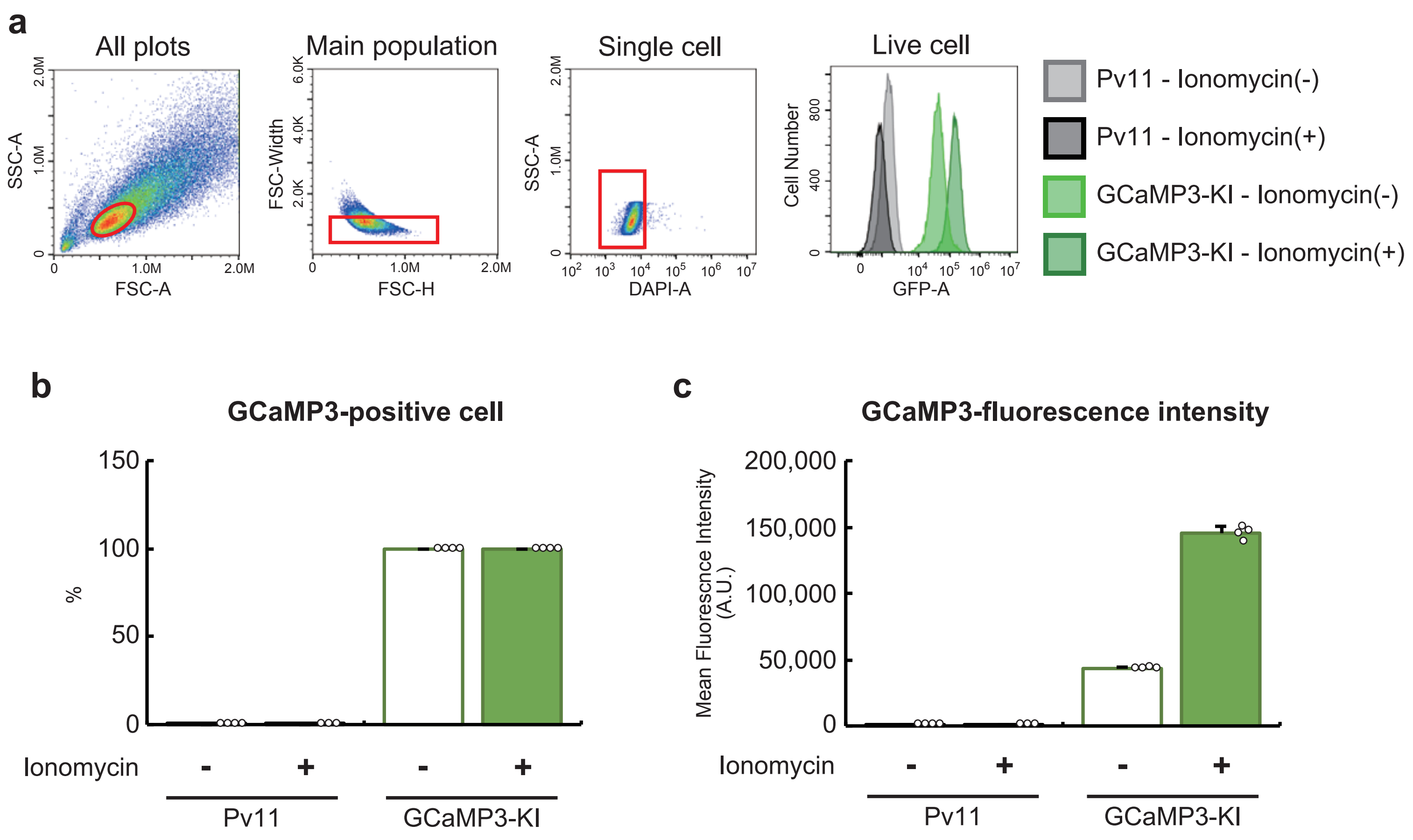

Supplementary Figure 5
Gating hierarchy and dot-plot images of GCaMP3-KI cells in flow cytometry experiments.
a) Representative dot-plot images are shown, and all gates are colored red. The gating hierarchy is main population – single cell – live cell – GCaMP3-positive cell. b) The proportions of GCaMP3-positive cells in the live cell population were analyzed. c) The mean fluorescent intensities in the live cell population were quantified. The values are expressed as mean ± SD; n = 3-4 in each group.

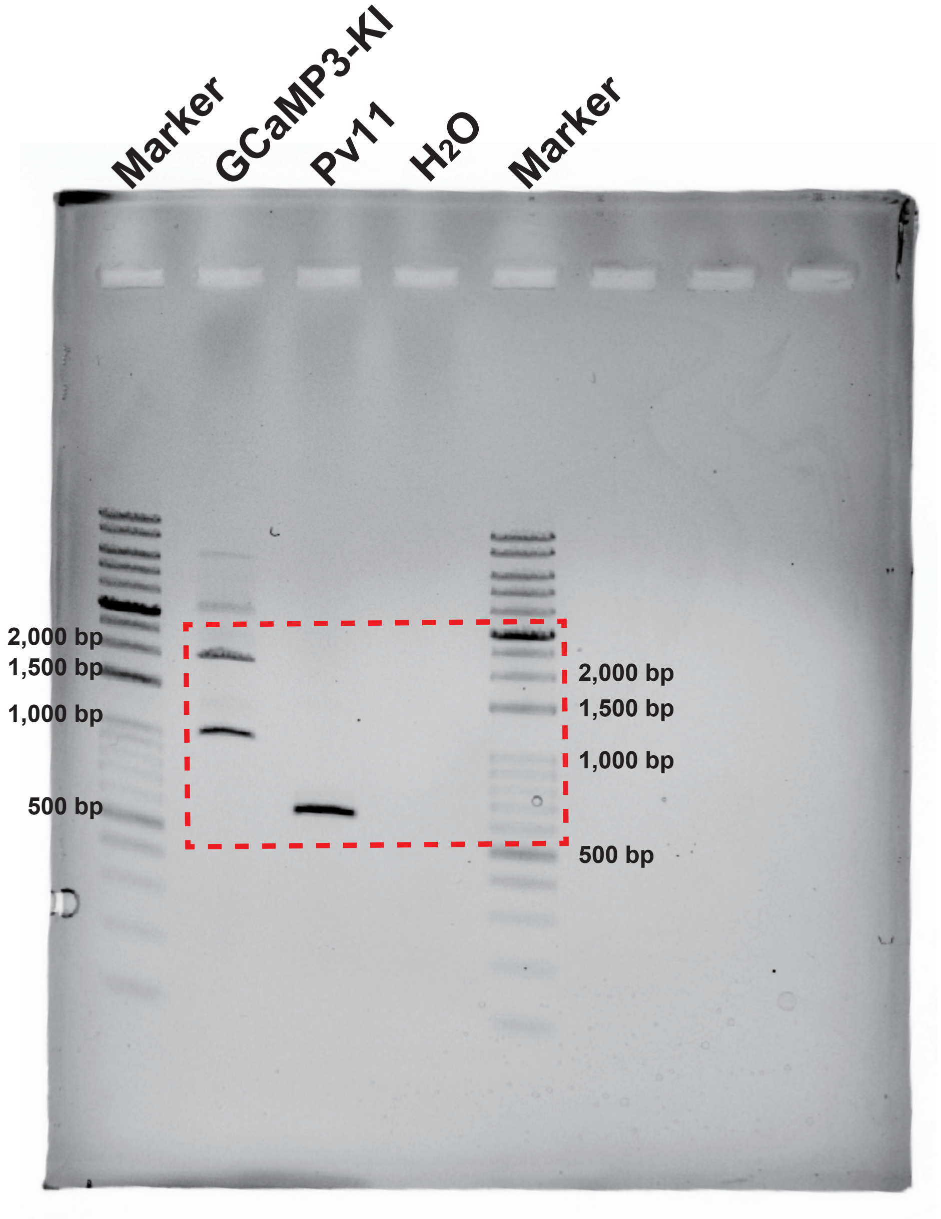

Supplementary Figure 6
Original PCR image in Figure 2c.
The cropped region in Figure 2c is enclosed in red dash line.

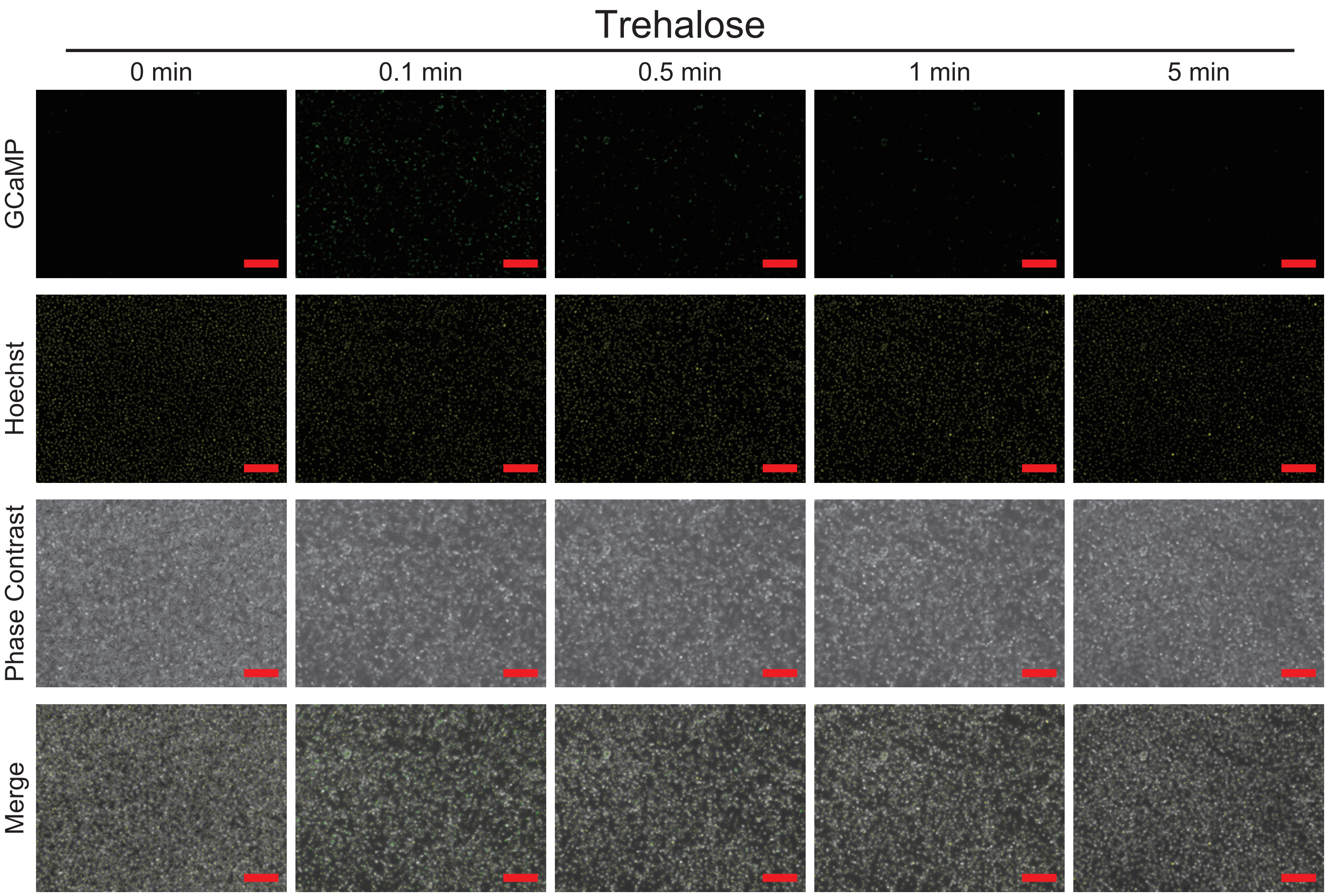

Supplementary Figure 7
Original images of “Trehalose” samples in Figure 3a. Scale bars, 100 μm.

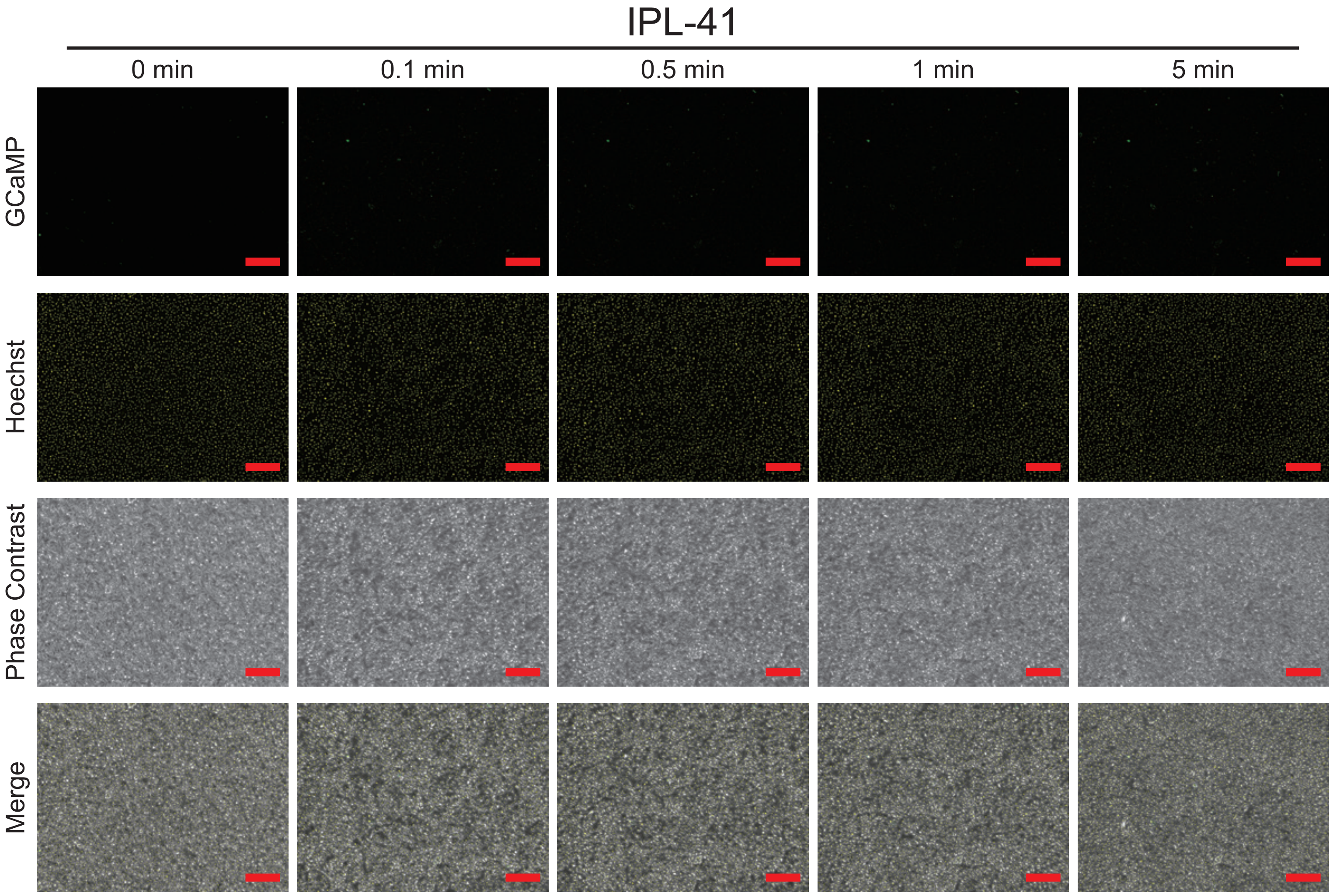

Supplementary Figure 8
Original images of “IPL-41” samples in Figure 3a. Scale bars, 100 μm.

Fig. S9

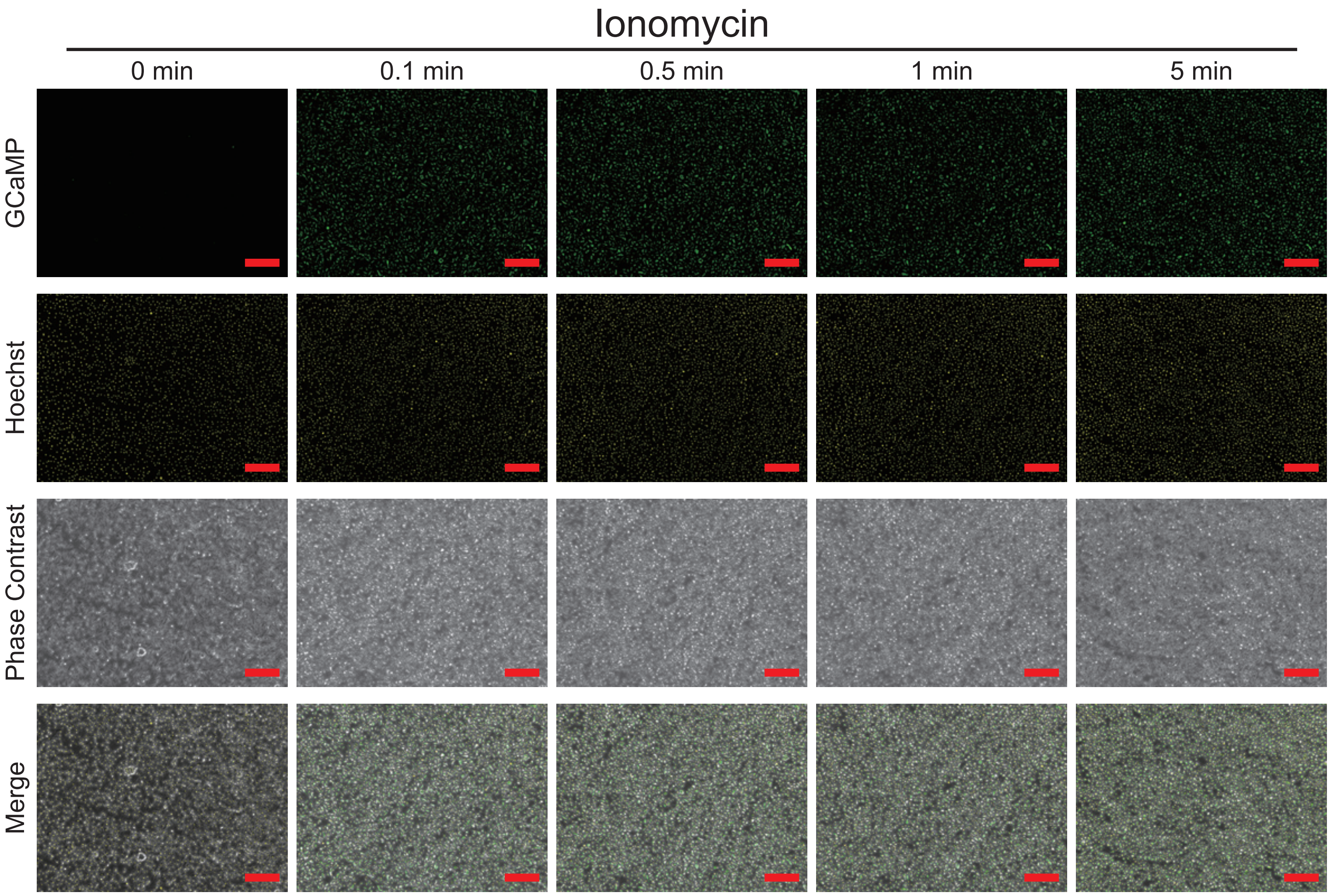

Supplementary Figure 9
Ionomycin treatment in GCaMP3-KI cells.
GCaMP3-KI cells were treated with ionomycin, and the time-course images were acquired. Scale bars, 100 µm.

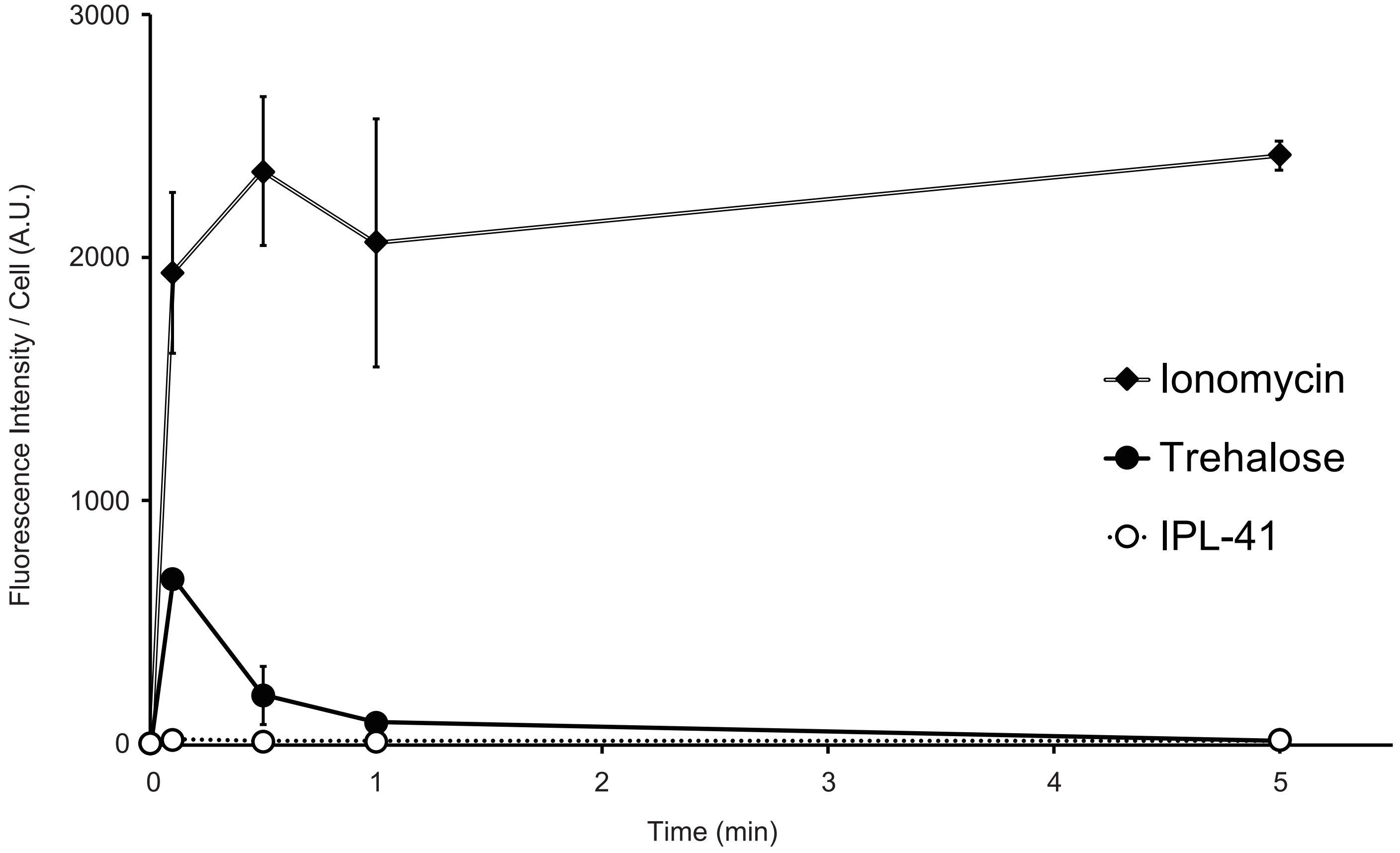

Supplementary Figure 10
Quantitative data of GCaMP3-fluorescence with ionomycin-treated GCaMP3-KI cells. The values are expressed as mean ± SD; n = 3 in each group.

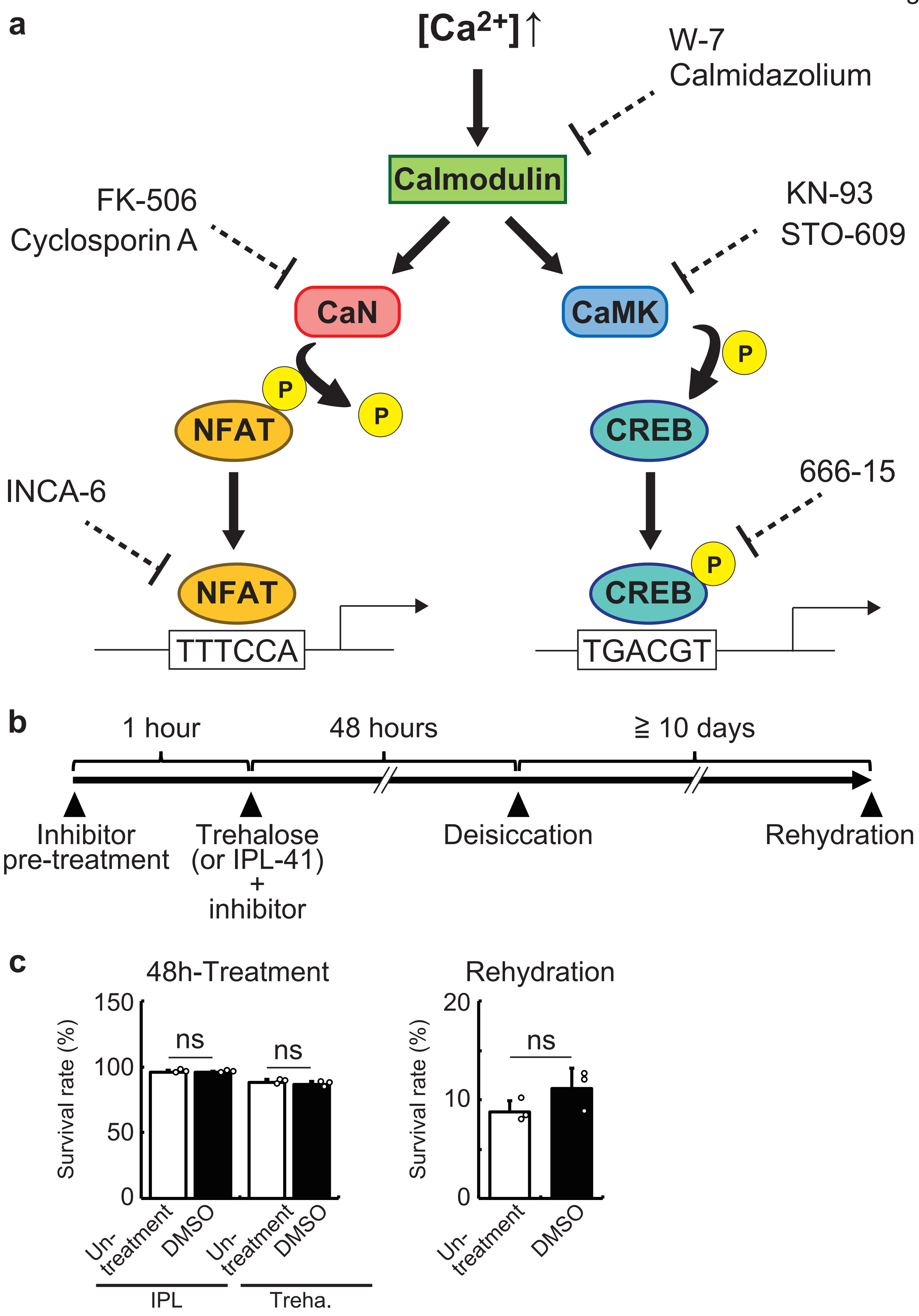

Supplementary Figure 11
Contribution of the calcium signaling pathways to the desiccation tolerance
a) The representative calcium signaling pathways and the inhibitors used in this study were shown. b) The experimental scheme of the inhibitor experiments is shown. c) The effect of DMSO on the survival rates during IPL-41/trehalose incubation or after desiccation-rehydration treatment were analyzed. The values are expressed as mean ± SD; n = 3 in each group.

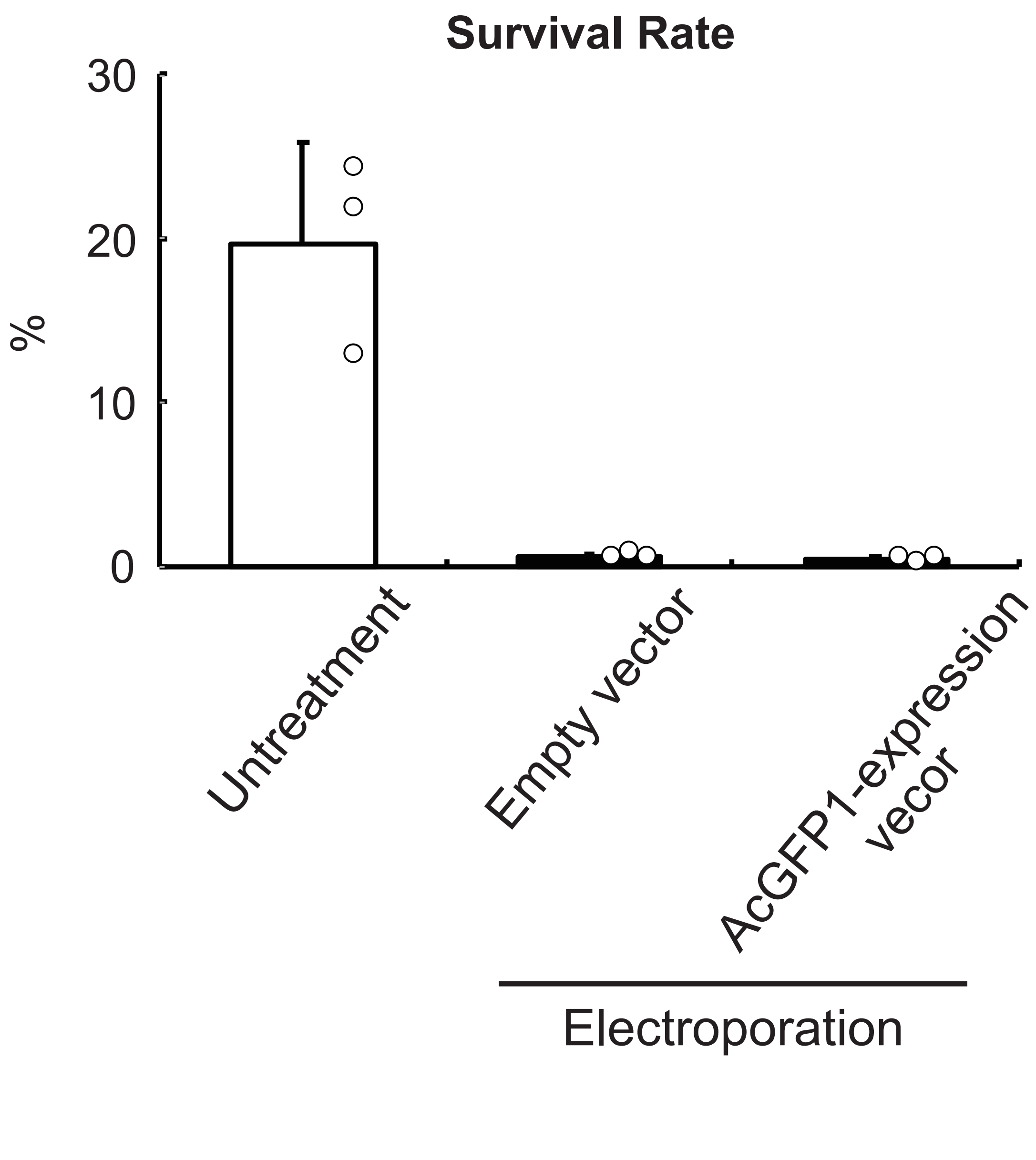

Supplementary Figure 12
Deleterious effect of electroporation on desiccation tolerance in Pv11 cells.
Electroporation was performed two days prior to trehalose treatment and the survival rate after desiccation-rehydration treatment were analyzed.

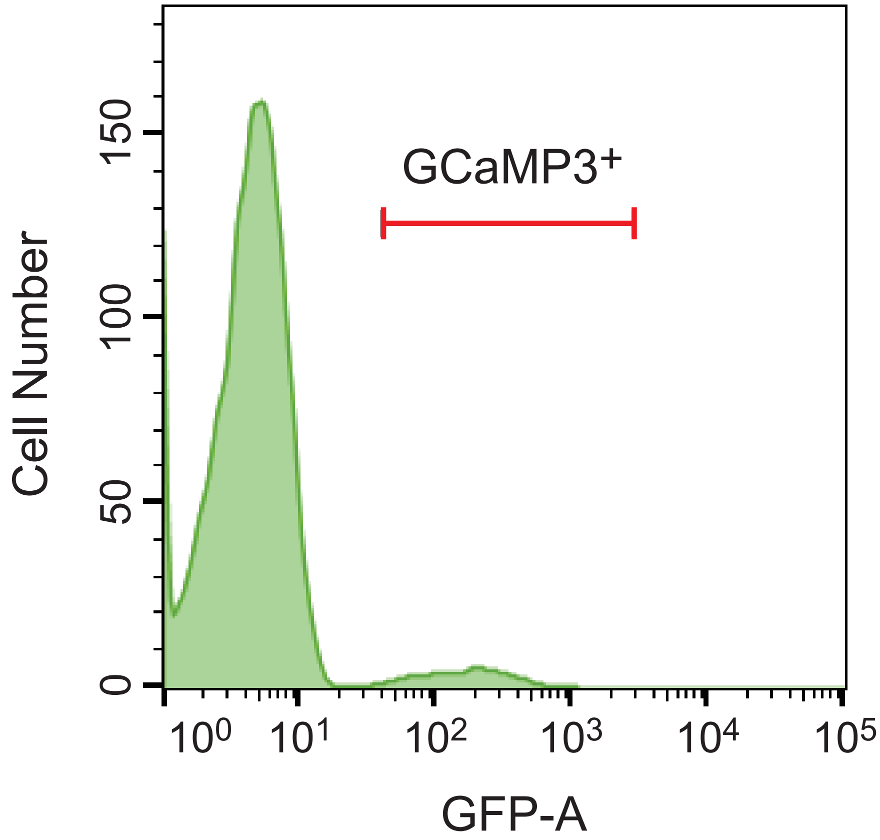

Supplementary Figure 13
Flow cytometric analysis of GCaMP3-KI cells after zeocin treatment.
A histogram for single cell sorting of GCaMP-KI cells is shown. The proportion of GCaMP3-positive cells was 5.1% in the transfected and zeocin-treated cells.
