# Supplementary material for: Cas9-mediated genome editing reveals a significant contribution of calcium signaling pathways to anhydrobiosis in Pv11 cells
Source: Sci Rep. 2021 Oct 5;11:19698. doi: 10.1038/s41598-021-98905-w (PMC8492635; doi:10.1038/s41598-021-98905-w)
Supplement: Supplementary file 4 — Supplementary Information 4. [file 41598_2021_98905_MOESM4_ESM.pdf]

Supplementary Note

This article is based on the manuscript posted to bioRxiv on October 15th in 2020 (10.1101/2020.10.15.340281). Although our article published in *International Journal of Molecular Science* (10.1101/2020.10.15.340281) used the CRISPR technique described in this article, the original idea had already shown in the bioRxiv manuscript. Furthermore, the *IJMS*'s article cites the bioRxiv manuscript. To avoid misunderstanding about the original idea of the CRISPR technique in Pv11 cells, we did not mention the detailed contents of the *IJMS*'s article.
